# “Imaging Triplets on Two Wheels”: ^61^Cu‑, ^64^Cu‑, and ^68^Ga-labeled Bicyclic Peptides Targeting Nectin‑4 Enable High-Contrast PET Imaging of Urothelial Carcinoma and Triple-Negative Breast Cancer

**DOI:** 10.1021/acs.bioconjchem.6c00366

**Published:** 2026-08-22

**Authors:** Johanna Mauersberger, Tobias Krönke, Martin Ullrich, Markus Laube, Florian Brandt, Marc Pretze, Fabian Lohaus, Sebastian Hoberück, Christian Thomas, Matthias Miederer, Ralph A. Bundschuh, Sven Stadlbauer, Klaus Kopka, Jens Pietzsch, Robert Wodtke

**Affiliations:** † 28414 Helmholtz-Zentrum Dresden-Rossendorf, Institute of Radiopharmaceutical Cancer Research, Bautzner Landstraße 400, Dresden 01328, Germany; ‡ 39063 University Hospital Carl Gustav Carus at the Technische Universität Dresden, Klinik Und Poliklinik für Nuklearmedizin, Fetscherstraße 74, Dresden 01307, Germany; § University Hospital Carl Gustav Carus at the Technische Universität Dresden, Klinik Und Poliklinik für Strahlentherapie Und Radioonkologie, Fetscherstraße 74, Dresden 01307, Germany; ∥ University Hospital Carl Gustav Carus at the Technische Universität Dresden, Klinik Und Poliklinik für Urologie, Fetscherstraße 74, Dresden 01307, Germany; ⊥ National Center for Tumor Diseases (NCT), NCT/UCC Dresden, a Partnership Between DKFZ, Faculty of Medicine and University Hospital Carl Gustav Carus, TUD Dresden University of Technology & Helmholtz-Zentrum Dresden-Rossendorf (HZDR), Dresden 01307, Germany; # German Cancer Research Center (DKFZ), Heidelberg 69120, Germany; ∇ German Cancer Consortium (DKTK), Partner Site Dresden, Dresden 01307, Germany; ○ Technische Universität Dresden, School of Science, Faculty of Chemistry and Food Chemistry, Mommsenstraße 4, Dresden 01069, Germany

## Abstract

Nectin-4 is a validated target for theranostic approaches toward urothelial carcinoma, and radiolabeled ligands for PET and SPECT imaging are gaining increasing interest for noninvasive quantification of Nectin-4 prior to a targeted therapy. The goal of this study was to develop bicyclic peptides that allow radiolabeling with three different radionuclides (^61^Cu, ^64^Cu, and ^68^Ga) and to evaluate their suitability for high-contrast PET imaging in urothelial carcinoma (UC) and triple-negative breast cancer (TNBC). For this purpose, **NECT-330**, **NECT-334**, and **NECT-346** were designed based on our previously developed ligand **NECT-224** but harbor additional linker units between the bicyclic scaffold and the radiometal chelator. Binding kinetics to recombinant Nectin-4 and related isoforms were assessed by surface plasmon resonance (SPR) interaction analysis. Nectin-4-specific cell binding of the radiolabeled probes was determined using UC and TNBC cell lines, and PET/CT imaging was performed in respective tumor xenograft models. All novel ligands showed potent and selective binding to Nectin-4, with improved *k*_off_ values compared to **NECT-224**. In small-animal PET/CT imaging studies, the most favorable properties were observed for [^**64**^ **Cu]­Cu-NECT-334** (tumor-to-muscle of 20.8 and tumor-to-heart of 11.2) and [^**64**^ **Cu]­Cu-NECT-346** (tumor-to-muscle of 15.5 and tumor-to-heart of 8.7) 1–2 h postinjection using a HT-1376 tumor model. Although the novel radioligands provided superior imaging properties to the previously developed radioligand [^**64**^ **Cu]­Cu-NECT-224**, a first-in-human application of the latter radioligand was initiated to assess the general suitability of PET/CT imaging in humans with a ^64^Cu-labeled bicyclic peptide directed to Nectin-4. Prospectively, the improved tumor-to-background contrasts achieved with the novel radioligands might further improve diagnostic accuracy and facilitate effective theranostic options in Nectin-4-expressing malignancies.

## Graphical abstract

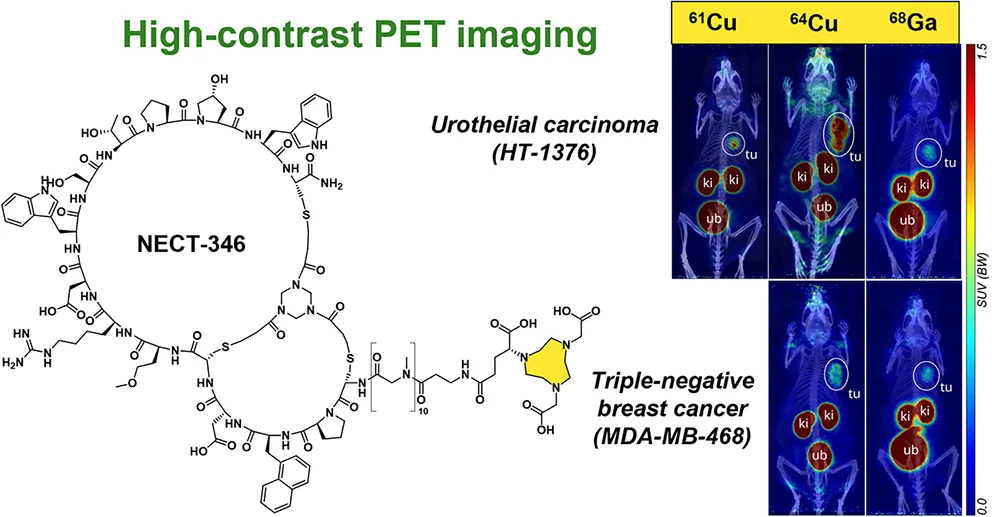

## Introduction

The development of efficient treatment options for cancer patients has witnessed an enormous upswing during the past few years, which can be partly attributed to the success of antibody-drug conjugates (ADCs) as treatment modalities. ADCs can be classified as targeted chemotherapeutic agents consisting of a monoclonal antibody that targets a protein on the surface of tumor cells and a cytotoxic payload that is attached through a linker moiety to the antibody. While the development of the first ADCs dates back more than 50 years, their current success as a treatment modality, best illustrated by the fact that already 15 ADCs have been approved by the FDA by 2025 (13 by the EMA), originates from advances over decades of research into each molecular component and their chemical connection. However, despite their proven efficacy as a treatment modality, it also became clear that the full potential of ADCs can only be unleashed by identifying patient groups that will most likely benefit from their application. In this context, ADCs can also lead to severe side effects, which additionally substantiate the need for patient stratification. For this purpose, quantification of the respective target protein for the ADC in the tumor tissue is required. Considering intratumor heterogeneity and potential differences in target abundance between primary tumors and metastatic lesions, noninvasive molecular imaging using PET and SPECT represents, on top of capturing the actual disease state, a suitable method for target protein quantification.^1–6^

Nectin-4 is a cell surface protein that is involved in Ca^2+^-independent cell adhesion processes. Its primary abundance on tumor cells has rendered Nectin-4 an interesting target for the development of ADCs.^7–10^ The ADC **enfortumab vedotin** (**Padcev**) has been clinically approved by the EMA and the FDA in combination with pembrolizumab (an antibody targeting PD-1) as a first-line treatment in patients with locally advanced or metastatic urothelial carcinoma.^11,12^ Besides urothelial carcinoma, Nectin-4 is also highly abundant in other tumor types, including triple-negative breast and pancreatic cancer,^7^ for which targeted treatment options are largely missing. Consequently, the efficacy of **enfortumab vedotin** is currently also being evaluated in cancer patients with tumor diseases beyond urothelial carcinoma (NCT05915351, pancreatic cancer; NCT04225117, breast cancer). The design of ADCs has also been adapted to other targeting vectors, such as small molecules (SMDCs) and peptides (PDCs).^13–16^ Among peptides as targeting vectors, bicyclic peptides in which the two macrocycles are created by triple-cysteine cross-linking hold great promise for the development of efficient peptide-drug conjugates (denoted as bicycle-toxin conjugates). Due to Bicycle Therapeutics’ proprietary phage screening platform,^17^ bicyclic peptides directed to various target proteins, including Nectin-4, have been identified by this company and optimized regarding binding affinity and stability.^18–21^ For Nectin-4, the most promising bicyclic peptide ligand was then progressed to a bicyclic toxin conjugate (**BT8009**, **zelenectide pevedotin**)^22,23^ that is currently being investigated in a clinical phase III in patients with metastatic urothelial carcinoma (NCT06225596) and has also entered clinical-phase studies for other solid tumor entities (NCT06933329, NCT06840483).

Given the established role of Nectin-4 as a therapeutic target and the clinical need to assess its abundance prior to targeted therapy, interest in radionuclide-based theranostic approaches targeting Nectin-4 has increased.^24–26^ Accordingly, immunoPET and immunoSPECT approaches were followed, including the direct radiolabeling of **enfortumab vedotin**.^27–32^ Furthermore, radiolabeled bicyclic peptides derived from **BT8009** were developed.^33–38^ Some of these radioligands were also advanced up to first-in-human applications with remarkable success, highlighting that Nectin-4 is a valid target for imaging purposes.

Recently, we developed Nectin-4-directed bicyclic peptide ligands derived from **BT8009**, which were labeled with either ^68^Ga or ^64^Cu for PET imaging.^39^ A small library of bicyclic peptides was prepared and characterized regarding their binding kinetics to Nectin-4. Complemented by a detailed radiopharmacological characterization in vitro and in vivo, we discovered that the ligand [^**64**^ **Cu]­Cu-NECT-224** exhibits the most favorable properties in terms of tumor uptake and tumor-to-background ratios. This prompted a first-in-human application of [^**68**^ **Ga]­Ga-NECT-224** as ^68^Ga represents an established radionuclide for clinical imaging, and these first clinical imaging results with [^**68**^ **Ga]­Ga-NECT-224** encourage also the translation of [^**64**^ **Cu]­Cu-NECT-224**.

We herein present data on analogues of **NECT-224**, which have different linker units (PEG_2_, PEG_8_, and Sar_10_) incorporated between the bicyclic peptide and the radiometal-chelator unit. This structural fine-tuning was rationalized based on our previous findings that the chelating unit can have a significant impact on the binding kinetics to Nectin-4 and that, putatively, a more distant separation of the chelating unit from the bicyclic peptide may be advantageous in this context. Furthermore, we hypothesized that hydrophilic linker units may accelerate the washout of the radioligands from healthy tissues without affecting tumor uptake and retention in the tumor tissue, thereby improving the tumor-to-background ratio. This in turn should be beneficial for detecting tumor lesions with even a low abundance of Nectin-4 but should simultaneously support the translation to potential radionuclide therapeutic approaches. The novel Nectin-4 ligands, **NECT-330**, **NECT-334**, and **NECT-346**, were labeled with ^68^Ga and ^64^Cu, and the respective radioligands were comparatively characterized using human urothelial carcinoma (UC) and triple-negative breast cancer (TNBC) cell lines in vitro and by PET imaging using tumor xenograft models derived thereof. As our previous study was focused on urothelial carcinoma models, **[** ^**68**^ **Ga]­Ga-NECT-224** and [^**64**^ **Cu]­Cu-NECT-224** were also characterized herein using the TNBC model. Moreover, these studies were complemented by preclinical imaging studies with [^**61**^ **Cu]­Cu-NECT-224** and [^**61**^ **Cu]­Cu-NECT-346** as ^61^Cu represents another promising PET radionuclide for clinical imaging purposes due to its half-life of 3.4 h,^40^ which better matches the pharmacokinetics of the bicyclic peptide ligands compared to ^64^Cu (*t*_1/2_ = 12.7 h). Overall, all of the novel radioligands may serve as suitable PET imaging probes. However, **NECT-346** labeled with ^61/64^Cu or ^68^Ga represents the most favorable ligand with improved tumor-to-background ratios for all radioligand versions compared to the analogously labeled **NECT-224**. On top of presenting novel Nectin-4-directed radioligands, we investigated the phenomenon of Nectin-4 shedding from the cell surface and showed that, although shedding occurs, the extent is low and does not affect radioligand binding in vitro and putatively also in vivo.

## Results

### Synthesis

The novel Nectin-4 ligands **NECT-330**, **NECT-334,** and **NECT-346** were synthesized via Fmoc-based automated solid-phase peptide synthesis and contain PEG_2_, PEG_8_, and Sar_10_ linkers, respectively, between the bicyclic scaffold and the radiometal-chelator unit (*R*)-NODAGA. All derivatives share the same bicyclic scaffold as **NECT-224** and the same chelating unit (Figure 1). After the synthesis of the linear peptides on resin, followed by their full cleavage, the crude peptides were cyclized in solution using TATA and were subsequently purified by RP-HPLC. Analytical RP-HPLC revealed high chemical purities (≥95% at 230 nm). The isolated yields ranged from 5 to 10%, and the identity of the peptides was confirmed by HRMS (Table S1 in Supporting Information).

**Figure 1:**
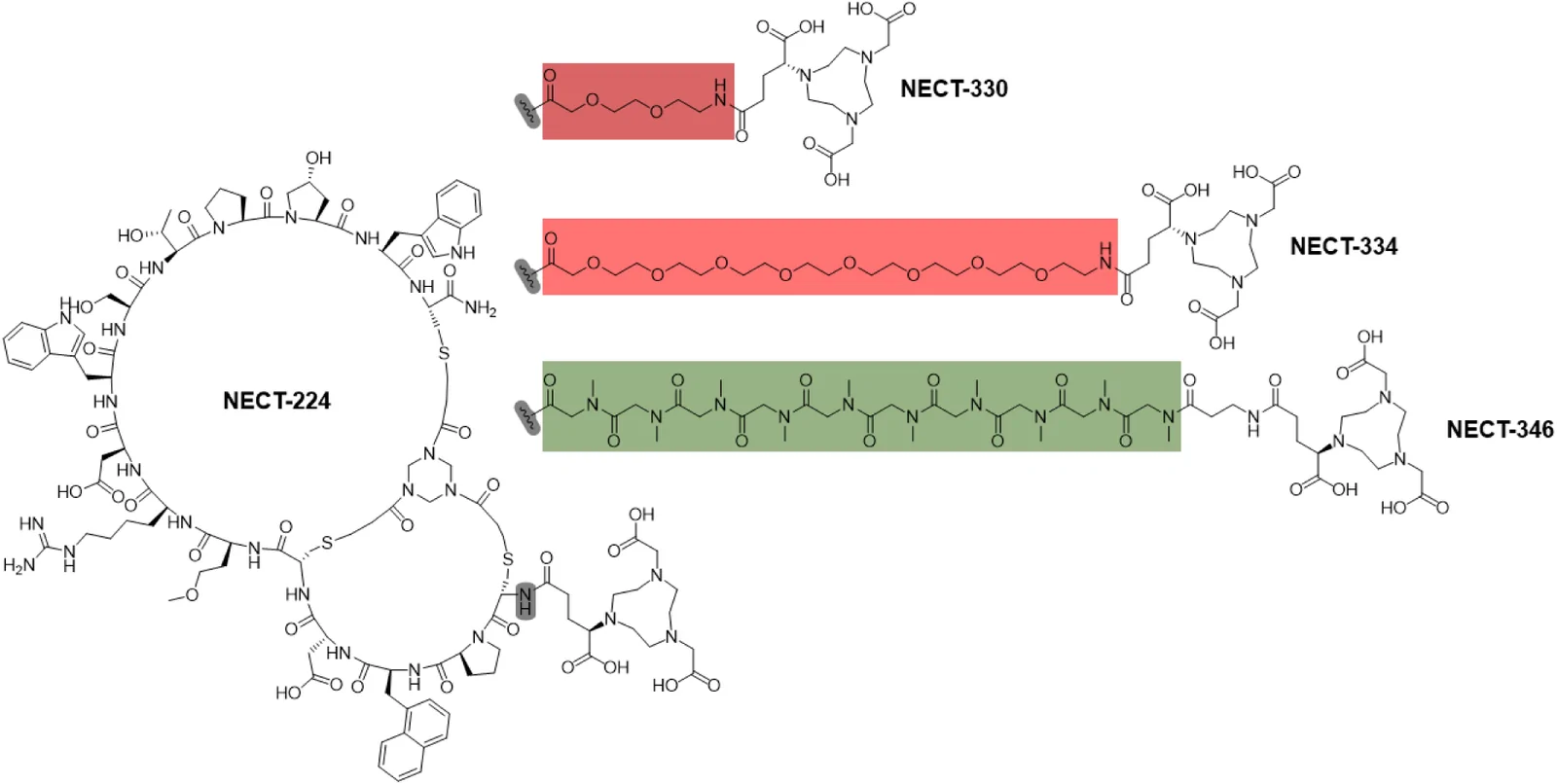
Structures of NECT-330, NECT-334, and NECT-346 in comparison to NECT-224.

### Characterization of Binding Kinetics to Nectin-4

For characterizing the binding kinetics to recombinant human (h) Nectin-4, we used our previously established SPR assay method (Table 1).^39^ In addition to the novel compounds, we sought to determine the binding kinetics of the ^nat^Cu and ^nat^Ga complexes of **NECT-224** and of **NECT-416**, which is the **NECT-224** precursor with a free N-terminus. **NECT-416** showed a threefold lower dissociation rate constant (*k*_off_) compared to **NECT-224** but a similar association rate constant (*k*_on_), resulting in an improved binding affinity (2.04 vs 5.17 nM). A similar trend in *k*_on_ and *k*_off_ values was obtained for ^**nat**^ **Cu-NECT-224** and ^**nat**^ **Ga-NECT-224** compared with **NECT-224**. Moreover, lower *k*_off_ values were also determined for **NECT-330**, **NECT-334**, and **NECT-346** compared to **NECT-224** with a trend toward higher *k*_off_ values with increasing linker length. Besides hNectin-4, the ligand-binding properties toward the other human Nectin isoforms (hNectin-1, -2, and -3) as well as toward mouse (m) Nectin-4 were assessed. At 200 nM, no binding to hNectin-1, -2, and -3 could be detected (Figure S1). The binding kinetics to mNectin-4 were comparable to those toward hNectin-4, albeit lower *K*_d_ values due to higher *k*_on_ value were determined (Table 1).

**Table 1:** Summary of Binding Data of the Bicyclic Peptides to Recombinant Human and Mouse Nectin-4^a^

	*N*-terminal substituent	Linker	Nectin-4	*K*_d_ (SPR, nM)^c^	*k*_on_ (SPR, ×10^6^ M^–1^s^–1^)^c^	*k*_off_ (SPR, ×10^–3^ s^–1^)^c^
NECT-224	NODAGA	-	human	5.17	1.40 (0.22)	7.24 (1.19)
			mouse	0.74	6.01 (0.16)	4.47 (0.18)
NECT-416	NH_2_	-	human	2.04	1.30 (0.02)	2.65 (0.11)
			mouse	0.62	7.84 (0.45)	4.87 (0.49)
^**nat**^ **Cu-NECT-224**	NODAGA	-	human	1.91	1.57 (0.03)	3.00 (0.02)
			mouse	n.d.	n.d.	n.d.
^**nat**^ **Ga-NECT-224**	NODAGA	-	human	1.41	1.28 (0.08)	1.80 (0.12)
			mouse	n.d.	n.d.	n.d.
NECT-330	NODAGA	PEG_2_	human	2.03	0.86 (0.04)	1.75 (0.10)
			mouse	0.68	4.40 (0.09)	2.97 (0.03)
NECT-334	NODAGA	PEG_8_	human	3.04	0.98 (0.01)	2.98 (0.14)
			mouse	1.16	2.67 (0.08)	3.09 (0.08)
NECT-346	NODAGA	Sar_10_	human	4.41	0.74 (0.01)	3.28 (0.06)
			mouse	1.90	2.11 (0.02)	3.99 (0.04)

a Binding data (*K*_d_, *k*_on_, and *k*_off_) determined by SPR. Data shown are mean values (±SD) of at least three separate experiments. Data for **NECT-224** toward human Nectin-4 were previously published by us^39^ and are included here for comparison.

### Radiolabeling, logD_7.4_ Determination, and Plasma Stability

Labeling of **NECT-330**, **NECT-334**, and **NECT-346** with ^68^Ga (NaOAc buffer, pH 4.5, 90 °C, 10 min) at apparent molar activities between 10 and 22 GBq/μmol, and with ^61^Cu/^64^Cu (NH_4_OAc buffer, pH 5.6, 60 °C, 20 min) at an apparent molar activity of 25 GBq/μmol, was performed as previously described.^39^ Radiochemical purity for all radiolabeled peptides exceeded 95% as determined by analytical radio-HPLC. For the ^64^Cu-labeled peptides, logD_7.4_ values of −2.68 ± 0.01 for **NECT-330**, −2.89 ± 0.06 for **NECT-334**, and −2.84 ± 0.15 for **NECT-346** were determined. In contrast, the logD_7.4_ value of [^**68**^ **Ga]­Ga-NECT-346** (−2.57 ± 0.09) was slightly higher compared to its ^64^Cu-labeled analog. All ^64^Cu-labeled peptides were assessed for their stability in human plasma over 24 h by analytical RP-HPLC, which revealed no evidence for proteolytic degradation or radiometal release (Figure S2).

### Characterization of Nectin-4 Concentration in TNBC Cell Lines, Tumor Xenograft Models, and Normal Organs

Previously, we characterized the Nectin-4 abundance in urothelial carcinoma cell lines and tumor xenograft models derived thereof.^39^ For the present study, we conducted similar experiments using selected TNBC cell lines. Western blot analysis and immunofluorescence staining confirmed the presence of Nectin-4 in MDA-MB-468 cells, while no Nectin-4 was detected in MDA-MB-231 cells (Figure 2A/B). Quantification of Nectin-4 was performed with ELISA. Accordingly, a concentration of ∼133 fmol/mg was determined in the MDA-MB-468 monolayer culture and ∼150 fmol/mg in the corresponding xenograft tumor lysates. In contrast, the Nectin-4 levels in the MDA-MB-231 monolayer culture, as well as in the corresponding xenograft tumor lysates, were below the detection limit (Figure 2C). In lysates of the salivary glands, kidneys, and liver from NMRI-nu/nu mice, mNectin-4 levels of 78, 266, and 1070 fmol/mg, respectively, were measured (Figure 2C).

**Figure 2:**
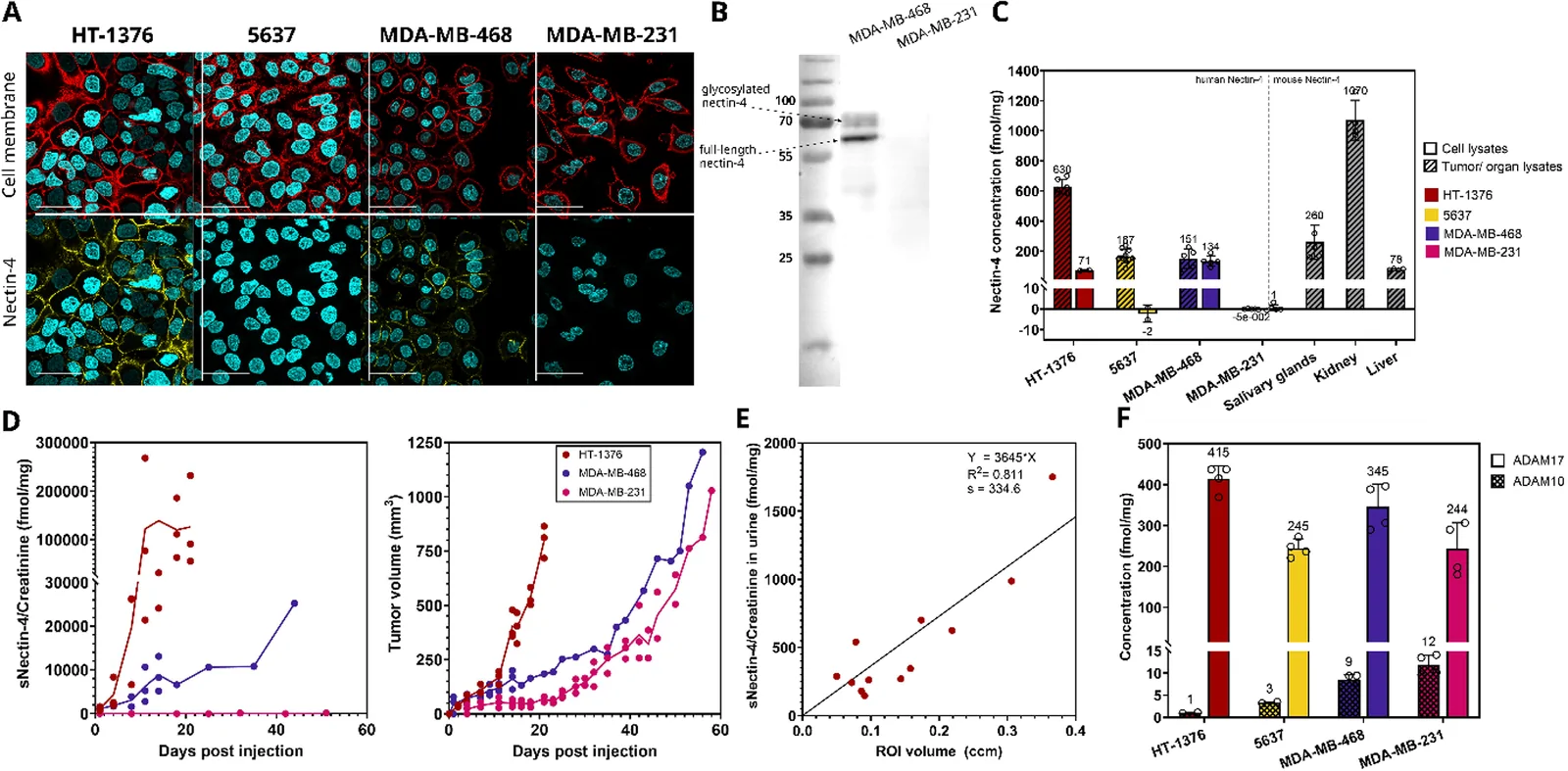
Concentration of (s)­Nectin-4 in cell lines, corresponding xenograft tumors, normal organs, and cell supernatants. **A**) Immunofluorescent staining of HT-1376, 5637, MDA-MB-468, and MDA-MB-231 cells for Nectin-4 (yellow). For reference, the cell membranes were stained with WGA-647 (red) and the cell nuclei with Hoechst (cyan). Scale bars indicate 50 μm. Immunofluorescene staining for HT-1376 and 5637 was previously published by us, but the results were validated herein.^39^ **B**) Exemplary immunoblots of MDA-MB-468 and MDA-MB-231 cell lysates. The Thermo Scientific PageRuler Plus Prestained Protein Ladder was acquired with white-light illumination and automatically merged to the chemiluminescent image (imager Celvin S). **C**) Quantification of Nectin-4 in cell lysates (solid bars) and xenograft tumor lysates (hatched bars), as well as in lysates of normal organs (hatched bars), with ELISA. Data shown are mean values (±SD) of 5 to 12 replicates. Data for HT-1376 and 5637 were previously reported by us, but more replicates were performed herein.^39^ **D**) sNectin-4 levels in urine samples of xenograft tumor-bearing mice as quantified by ELISA depending on the time after tumor cell injection (left diagram). Plot of tumor volume as a function of the time after tumor cell injection (right diagram). Three animals were monitored per tumor model. **E**) Correlation of sNectin-4 detected in urine samples of HT-1376 tumor-bearing mice and corresponding ROI tumor volume as determined by PET imaging. **F**) Quantification of ADAM10 and 17 with ELISA in cell lysates and tumor lysates. Data shown are mean values (±SD) of four replicates.

### Characterization of Nectin-4 Shedding

Because of the known phenomenon of being shed from the cell surface by the metalloproteases ADAM10 and 17,^41–43^ we sought to assess Nectin-4 shedding from the tumor cells investigated herein. For this purpose, we initially quantified soluble Nectin-4 (sNectin-4) in the cell supernatant of HT-1376 cells at different time points (2, 6, 24, and 48 h) with ELISA. sNectin-4 was detected in the cell supernatant and a linear increase of sNectin-4 over time could be derived with an estimated shedding rate of 0.772 fmol per hour (Figure S3A). This value was then used to calculate the amount of sNectin-4 in relation to the amount of cell-bound Nectin-4 in the corresponding cell monolayer. Accordingly, the sNectin-4 amount in the supernatant accounted for less than 2% of the cell-bound Nectin-4 after 1 h of cultivation (Figure S3B).

The relationship between tumor growth and urinary sNectin-4 levels was examined in three tumor models with different Nectin-4 tissue concentrations. The level of sNectin-4 in the urine was measured with ELISA and plotted against time (Figure 2D). While a rapid increase of sNectin-4 up to ∼130,000 fmol/mg creatinine within 21 days of tumor injection was detected in HT-1376 tumor-bearing mice, the MDA-MB-468 tumor-bearing mice showed only a moderate increase of up to ∼25,000 fmol/mg creatinine within 44 days of growth. For all MDA-MB-231 tumor-bearing mice, the sNectin-4 level was below the detection level, even 51 days post-tumor-cell injection. The three xenograft models exhibited an exponential tumor growth curve (Figure 2D) and were monitored until the tumor volume reached ∼1,000 mm^3^ or other termination criteria were reached. The sNectin-4 levels in the urine of HT-1376 tumor-bearing mice that underwent PET studies were also correlated with the tumor ROI volume as determined by image analysis. The plot is shown in Figure 2E and reveals a linear relation, confirming the Nectin-4–dependent radioligand accumulation in the tumor tissue.

Cell lysates were also tested for the presence of ADAM10 and 17 (Figure 2F). Quantification with ELISA revealed levels of 244 to 415 fmol/mg for ADAM17 and 1 to 12 fmol/mg for ADAM10 in the urothelial (HT-1376, 5637) and TNBC cell lines (MDA-MB-468 and -231). Furthermore, incubation of HT-1376 cells with the ADAM10/17 inhibitors **JG26** and **INCB3619** led to a decrease in sNectin-4 in the cell culture supernatant of HT-1376 cells (Figure S3C/D), corroborating the role of these enzymes in shedding of surface-bound Nectin-4. Inhibitor **INCB3619** exhibited a lower effective dose for blocking Nectin-4 shedding and was therefore chosen for assessing the influence of ADAM10/17 inhibition on binding of [^**64**^ **Cu]­Cu-NECT-224** to HT-1376 cells. At 100 nM of **INCB3619** (15 min preincubation period), the specific binding of the radioligand after an incubation period of 1 h was not affected (Figure S3E).

### Characterization of Nectin-4-dependent Radioligand Binding to UC and TNBC Cells

Cell binding at 10 nM of radioligand confirmed the specificity of all ^64^Cu-labeled ligands toward Nectin-4, as no binding to the Nectin-4-negative cell lines (5637 for UC and MDA-MB-231 for TNBC) was detected (Figure 3A,B). Values for specific binding of the ^64^Cu-labeled ligands toward MDA-MB-468 cells were comparable (ranging from 100 to 185 fmol/mg) to [^**64**^ **Cu]­Cu-NECT-224** (134 fmol/mg), while toward HT-1376 cells, their binding was higher compared to the binding of [^**64**^ **Cu]­Cu-NECT-224** (>310 vs 165 fmol/mg). Toward HT-1376 cells, fractions of 75 and 76% for [^**64**^ **Cu]­Cu-NECT-330** and [^**64**^ **Cu]­Cu-NECT-346** were still retained after washing with an acidic glycine buffer, while only 39% was retained for [^**64**^ **Cu]­Cu-NECT-334**. A similar tendency was observed toward MDA-MB-468 cells (58% and 46% vs 32%). For comparison, [^**64**^ **Cu]­Cu-NECT-224** showed retained fractions of 73% toward HT-1376 cells and 47% toward MDA-MB-468 cells.

**Figure 3:**
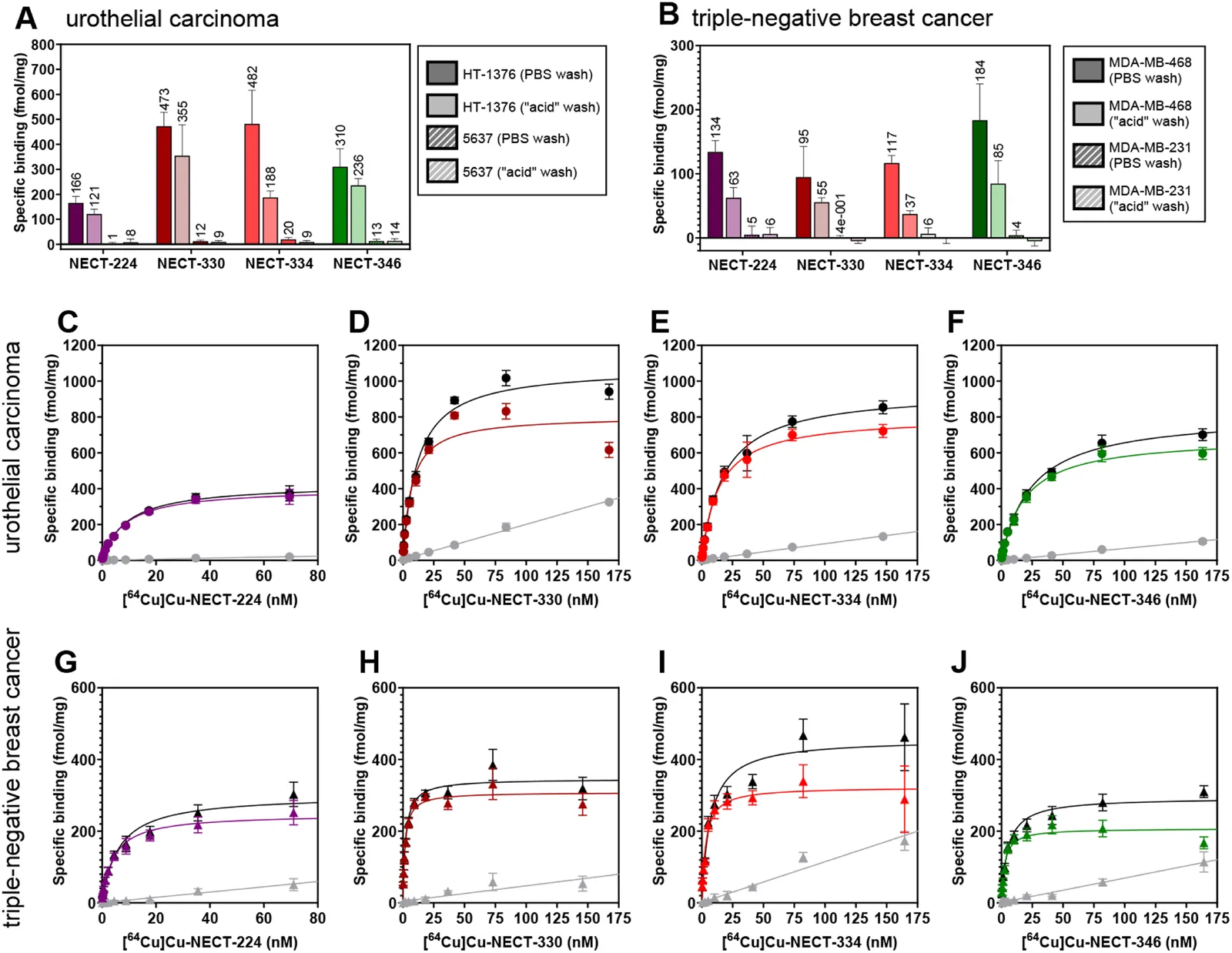
Cell binding and internalization of the ^64^Cu-labeled ligands using UC and TNBC cells. **A/B**) Specific cell binding of the ^64^Cu-labeled ligands to intact UC cells (**A**, HT-1376 in solid bars and 5637 in hatched bars) and TNBC cells (**B**, MDA-MB-468 in solid bars and MDA-MB-231 in hatched bars). Data are shown for specific binding after washing with PBS (muted colors) and after washing with acidic glycine buffer (“acid” wash, light colors), with mean values given on the bars. Data shown are mean values (±SD) of two separate experiments, each performed in sextuplicate. **C–F**) Saturation binding of [**^64^Cu]­Cu-NECT-224** (**C**), [**^64^Cu]­Cu-NECT-330** (**D**), [**^64^Cu]­Cu-NECT-334** (**E**), and [**^64^Cu]­Cu-NECT-346** (**F**) toward intact HT-1376 cells. **G–J**) Saturation binding of [**^64^Cu]­Cu-NECT-224** (**G**), [**^64^Cu]­Cu-NECT-330** (**H**), [**^64^Cu]­Cu-NECT-334** (**I**), and [**^64^Cu]­Cu-NECT-346** (**J**) toward intact MDA-MB-468 cells. In **C**–**J**, data for total binding, nonspecific binding, and specific binding are shown in black, gray, and colored circles and triangles, respectively. Nonspecific binding was determined in the presence of 1 μM **NECT-208** or **NECT-214** (compounds **1e** and **3a** from ref 39, respectively). Regression analysis was performed as described in the Experimental section. Data for [**^64^Cu]­Cu-NECT-224** in **A** and **C** were previously published by us^39^ and are included here for comparison.

In addition to cell binding at 10 nM, saturation binding analyses were performed for the novel ^64^Cu-labeled ligands toward Nectin-4-positive HT-1376 and MDA-MB-468 cells (Figure 3 and Table 2). Apparent binding affinities (*K*_d_ values) were in the low nanomolar range (1.09–16.5 nM), with higher *K*_d_ values being observed toward HT-1376 cells (5.87–16.5 nM) than toward MDA-MB-468 cells (1.09–3.10 nM). Similarly, a lower *K*_d_ value for [^**64**^ **Cu]­Cu-NECT-224** toward the TNBC cell lines was determined compared to the previously determined binding affinity toward HT-1376 (2.11 vs 6.06 nM). While similar *B*_max_ values for the ^64^Cu-labeled ligands, including [^**64**^ **Cu]­Cu-NECT-224,** were determined toward MDA-MB-468 cells (237–308 fmol/mg), higher values were observed for the novel ^64^Cu-labeled ligands **NECT-330**, **NECT-334**, and **NECT-346** compared to [^**64**^ **Cu]­Cu-NECT-224** toward HT-1376 cells. [^**64**^ **Cu]­Cu-NECT-224** exhibited a *B*_max_ value of 378 fmol/mg, as previously determined,^39^ which is 2- to 2.7-fold lower compared to the novel radioligands. Furthermore, a trend toward lower *B*_max_ values with shorter linker length can be noted.

**Table 2:** Summary of Saturation Binding Data for the ^64^Cu- and ^68^Ga-labeled Ligands^c^

		HT-1376^a^		MDA-MB-468^b^	
Compound	radionuclide	*K*_d_ (nM)	*B*_max_ (fmol/mg)	*K*_d_ (nM)	*B*_max_ (fmol/mg)
NECT-224	^64^Cu	6.06 (±0.66)	378 (±12.0)	2.11 (±0.31)	289 (±10)
	^68^Ga	13.1 (±1.34)	1,510 (±58)	3.86 (±1.41)	423 (±25)
NECT-330	^64^Cu	5.87 (±0.93)	1,033 (±25)	1.09 (±0.23)	308 (±7)
NECT-334	^64^Cu	8.56 (±1.10)	911 (±19)	1.76 (±0.35)	267 (±6)
NECT-346	^64^Cu	16.5 (±1.88)	744 (±16)	3.10 (±0.50)	237 (±5)
	^68^Ga	14.5 (±3.05)	756 (±35)	6.62 (±3.54)	177 (±17)

a A Nectin-4 positive urothelial carcinoma cell line.

b A Nectin-4 positive, triple-negative breast cancer cell line.

c Data shown are mean values (±SD) of two separate experiments for ^64^Cu-labeled ligands and one experiment for ^68^Ga-labeled ligands with intact cells, which were performed in quintuplicate. Binding data for **[^64^Cu]­Cu-NECT-224** and **[^68^Ga]­Ga-NECT-224** toward HT-1376 were previously published by us.^39^

In addition to assessing the cell binding of all ^64^Cu-labeled ligands, binding of their ^68^Ga-labeled pendants to TNBC cells was exemplarily determined for [^**68**^ **Ga]­Ga-NECT-224** and [^**68**^ **Ga]­Ga-NECT-346,** as well as for [^**68**^ **Ga]­Ga-NECT-346** toward the UC cells (Table 2 and Figure S4). In contrast to the results for the ^64^Cu-labeled ligands, binding to both UC and TNBC cells was more favorable for [^**68**^ **Ga]­Ga-NECT-224** compared with [^**68**^ **Ga]­Ga-NECT-346**, as exemplified by higher (maximum) binding capacities.

### PET Imaging Using a UC Tumor Xenograft Mouse Model

The distribution of the novel ^64^Cu- and ^68^Ga-labeled Nectin-4 ligands was evaluated in a subcutaneous HT-1376 tumor xenograft model via PET/CT imaging (Figure 4). The time-activity curves (TACs) for tumor and heart are shown in Figures 4C,D and Figure 4H,I (TACs for muscle, kidney, liver, and urinary bladder are shown in the Supporting Information, Figure S5). According to the PET/CT images, all novel ligands labeled either with ^64^Cu or ^68^Ga clearly enabled visualization of the Nectin-4–positive HT-1376 tumors. Furthermore, all radioligands underlay mainly a renal excretion. Apart from the bladder and kidneys, a low activity accumulation was also visible in the salivary glands. The TACs for the heart (blood content only) followed biphasic blood kinetics, with radioligand fractions of 88–93% being removed during the first phase (*T*_1/2_ = 1.2–1.7 min), followed by a second phase (*T*_1/2_ = 26–27 min) for the ^64^Cu-labeled ligands, but similar values were also obtained for the ^68^Ga-labeled ligands (1.5–1.6 min and 27–28 min; Tables S6 and S7).

**Figure 4:**
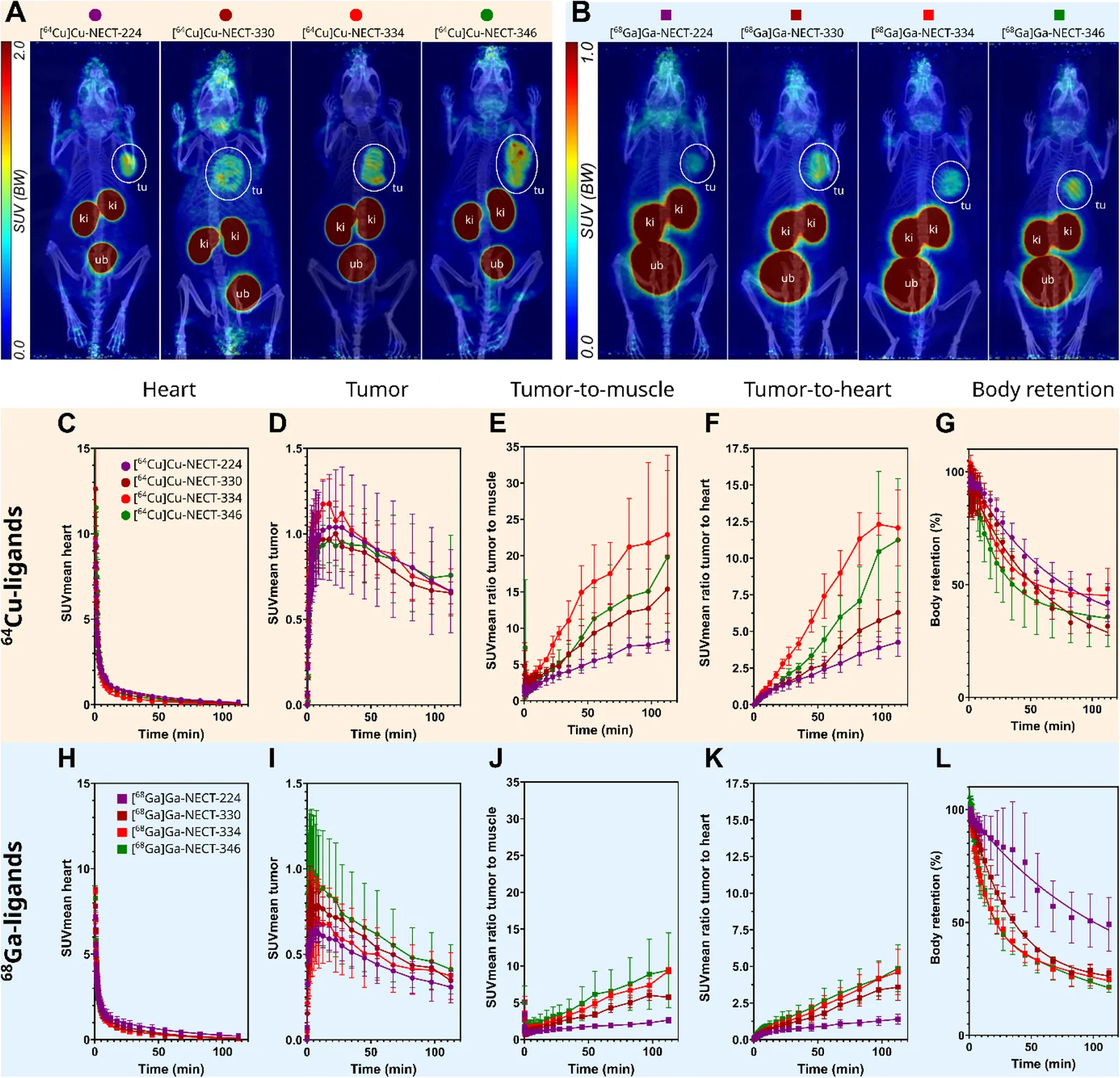
PET images and image-derived uptake values of the ^64^Cu- and ^68^Ga-labeled Nectin-4 ligands in HT-1376 tumor-bearing mice. **A/B**) PET/CT images at 1–2 h after intravenous injection of the indicated ^64^Cu-labeled (**A**) and ^68^Ga-labeled ligands (**B**) in HT-1376 tumor-bearing mice. 6–9 MBq/animal (0.3–0.45 nmol/animal) were injected. Images are presented as maximum-intensity projections with a common scale (SUV 0–2 for ^64^Cu-labeled and SUV 0–1 for ^68^Ga-labeled ligands). Anatomical positions of the tumor (tu), kidney (ki), and urinary bladder (ub) are shown. **C/D/H/I**) Time-activity curves (SUVmean, decay-corrected, as a function of time up to 2 h) for the blood content of the heart (**C/H**) and tumor (**D/I**) obtained from quantitative analysis of PET images are depicted for the ^64^Cu-labeled (**C/D**) and ^68^Ga-labeled ligands (**H/I**). **E/F/J/K**) Time-resolved SUVmean ratios up to 2 h p.i., including tumor-to-muscle (**E/J**) and tumor-to-heart (**F**/**K**) for the ^64^Cu-labeled (**E/F**) and ^68^Ga-labeled ligands (**J/K**). **G/L**) Time-resolved body retention in % of the initial dose up to 2 h p.i. for the ^64^Cu-labeled (**G**) and ^68^Ga-labeled ligands (**L**). Data points in **C–L** represent mean values (±SD) measured in groups of HT-1376 tumor-bearing mice (*n* = 6 for [**^64^Cu]­Cu-NECT-224**, *n* = 4 for [**^64^Cu]­Cu-NECT-334,** and [**^68^Ga]­Ga-NECT-346** or *n* = 3). All data shown for [**^64^Cu]­Cu-NECT-224** were previously published by us^39^ and were included for reasons of comparison.

Among the ^64^Cu-labeled ligands, the TACs for the tumor tissue were very similar and showed fast tumor uptake, with a maximum SUVmean values of 0.9–1.1 at ≈22.5 min p.i. (Figure 4D). In a similar fashion, the TACs for the tumor tissue of the ^68^Ga-labeled ligands resembled strongly each other; however, the maximum SUVmean values (0.6–1.0) were somewhat lower and were reached at an earlier time point (≈5 min p.i.) compared to their ^64^Cu-labeled pendants (Figure 4I). After the respective maximum tumor uptake, all radioligands underwent washout from the tumor tissue, with SUVmean values of 0.7–0.8 and 0.4–0.5 being reached at 1–2 h p.i. for the ^64^Cu-labeled and ^68^Ga-labeled ligands, respectively. At the same time point, the proportions of body retention, i.e., the percentage of the initially injected activity retained in the mouse 2 h p.i., were similar within each subgroup of ^64^Cu- and ^68^Ga-labeled ligands, but lower for the latter radioligands (32–48% vs 21–27%, Figure 4G and L).

In contrast to the similar tumor TACs, the tumor-to-muscle (T/M) and tumor-to-heart (T/H) ratios are more distinct among each group of radioligands with the best tumor site visualization being achieved at the 1–2 h time frame after radioligand injection. In this context, statistical comparisons were performed for the T/M and T/H ratios and are included in Tables S2 and S3. For the ^64^Cu-labeled ligands (Figure 4E and F, Table S2), [^**64**^ **Cu]­Cu-NECT-334** exhibited the highest T/M and T/H ratios at 1–2 h p.i. (20.8 and 11.2), followed by [^**64**^ **Cu]­Cu-NECT-346** (15.5 and 8.7) and [^**64**^ **Cu]­Cu-NECT-330** (12.7 and 5.3). In contrast, for the ^68^Ga-labeled ligands (Figure 4J and K, Table S2), [^**68**^ **Ga]­Ga-NECT-334** and [^**68**^ **Ga]­Ga-NECT-346** showed similar ratios (≈8.0 and ≈4.0 for T/M and T/H, respectively), but these ratios were again higher compared to [^**68**^ **Ga]­Ga-NECT-330** (5.3 and 3.1).

The biodistribution of [^**64**^ **Cu]­Cu-NECT-346** was also characterized using the 5637 tumor xenograft model (Figures S6 and S7), which has a lower Nectin-4 level compared to the HT-1376 xenograft model (Figure 2B). Besides [^**64**^ **Cu]­Cu-NECT-346**, the biodistribution of [^**64**^ **Cu]­Cu-NECT-224** was also assessed in 5637 tumor-bearing mice (Figures S6 and S7 and Table S4), which was not done in our previous study. The shape and height of the tumor TAC, as well as the T/M and T/H ratios for [^**64**^ **Cu]­Cu-NECT-346** in the 5637 model, were comparable to the data obtained with the HT-1376 model using the same radioligand (e.g., T/M ratios of 15.5 for HT-1376 and 17.2 for 5637 at 1–2 h p.i.), but improved tumor uptake and again higher tumor-to-organ ratios were obtained in the 5637 tumor model in comparison to [^**64**^ **Cu]­Cu-NECT-224** (T/M of 7.3 for 5637 at 1–2 h p.i.).

In addition to labeling of the NODAGA-bearing Nectin-4 ligands with ^64^Cu and ^68^Ga, ^61^Cu labeling was tested for **NECT-224** and **NECT-346,** and the ^61^Cu-labeled ligands were radiopharmacologically characterized in vivo using the HT-1376 tumor model (Figures 5, Figure S8, and Table S5). Both radioligands enabled clear visualization of the tumor tissue, with comparable tumor TACs as well as T/M and T/H ratios being reached up to 2 h p.i. (e.g., T/M of 16.0 for [^**61**^ **Cu]­Cu-NECT-346** and 13.1 for [^**61**^ **Cu]­Cu-NECT-224** at 1–2 h p.i.).

**Figure 5:**
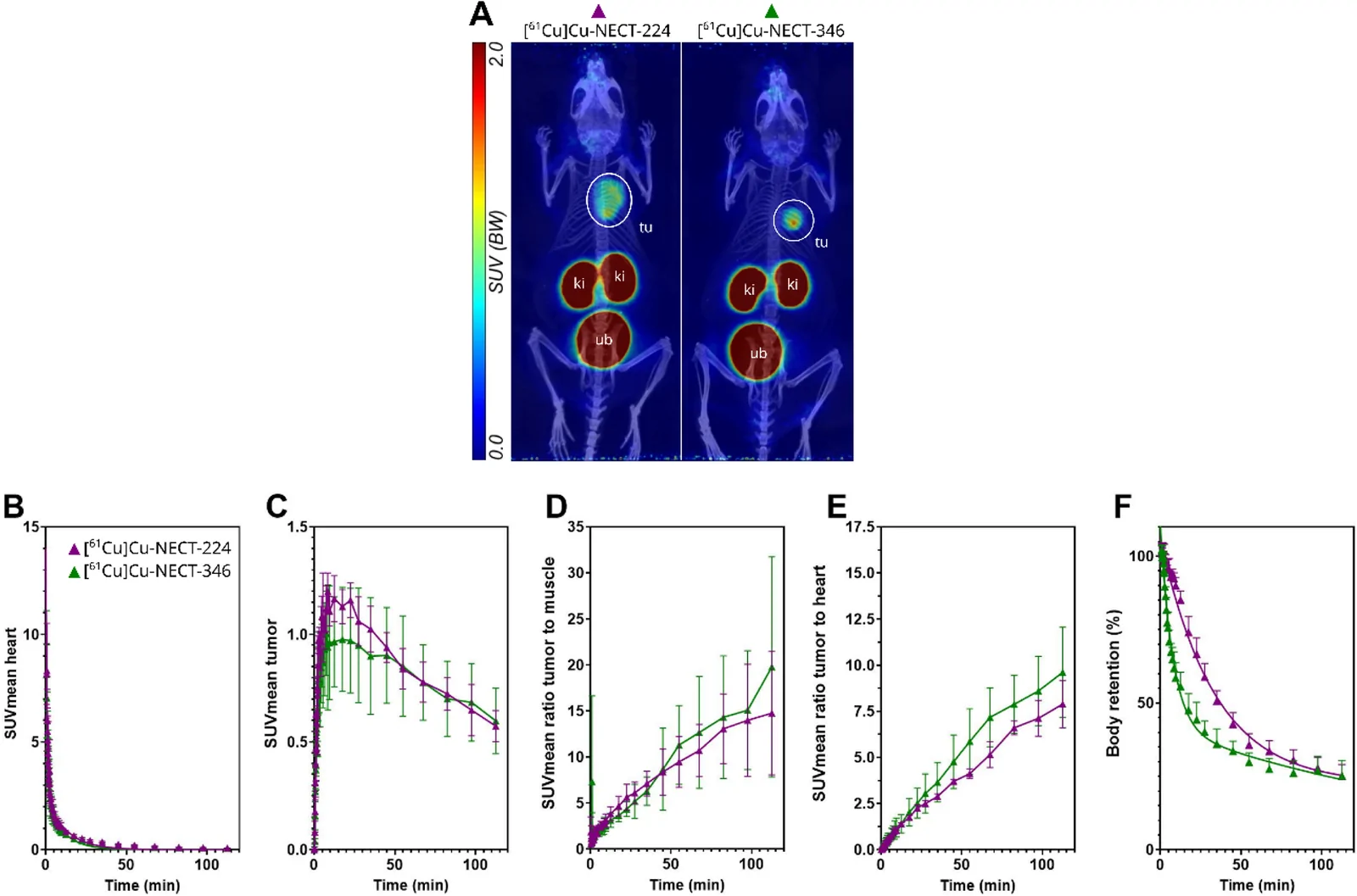
PET images and image-derived uptake values of ^61^Cu-labeled NECT-224 and NECT-346 in HT-1376 tumor-bearing mice. **A**) PET/CT images at 1–2 h after intravenous injection of [**^61^Cu]­Cu-NECT-224** and [**^61^Cu]­Cu-NECT-346** in HT-1376 tumor-bearing mice. 6–9 MBq/animal (0.3–0.45 nmol/animal) were injected. Images are presented as maximum-intensity projections with a common scale. Anatomical positions of the tumor (tu), kidney (ki), and urinary bladder (ub) are shown. **B/C**) Time-activity curves (SUVmean, decay-corrected, as a function of time up to 2 h) for the blood content of the heart (**B**) and tumor (**C**) obtained from quantitative analysis of PET images are depicted. **D/E**) Time-resolved SUVmean ratios up to 2 h p.i., including tumor-to-muscle (**D**) and tumor-to-heart (**E**). **F**) Time-resolved body retention in % of the initial dose up to 2 h p.i. Data points in **B–F** represent mean values (±SD) measured in groups of HT-1376 tumor-bearing mice (*n* = 4).

To complement our in vitro studies on the potential influence of modulating the shedding of Nectin-4, we studied the tumor uptake of [^**64**^ **Cu]­Cu-NECT-224** with preadministration of the ADAM10 and 17 inhibitor **INCB3619** (i.p. injection of 1.4 mg/kg, 17 and 1 h before radiotracer injection). The shape and height of the tumor TAC resembled those observed in the absence of **INCB3619** (Figures S9 and S10).

### PET Imaging Using a TNBC Tumor Xenograft Mouse Model

All novel ^64^Cu-labeled ligands and the previously developed [^**64**^ **Cu]­Cu-NECT-224** were also subjected to PET/CT imaging studies using the Nectin-4-positive MDA-MB-468 tumor model (Figure 6; TACS for normal organs in Figure S11). The tumor tissue could be visualized with all ^64^Cu-labeled ligands, and the general shape of the tumor TACs resembled those obtained for the HT-1376 tumor model. However, the maximum SUVmean values tended to be lower for the MDA-MB-468 compared with the HT-1376 tumor. Furthermore, all novel ^64^Cu-labeled ligands showed higher SUVmean values compared to [^**64**^ **Cu]­Cu-NECT-224**, in particular from time points of 30 min or later after intravenous injection (e.g., SUVmean of 0.3 for [^**64**^ **Cu]­Cu-NECT-224** vs 0.5 for [^**64**^ **Cu]­Cu-NECT-334** at 1–2 h p.i., Figure 6D). The T/M and T/H ratios are significantly higher for [^**64**^ **Cu]­Cu-NECT-330** and [^**64**^ **Cu]­Cu-NECT-346** compared to [^**64**^ **Cu]­Cu-NECT-224** (e.g., a T/M ratio of 6.0 for [^**64**^ **Cu]­Cu-NECT-224** vs 10.0 for [^**64**^ **Cu]­Cu-NECT-346**; Figure 6E,F, Table S3).

**Figure 6:**
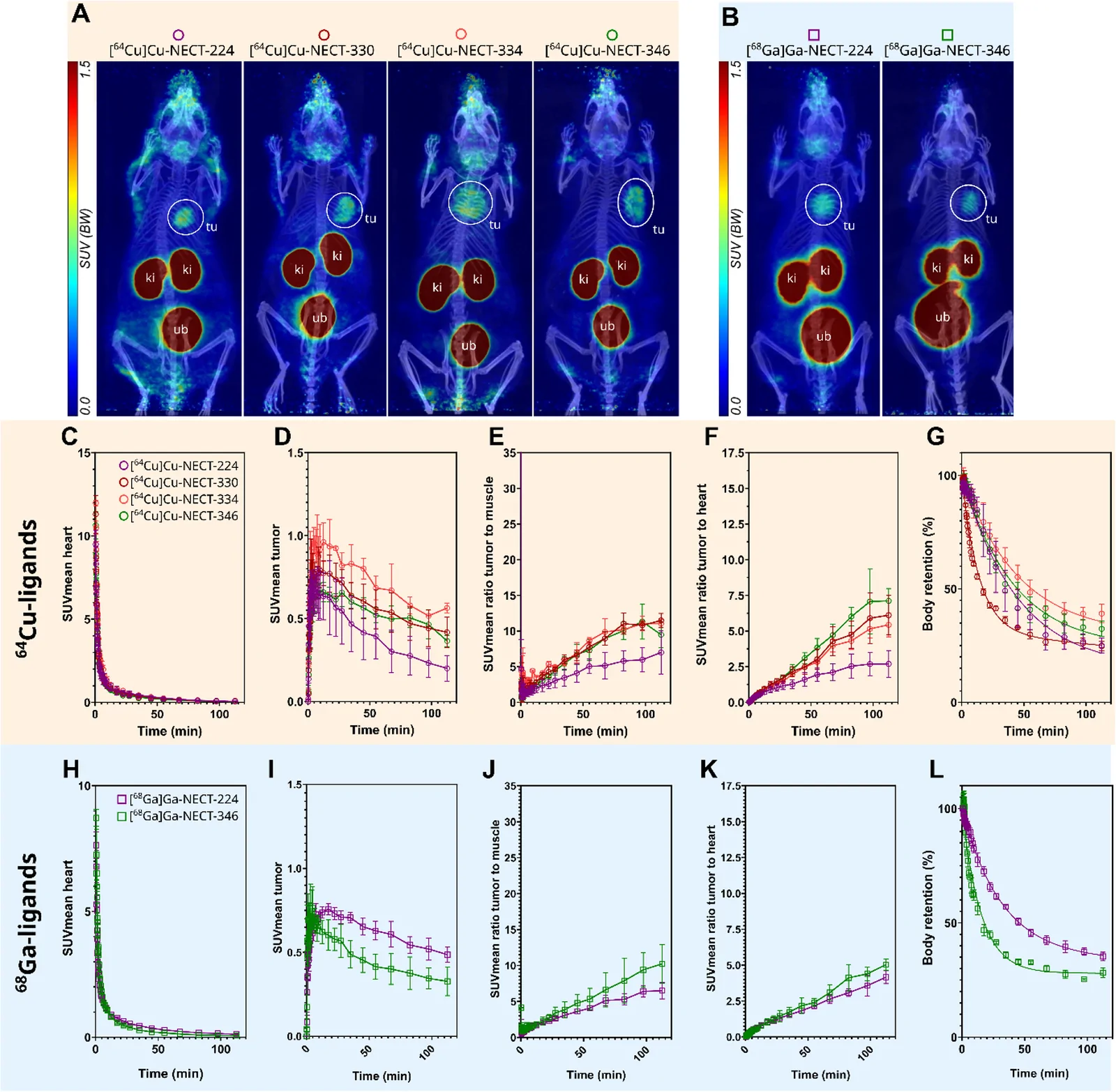
PET images and image-derived uptake values of the ^64^Cu- and ^68^Ga-labeled Nectin-4 ligands in MDA-MB-468 tumor-bearing mice. Exemplary PET/CT images of ^64^Cu-labeled (**A**) and ^68^Ga-labeled (**B**) ligands in MDA-MB-468 tumor-bearing mice at 1 to 2 h after intravenous injection of the indicated radiotracers. 6–9 MBq/animal (0.3–0.45 nmol/animal) were injected. All images are scaled to a maximum SUV of 1.5. **C/D/H/I**) Time-activity curves (SUVmean, decay-corrected, as a function of time up to 2 h) for the blood content of the heart (**C/H**) and of the tumor (**D/I**) of ^64^Cu-labeled (**C/D**) and ^68^Ga-labeled ligands (**H/I**), respectively, obtained from quantitative analysis of PET images. Time-resolved SUVmean ratios up to 2 h p.i., including tumor-to-muscle (**E/J**) and tumor-to-heart (**F/K**) of ^64^Cu-labeled (**E/F**) and ^68^Ga-labeled ligands (**J/K**), respectively. **G/L**) Time-resolved body retention in % of the initial dose up to 2 h p.i. Data points in **C–L** represent mean values (±SD) of groups of three tumor-bearing mice, except [**^68^Ga]­Ga-NECT-346** which represents only two mice.

**[** ^**68**^ **Ga** **]­Ga-NECT-224** and **[** ^**68**^ **Ga** **]­Ga-NECT-346** were also characterized using the MDA-MB-468 tumor model. Here, while tumor uptake was higher for [^**68**^ **Ga]­Ga-NECT-224** compared to [^**68**^ **Ga]­Ga-NECT-346** (Figure 6I), the T/M and T/H ratios tended to be more favorable for [^**68**^ **Ga]­Ga-NECT-346** compared to [^**68**^ **Ga]­Ga-NECT-224** (Figure 6J,K).

In addition to the MDA-MB-468 tumor model, [^**64**^ **Cu]­Cu-NECT-224** and [^**64**^ **Cu]­Cu-NECT-346** were also characterized using the Nectin-4-negative MDA-MB-231 tumor model (Figures S6 and S7 and Table S4). Tumor tissue was barely detectable in the PET/CT images, which was also reflected by the comparably low maximum SUVmean values of ≈0.5 and T/M (2.3 and 3.3 for [^**64**^ **Cu]­Cu-NECT-224** and [^**64**^ **Cu]­Cu-NECT-346**, respectively) and T/H ratios (1.1 and 1.6 for [^**64**^ **Cu]­Cu-NECT-224** and [^**64**^ **Cu]­Cu-NECT-346**, respectively) at 1–2 h p.i.

### First-in-Human Application of [^64^Cu]­Cu-NECT-224

Due to the consistently better in vivo performance of the ^64^Cu-labeled Nectin-4 ligands compared to their ^68^Ga-labeled analogues and our previous experience with [^**68**^ **Ga]­Ga-NECT-224**, we initiated a first-in-human application of [^**64**^ **Cu]­Cu-NECT-224**. Of course, our preclinical data herein indicate that the novel radioligands, in particular [^**64**^ **Cu]­Cu-NECT-334** and [^**64**^ **Cu]­Cu-NECT-346**, show superior imaging properties than [^**64**^ **Cu]­Cu-NECT-224**, but the application of [^**64**^ **Cu]­Cu-NECT-224** was more straightforward, and one aim for the first-in-human application was also to demonstrate the general suitability of a ^64^Cu-labeled Nectin-4 ligand for PET/CT imaging in humans.

A 68-year-old man with recurrent metastatic urothelial carcinoma of the urinary bladder and multiple prior therapies, who was initially diagnosed 4 years ago, underwent a **[** ^**64**^ **Cu** **]­Cu-NECT-224** PET/CT as part of an individual diagnostic procedure, after providing informed consent and within an individualized diagnostic concept, prior to radiotherapy for a symptomatic femoral and pelvic bone metastasis. The prior therapies included transurethral resection of the bladder tumor, multiple reresections of local tumor recurrences, chemotherapy, immunotherapy, and radiotherapy to both the primary tumor region and bone metastases. At the time point of the scan, the patient had received no systemic treatment within the preceding 3 years. Figure 7 shows the PET scan as maximum-intensity projection (MIP) images at 67 min and 16.5 h after intravenous injection of [^**64**^ **Cu]­Cu-NECT-224**. Apart from physiological distribution of the radiotracer, including renal clearance, several Nectin-4-positive bone metastases, especially in the left hemipelvis, and a left external iliac lymph node metastasis, are visible. The latter had an SUVmax of 10.8 (SUVpeak: 5.4) at 67 min p.i., which decreased to 3.9 (SUVpeak: 1.3) at 16.5 h p.i. (Figure 7C/D). As another example, the most prominent and symptomatic iliac bone metastasis with a soft-tissue component demonstrated a decrease in SUVmax from 10.2 (SUVpeak: 5.0) at 67 min p.i. to 3.9 (SUVpeak: 1.3) at 16.5 h p.i. (Figure 7E/F).

**Figure 7:**
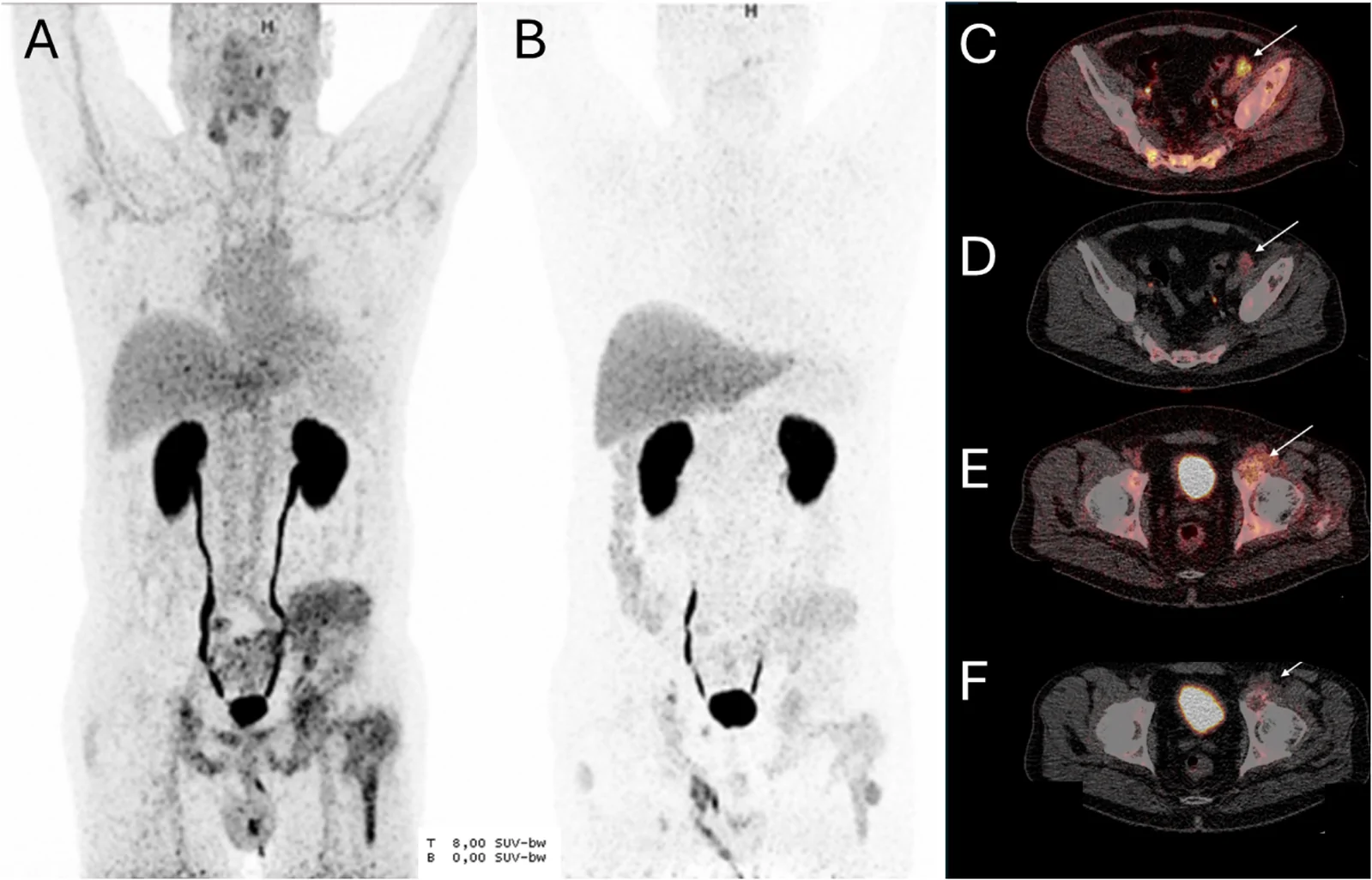
First-in-human application of **[^64^Cu]­Cu-NECT-224**. **A**/**B**) Maximum-intensity projection (MIP) scaled to a maximum standardized uptake value (SUVmax) of 8, acquired in the anterior view, demonstrating the physiological distribution of [**^64^Cu]­Cu-NECT-224** alongside multiple tumor manifestations. Imaging was performed at 67 min (**A**) and 16.5 h (**B**) after intravenous injection of 251 MBq of [**^64^Cu]­Cu-NECT-224**. **C/D**) Representative axial fused PET/CT images (scaled to an SUVmax of 8) are shown with a left external iliac lymph node metastasis indicated by a white arrow (**C** at 67 min and **D** at 16.5 h p.i.). **E/F**) Representative axial fused PET/CT images (scaled to an SUVmax of 8) are shown with a bone metastasis with a soft-tissue component indicated by a white arrow (**E** at 67 min and **F** at 16.5 h p.i.).

In terms of activity uptake in normal tissues, the initially high uptake in the renal pelves (SUVmax ≈ 200) had vanished in the 16.5 h p.i. images, while the moderate uptake in the renal parenchyma remained stable (right, SUVmean: 10.7 vs 9.3 for 67 min and 16.5 h, respectively, Figure S12). Furthermore, a decrease in activity over time was observed for the liver (SUVmax 3.5 vs 2.6), the spleen (SUVmax 1.9 vs 1.0), the vascular blood pool (SUVmax 4.5 vs 0.5), and the salivary gland (SUVmax 3.1 vs 0.8, right submandibular gland).

## Discussion

Nectin-4 has been proven as a valid target for antibody- and peptide-drug conjugates with **enfortumab vedotin** being clinically approved by the EMA and the FDA, in combination with **pembrolizumab** (an antibody targeting PD-1), as first-line treatment in patients with locally advanced or metastatic urothelial carcinoma.^11^ ,^12^ While the assessment of the patient’s Nectin-4 status was not required in any registration trial,^44,45^ there is a clinical demand for quantifying the Nectin-4 status in patients prior to a potential targeted chemotherapy as e.g., the Nectin-4 level differs among molecular subtypes of urothelial cancer^46^ and can also be heterogeneous between primary and metastatic lesions.^47^ This sets the basis for the development of Nectin-4-directed radioligands, with antibody- and peptide-based radioligands being pursued in recent years. Just recently, Mishra et al. impressively demonstrated in preclinical studies that PET-measured target engagement derived from pre- and post-treatment SUV values with a ^68^Ga-labeled bicyclic peptide ([^**68**^ **Ga]­Ga-AJ647**) targeting Nectin-4 predicted the therapeutic outcomes of **enfortumab vedotin** better than the drug dose or the pretreatment SUV values alone.^48^ This might further stimulate the exploration of diagnostic radioligands toward Nectin-4 in the context of personalized medicine.

Previously, we translated the bicycle toxin conjugate **BT8009** into ^68^Ga- and ^64^Cu-labeled ligands for noninvasive imaging with PET.^39^ Among a series of ligands, **NECT-224**, in particular as the ^64^Cu-labeled version, demonstrated the most favorable in vivo performance as assessed in a urothelial carcinoma xenograft model (HT-1376). Although tumor uptake and T/M and T/H ratios were sufficient for imaging purposes - also highlighted by a first-in-human application of **[** ^**68**^ **Ga** **]­Ga-NECT-224** - our data on the binding kinetics to Nectin-4 gave an incentive to further structurally optimize the bicyclic peptides to improve their in vivo performance. In particular, this becomes of interest, when tumor entities with a lower abundance of Nectin-4 compared to that of urothelial carcinoma are considered.

Herein, we introduced three novel Nectin-4 ligands that are derived from **NECT-224** but bear additional linker moieties within the bicyclic scaffold and the chelating unit NODAGA. This design was primarily motivated by our findings that the N-terminal moiety can have a significant impact on the binding kinetics of Nectin-4. Second, hydrophilic linkers such as PEG or poly-Sar might improve tumor-to-background ratios due to more rapid washout from normal tissues.^49^ Regarding the choice of linker moieties, we chose Sar_10_ as realized in **NECT-346**, which also separates the MMAE toxin unit in **BT8009** from the bicyclic peptide.^22^ A Sar_10_ linker was also exploited previously for radiolabeled bicyclic peptides directed to MMP14.^50^ In addition, PEG-based linkers of shorter (PEG_2_, **NECT-330**) and similar length (PEG_8_, **NECT-334**) compared to Sar_10_ were evaluated. During our work, a similar ligand design was exploited by Wan et al.^36^ (**NOTA-PEG**_**2/4/6/12/24**_ **-BP**) and Sun et al.^33^ (Sar_10_, PEG_5_, and PEG_8_ in **FZ-NR-1, FZ-NR-2, and FZ-NR-3**, respectively, all with DOTA). In contrast to our ligands, which bear methoxinine at position 4, these bicyclic peptides bear the original methionine from **BT8009**. We previously highlighted that this amino acid substitution led not only to higher radiochemical purity upon labeling with ^64^Cu or ^68^Ga but also to improved tumor uptake in the case of ^64^Cu labeling.^39^

All novel Nectin-4 ligands showed more favorable binding kinetics to hNectin-4 by means of lower *k*_off_ values compared to **NECT-224**, indicating that the direct attachment of the bulky chelating unit to the N-terminus is somewhat unfavorable. In line with these findings, **NECT-416,** bearing a free N-terminus, exhibited also a lower *k*_off_ value compared to **NECT-224**. However, rather than the steric demand of the NODAGA moiety, it seems that the charge of the N-terminal substituent is crucial for binding to Nectin-4, as the ^nat^Cu and ^nat^Ga complexes of **NECT-224** showed lower *k*_off_ values compared to their metal-free analogs. In addition to characterizing binding kinetics to hNectin-4, **NECT-224** as well as the novel linker-bearing ligands were characterized toward mNectin-4 and hNectin-1, -2, and -3. In line with data for **BT8009**,^23^ our Nectin-4 ligands were selective for hNectin-4, with comparable binding kinetics toward human and mouse Nectin-4, while no binding to hNectin-1, -2, and -3 was observed up to 200 nM.

Encouraged by these findings, we envisaged to characterize the novel ligands in our previously established UC cell and tumor models^39^ and expanded these studies also to TNBC models. TNBC represents a tumor entity for which targeted theranostic approaches are largely missing with Nectin-4 being a promising molecular target.^51^ In this context, **BT8009** is currently evaluated in a Phase II study in patients with breast cancer, including TNBC (NCT06840483). While HT-1376 and 5637 cells, as well as derived tumor xenografts, were characterized previously by us for Nectin-4 abundance,^39^ we herein characterized the MDA-MB-468 and MDA-MB-231 TNBC cell lines and their respective tumor xenografts. MDA-MB-468 was chosen as the Nectin-4-positive cell line and MDA-MB-231 as the Nectin-4-negative one, which is in accordance with previous reports.^30,31,41^ The Nectin-4 abundance was confirmed herein by Western blot analysis and its membranous location by immunofluorescence staining. Furthermore, quantification of the Nectin-4 level was performed with an ELISA. Accordingly, MDA-MB-468 cells exhibited a similar protein level to that of HT-1376 cells, while no Nectin-4 could be detected by either method for the MDA-MB-231 cells. MDA-MB-468 tumor xenografts maintained a comparable Nectin-4 level to that of their monolayer culture, and again no Nectin-4 could be detected in MDA-MB-231 tumor xenografts. Therefore, the latter tumor model can be considered as a truly Nectin-4-negative xenograft model, which is distinct from the data for the UC cell line 5637. For 5637, the monolayer culture lacked a measurable Nectin-4 level, but a significant amount of this protein was present in tumor xenografts derived thereof.^39^ In fact, the Nectin-4 level in 5637 tumor xenografts was comparable to that of the MDA-MB-468 tumor xenografts. The Nectin-4 positivity of 5637 tumors was also previously demonstrated by Wan et al. with immunohistochemical staining, albeit the authors, unlike us, were also able to detect Nectin-4 at the cellular level by immunofluorescence staining.^36^

Having established Nectin-4-positive and -negative TNBC models in addition to UC models, we directed our interest to the phenomenon of Nectin-4 shedding from the cell surface. Fabre-Lafay et al. discovered previously that Nectin-4 can serve as a histological and serological marker for breast cancer.^41^ The same group reported that the detected sNectin-4 originated from ectodomain shedding catalyzed by the metalloprotease ADAM17, which is also abundant on breast cancer cells.^42^ A subsequent study by Buchanan et al. highlighted that ectodomain shedding of Nectin-4 occurs also in ovarian cancer and is mediated by ADAM10 and ADAM17.^43^ Inspired by these reports, we sought to assess this phenomenon in our UC and TNBC models and to characterize the potential impact of Nectin-4 shedding on radioligand binding. At first, the sNectin-4 level could be detected in the supernatant of HT-1376 cells. By quantification of sNectin-4 in the urine of mice subcutaneously injected with HT-1376 and MDA-MB-468 cells, an increase in the sNectin-4/creatinine value could be detected over time. For the HT-1376 tumor model, sNectin-4/creatinine correlated well with the tumor volume quantified by PET/CT imaging with **[** ^**64**^ **Cu** **]­Cu-NECT-224**. Of note, correlations with the tumor volume obtained by caliper or CT measurements were significantly worse because of the presence of a necrotic core in the HT-1376 tumors. In this context, a correlation of urine Nectin-4 and the tumor H-score of Nectin-4 was previously found by Miyake et al. in patients with bladder cancer.^52^

To explore the enzymes involved in Nectin-4 shedding, we studied the time- and concentration-dependent occurrence of sNectin-4 in the supernatant of HT-1376 cells in the presence of the two commercially available ADAM10/17 inhibitors **JG26** ^53^ and **INCB3619**.^54^ A dose-dependent reduction of the sNectin-4 level was detectable by both inhibitors; however, **INCB3619** appeared to be 10 times more potent than **JG26** (relative reduction of sNectin-4 to ≤ 50% at 100 and 1000 nM for **INCB3619** and **JG26**, respectively). Considering the known inhibitory potencies of both compounds toward ADAM10 (IC_50_ values of 150 and 22 nM for **JG26** and **INCB3619**, respectively) and ADAM17 (IC_50_ values of 1.9 and 14 nM for **JG26** and **INCB3619**, respectively), our results indicate that Nectin-4 shedding from the HT-1376 cells is primarily driven by ADAM10. Quantification with ELISA confirmed the presence of both metalloproteases in all four UC and TNBC cell lines and tumor xenografts derived thereof. However, apparent protein levels of ADAM10 were considerably lower compared to levels of ADAM17, which might question our results with the above-mentioned protease inhibitors. In this context, after having performed the ADAM10 ELISA, we noted that ADAM10 can autocatalytically and rapidly degrade itself upon cell lysis, which affects its mature but not its pro form.^55^ This might explain the results for the ADAM10 ELISA herein and suggests an adjusted preparation of cell lysate samples for future studies.

Generally, the shedding of Nectin-4 from the cell surface is detrimental to any targeted strategy, as a lower binding capacity or release of the initially bound target molecule can occur. Furthermore, released Nectin-4, i.e., sNectin-4, in the blood circulation might compete for binding of the radioligand to tumor-associated Nectin-4. However, to be relevant for a targeted strategy, the fraction of sNectin-4 over time compared with the cell-bound Nectin-4 is crucial. Herein, we were able to quantify for HT-1376 cells that <2% of cell-bound Nectin-4 is released as sNectin-4 per hour. Accordingly, the binding of [^**64**^ **Cu]­Cu-NECT-224** in the absence and presence of **INCB3619** was not different. In vivo blocking of Nectin-4 shedding in HT-1376 tumor-bearing mice was attempted upon preadministration of **INCB3619** (1.4 mg/kg at 17 and 1 h before radiotracer injection). However, this dosing regimen did not affect the sNectin-4 level in the urine or the tumor uptake of [^**64**^ **Cu]­Cu-NECT-224**, which in turn limits conclusions about the relevance of Nectin-4 shedding on the tumor targeting by radioligands. In a previous study on HER-2 extracellular domain (ECD) cleavage, which is also mediated by ADAM proteases, **INCB3619** was continuously given at 30 mg/kg/day over 6 days. This dosing regimen efficiently reduced serum ECD levels and enhanced the antitumor effect of trastuzumab in a preclinical tumor model.^54^ Considering that an analogue of **INCB3619** reduced serum ECD levels even at 3 mg/kg/day and considering the commercial availability of **INCB3619**,^54^ we hypothesized that a double preadministration of 1.4 mg/kg before radiotracer injection would be sufficient to block ADAM10/17 activity and thus to reduce sNectin-4 in the urine. But obviously, our dosing regimen was too low or too short to efficiently block the proteolytic activity of ADAM10 and 17 and/or to measurably affect the sNectin-4 level. Therefore, further studies are needed to characterize the influence of the shedding phenomenon on tumor uptake by Nectin-4-directed radioligands.

With the detailed characterization data for the UC and TNBC cell lines and tumor xenografts in hand, we performed comparative cell-binding and internalization studies with the novel radioligands and our previously developed radioligands [^**64**^ **Cu]­Cu-NECT-224** and [^**68**^ **Ga]­Ga-NECT-224**. In line with the SPR data for the novel ligands, the ^64^Cu-labeled analogues exhibited potent Nectin-4-specific cell binding accompanied by a high extent of putative internalization. It is worth noting that the acid wash-resistant fractions were higher toward HT-1376 compared to MDA-MB-468 cells for the same radioligand. This might indicate a cell-type-specific pharmacological effect upon radioligand binding to Nectin-4. In line with this finding, the *B*_max_ values were considerably higher toward HT-1376 compared to MDA-MB-468, and, simultaneously, lower apparent *K*_d_ values were determined using the latter cell line. We recently characterized the temperature- and time-dependent binding of [^**64**^ **Cu]­Cu-NECT-224** to HT-1376 cells and proposed that pronounced internalization occurs, which depend on Nectin-4, but the protein itself seems to remain at the cell surface.^39^ Therefore, higher *B*_max_ values than expected were found based on the amount of Nectin-4 as determined by ELISA. This apparent shuttling of the radioligand inside the ells is even more pronounced for the novel linker-bearing radioligands compared to [^**64**^ **Cu]­Cu-NECT-224**. Even though higher *B*_max_ values were also obtained toward MDA-MB-468, the difference from the actual protein amount is lower. The underlying reasons need to be assessed in subsequent studies.

Comparative PET/CT imaging studies of the novel ^64^Cu- and ^68^Ga-labeled ligands revealed a clearly improved in vivo performance in both UC and TNBC tumor models compared with our previously developed radioligands [^**64**^ **Cu]­Cu-NECT-224** and [^**68**^ **Ga]­Ga-NECT-224**. Although the shape and height of the tumor TAC are usually not affected by the introduced linker units, a significant enhancement of the T/M and T/H ratios was consistently obtained, which primarily originated from a faster washout from the normal tissues, exemplified by a reduced elimination half-life in the blood pool. Among the novel ligands, ^64^Cu- and ^68^Ga-labeled **NECT-334** and **NECT-346**, bearing PEG_8_ and Sar_10_ linker units, respectively, slightly outperformed radiolabeled **NECT-330** with a short PEG_2_ linker. It is worth noting that higher SUVmean values in the 5637 and MDA-MB-468 tumors were obtained by the novel radioligands compared to radiolabeled **NECT-224,** but not in the HT-1376 tumors. Furthermore, the ^64^Cu-labeled ligands showed superior in vivo performance compared to their ^68^Ga-labeled analogues, a phenomenon that was also highlighted in our previous study.^39^ A detailed reason for this finding can still not be given. Previously, [^**68**^ **Ga]­Ga-FZ-NR-1** was preclinically evaluated in MDA-MB-468 and MDA-MB-231 cell and tumor models. PET/CT imaging in MDA-MB-468-bearing mice revealed a T/M ratio of 4.5 at 60 min p.i., which is similar to the ratio of [^**68**^ **Ga]­Ga-NECT-346** in the same tumor model and at the same time point, but lower than the ratio obtained with [^**64**^ **Cu]­Cu-NECT-346**.

In addition to Nectin-4-directed radioligands based on bicyclic peptides, several immunoPET and immunoSPECT approaches were published previously.^27–31^ Among others, Weibo Cai and colleagues reported on different immunoPET probes based on ^64^Cu- or ^89^Zr-labeling of appropriately modified **enfortumab vedotin** ([^**64**^ **Cu]­Cu-NOTA-Padcev** ^27^ and [^**89**^ **Zr]­Zr-DFO-Padcev**,^28^ respectively) or a bivalent antibody fragment derived from this ADC ([^**64**^ **Cu]­Cu-NOTA-EV-F­(ab’)**_**2**_^29^). Due to the different pharmacokinetic behavior of antibodies or antibody fragments, the activity retention in normal organs, including the blood circulation, is higher compared to small radiolabeled peptides, which is accompanied by a shift in the tumor TAC to later time points. For [^**64**^ **Cu]­Cu-NOTA-EV-F­(ab’)**_**2**_, maximum tumor uptake in an MDA-MB-468 model was observed at 4 h p.i., and maximum T/H (starting at 12 h p.i.) and T/M (starting at 4 h p.i.) ratios of 3 and 10, respectively.^29^ In contrast, [^**89**^ **Zr]­Zr-DFO-Padcev** showed maximum tumor uptake at 48 h p.i. in the same tumor model and maximum T/H and T/M ratios of approximately 4 and 20, respectively, at the same time point.^28^ Considering the results for [^**64**^ **Cu]­Cu-NECT-346** in the MDA-MB-468 tumor model herein, with the most favorable T/H and T/M ratios of 6.2 and 10.0 being reached at 1–2 h p.i., [^**64**^ **Cu]­Cu-NECT-346** enables earlier imaging acquisition, which is favorable for clinical applications, and provides the advantage of even higher T/H ratios compared to the aforementioned immunoPET approaches.

In addition to the desired uptake in the tumor tissue, a prominent activity uptake was seen for all radioligands in the kidneys and bladder, which aligns with the renal excretion route of the radioligands. However, a considerable activity uptake was also noted in the salivary glands (e.g., for [^**64**^ **Cu]­Cu-NECT-224**, a maximum SUVmean of 2.5, Figure S13), which was even more pronounced for the novel radioligands compared to radiolabeled **NECT-224**. In line with this, activity uptake in the salivary glands in mice was also noted for other Nectin-4-directed radioligands^36^ and even in humans.^56^ While it is often stated that Nectin-4 is largely absent in adult tissues, Nectin-4 was actually detected in a range of normal tissues, including the bladder, breast, stomach, salivary glands, and skin.^9^ To shed further light on this finding, we sought to quantify the level of mNectin-4 in the kidneys, salivary glands, and liver of NMRI-nu/nu mice. According to the ELISA data, high mNectin-4 levels were detected in these normal tissues, with the mNectin-4 level in the kidneys even exceeding that of the hNectin-4 level in the tumor xenograft models. Consequently, activity uptake and prolonged retention in the kidneys and salivary glands might also result from the physiological abundance of this protein in these tissues. However, it is worth noting that the activity retention in the salivary glands is shorter compared to the tumor tissue, which, however, might not be reasoned by different binding kinetics of the ligands toward mouse and human Nectin-4 as shown herein.

Besides ^64^Cu and ^68^Ga, we performed pilot experiments with ^61^Cu-labeled **NECT-224** and **NECT-346** using the HT-1376 tumor model. ^61^Cu represents another copper radioisotope with suitable nuclear physical properties for PET imaging.^57^ In this context, ^61^Cu-labeled radioligands have also entered clinical interest, with [^**61**^ **Cu]­Cu-NODAGA-LM3**, an SST_2_ antagonist,^57^ being currently investigated in a prospective clinical phase I/II study (NCT06455358), and recently, imaging results of the first patient from this clinical trial were published.^58^ Considering the fast tumor uptake and rapid blood clearance of the presented Nectin-4 ligands, the short half-life of ^61^Cu compared to ^64^Cu (3.4 vs 12.7 h) fits more properly to the biological half-life of the targeting molecules. Furthermore, due to the shorter half-life and more favorable nuclear properties of ^61^Cu compared to ^64^Cu (e.g., no fraction of β^–^ decay for ^61^Cu), the radiation exposure of healthy organs upon injection of ^61^Cu-labeled ligands might be lower compared to their ^64^Cu-labeled counterparts.^59,60^ Of course, from a chemical point of view, the ^61^Cu-labeled peptides are identical to their ^64^Cu-labeled analogs and thus the biodistribution of both radioligands should be equal. While this could be shown for [^**61**^ **Cu]­Cu-NECT-346**, higher T/M and T/H ratios were obtained for [^**61**^ **Cu]­Cu-NECT-224** as expected based on the data for its ^64^Cu-labeled analog, which rendered the in vivo performance of [^**61**^ **Cu]­Cu-NECT-224** equal to [^**61**^ **Cu]­Cu-NECT-346**. Although a reason for this surprising finding cannot be given at this stage, our data at least indicate the suitability of both ligands for PET/CT imaging upon labeling with ^61^Cu.

Encouraged by the consistently better in vivo performance of the ^64^Cu-labeled ligands compared with their ^68^Ga-labeled pendants, we envisaged the first-in-human application of [^**64**^ **Cu]­Cu-NECT-224**. A clear detection of the Nectin-4–positive lesions was possible, and the physiological distribution of [^**64**^ **Cu]­Cu-NECT-224** appeared to be similar to that of [^**68**^ **Ga]­Ga-NECT-224** or [^**68**^ **Ga]­Ga–N188** according to our previous experiences.^39,61^ In this context, activity uptake in the salivary glands occurs, which was also noted for [^**68**^ **Ga]­Ga-FZ-NR-1** previously,^56,62^ and aligns with our data herein on the Nectin-4 abundance in salivary glands. However, a decreasing activity level was observed in the salivary glands over time, while the initial activity uptake in the renal parenchyma remained constant at 16.5 h p.i. Previously, we showed by ex vivo metabolite analysis upon injection of **[** ^**64**^ **Cu** **]­Cu-NECT-224** in mice that a distinct radiometabolite was formed in the kidneys. Current studies by our group are focused on the structural elucidation of this radiometabolite and its retention behavior in the kidneys. Apart from activity uptake in normal organs, the additional image acquisition at a later time point (16.5 h) revealed that some lesions could still be detected, but there was a general trend to lower SUVmax values compared to the early imaging time point (67 min). This in turn indicates that our current generation of Nectin-4 ligands still needs to be improved to enable also radionuclide therapy besides pure imaging purposes. Although a direct comparison regarding the performance of [^**64**^ **Cu]­Cu-NECT-224** and [^**68**^ **Ga]­Ga-NECT-224** is not possible at the moment, the ^64^Cu-labeled analogue offers centralized production and subsequent distribution, which might further support the characterization of Nectin-4 as a molecular target in cancer patients. The results of the first-in-human application of [^**64**^ **Cu]­Cu-NECT-224** are also encouraging for the clinical translation of the novel radioligands presented herein, in particular [^**64**^ **Cu]­Cu-NECT-334** and [^**64**^ **Cu]­Cu-NECT-346**, which could provide even better tumor-to-background contrast in humans, as observed preclinically.

## Conclusion

This study demonstrates that **NECT-330**, **NECT-334**, and **NECT-346**, which are structurally advanced from **NECT-224**, are highly potent and selective ligands for Nectin-4. Owing to the complexation properties of the versatile chelator (*R*)-NODAGA, a detailed radiopharmacological characterization in UC and TNBC models upon labeling with ^61^Cu, ^64^Cu, and ^68^Ga was performed, which revealed an overall superior in vivo performance compared to previous [^**64**^ **Cu]­Cu-NECT-224** and [^**68**^ **Ga]­Ga-NECT-224** with the best T/M and T/H ratios being provided by [^**64**^ **Cu]­Cu-NECT-334** and [^**64**^ **Cu]­Cu-NECT-346**, which warrants their further clinical translation. This is further encouraged by the first-in-human application of [^**64**^ **Cu**]­Cu-NECT-224 presented herein, showing the general suitability for Nectin-4-directed PET/CT imaging in patients with ^64^Cu-labeled bicyclic peptides. Accordingly, we assume that the novel ligands also enable the exploration of noninvasive imaging of tumor-associated Nectin-4 in tumor entities with even lower abundance of this protein compared to UC and TNBC. While we were able to demonstrate that the shedding of Nectin-4 does not affect the overall binding of radioligands in vitro, a definitive statement regarding the targeting of tumor-associated Nectin-4 by radioligands cannot be given. However, according to the in vitro data, we assume no detrimental effect of the shedding phenomenon on radioligand binding to tumor-associated Nectin-4. In ongoing studies, we seek to further structurally modify the bicyclic scaffold originally derived from **BT8009**, as the binding kinetics, in particular the *k*_off_ values, still need to be improved to achieve longer tumor retention, which, in turn, would also pave the way toward a Nectin-4-directed radioligand therapy.

## Materials and Methods

### Chemistry and Radiochemistry

All chemicals were purchased from BLD Pharmatech, IRIS Biotech, ABCR, Thermo Fisher Scientific and Carbolution. The detailed synthesis protocol of all peptides are provided in the Supporting Information. For radiolabeling with in-house produced^61^Cu and ^64^Cu,^63–66^ [^61^Cu]­CuCl_2_ or [^64^Cu]­CuCl_2_ each in 0.01 M HCl was diluted with 0.1 M NH_4_OAc (pH 5.6) to a final activity concentration of 1 MBq/μL. To the peptides (1–6 μL of 2 mM DMSO stock, 2–12 nmol), 50–300 MBq of the prepared [^61^Cu]­CuCl_2_ or [^64^Cu]­CuCl_2_ solutions were added. The labeling reaction was carried out at 60 °C for 20 min and the radiochemical yield was assessed by radio-HPLC analysis. Radiolabeling with ^68^Ga was performed using [^68^Ga]­GaCl_3_ eluted with 0.1 M HCl from a ^68^Ge/^68^Ga generator (Eckert & Ziegler). Peptides (2 μL of 2 mM DMSO stock, 4 nmol) were labeled with [^68^Ga]­GaCl_3_ (75 MBq, 250 μL) in 1 M sodium acetate buffer (pH 4.5). The labeling reaction was carried out at 90 °C for 10 min and the radiochemical yield was assessed by radio-HPLC analysis. LogD_7.4_ values and stability in human plasma were assessed as previously described.^39^

### Surface Plasmon Resonance Spectroscopy

SPR analysis of binding kinetics to human Nectin-4 (R&D Systems, Cat. No. 2659-N4) and mouse Nectin-4 (Acro Biosystems, Cat. No. NE4-M52H3) was performed as previously described for human Nectin-4.^39^ In brief, binding analysis was performed by consecutive injection of five increasing concentrations of the bicyclic peptide (120 s contact time for each concentration), followed by 600 s dissociation time, and analysis on both flow cells (FCs). Regeneration was performed by sequential injection of 10 mM NaOAc (pH 5.5) for 60 s, washing the needle with HBS-P+, injection of 10 mM NaOAc (pH 5.5) for 60 s, a 600 s wait, four injections of HBS-P+ (60 s), and a final 600 s waiting for stabilization. Blank runs using only buffer (HBS-P+) instead of sample were carried out to obtain double-referenced sensorgrams. Unless otherwise stated, binding analyses were performed in a concentration range between 0.16–100 nM or 1.6–1000 nM (fivefold dilution series) and analyzed in triplicate. The data were analyzed using Biacore Evaluation Software 3.2.1. Each sensorgram was reference-subtracted (FC­(active) – FC­(reference)) and blank-corrected. Data were fitted to a 1:1 binding model.

For assessing isoform specificity, the human isoforms Nectin-1, -2, and -3 (Acro Biosystems, Cat. No. PV1–H5223, PV2–H52E2, PV3–H52E4), and -4 were immobilized on a CM5 chip at levels of 1394 RU, 849 RU, 1835 RU, and 1319 RU on flow cells 1, 2, 3, and 4, respectively, using the protocol described above. Binding analyses were performed by injection of the ligand at a concentration of 200 nM over all four flow cells and analysis of the binding signal 60 s after injection of the peptide, compared to the baseline signal before injection. Of note, this was performed because solvent-based signal shifts during analyte injection did not allow for proper evaluation, and no reference cell was available in this setup. Hence, we decided to evaluate the binding signal in the dissociation phase, which is not influenced by this shift and also allows us to discriminate binders with significantly lower *k*_off_ values from comparably weak binders with high *k*_off_ values.

### Cell Culture

HT-1376 cells were cultured in DMEM supplemented with GlutaMAX, 10% FBS, and 1% penicillin/streptomycin, whereas 5637 cells were cultured in RPMI supplemented with GlutaMAX, 10% FBS, and, 1% penicillin/streptomycin. Both cell lines were cultured in 5% CO_2_ and at a relative humidity of 95% at 37 °C. MDA-MB-231 and MDA-MB-468 cells were cultured in L15 supplemented with 10% FBS and 1% penicillin/streptomycin, without additional CO_2_ at 37 °C. All radioligand binding assays were performed using DMEM supplemented with 10% FBS and 1% penicillin/streptomycin.

### Enzyme-Linked Immunosorbent Assay (ELISA)

The human Nectin-4 ELISA kit—Quantikine (#DNEC40) from R&D Systems, the mouse Nectin-4 ELISA kit (#ELM-NECTIN4–1) from RayBiotech, the human ADAM10 ELISA kit from Antibodies Online (#ABIN6953443), and the human TACE/ADAM17 ELISA kit (#EHADAM17) from ThermoFisher were used. All ELISAs were performed according to the manufacturer instructions.

Cell supernatant samples were collected from the culture vessel at the indicated time points. They were centrifuged for 5 min at 1000 × g, and the supernatant was transferred to a new tube and used for the assay. For cell lysis samples, the cell lines were cultured to a confluency of ∼90% and then briefly washed three times with cold PBS. Lysis Buffer 2 (R&D #895347) was added to the cells, and after a 10-min incubation on ice, the lysed cell layer was scraped from the surface using a cell scraper. The lysate was centrifuged at 14,000 × *g* for 15 min, and only the supernatant was used for further analysis.

To prepare the tumor lysates, tumor samples of ≈4 × 4 mm were collected after the animal was sacrificed, avoiding the necrotic core. The tissue was lysed in ∼300 μL of lysis buffer 2 (R&D #895347) using the GentleMACS device (Miltenyi Biotec). To collect urine samples, the mice were separated in an empty cage either directly after PET imaging or at regular intervals of tumor growth. The urine was immediately collected and centrifuged at 10,000 × *g* for 5 min at 4 °C. Only the clear supernatant was used for any analysis.

### Determination of Creatinine Levels

The urine samples used for ELISA analysis were also analyzed for their creatinine concentrations using the Parameter Creatinine Assay (#KGE005) from R&D Systems. The assay was performed according to the manufacturer’s instructions. All measurements of soluble Nectin-4 in urine samples were normalized to creatinine content and are given in fmol of Nectin-4 per mg of creatinine.

### Cell Binding, Internalization, and Saturation Binding of Radioligands

Cell binding and internalization experiments for the ^64^Cu- and ^68^Ga-labeled ligands using HT-1376, 5637, MDA-MB-468, and MDA-MB-231 cells were performed as described previously.^39^ In brief, cells were seeded in a 48-well plate 4 to 5 days prior to the experiment so that confluence reached approximately 95 to 100% on the day of the assay. Immediately before the radioligand binding experiments, the cells were washed with fresh medium once. Total binding of the radioligands (10 nM) as well as the nonspecific binding (in the presence of 1 μM **NECT-208** or **NECT-214**, previously denoted as compounds **1e** and **3a**,^39^ respectively) were determined in order to calculate the specific binding. If not indicated otherwise, only specific binding data are presented. The cells were incubated with the radioligand dilution for 1 h at 37 °C and 300 rpm on a shaker. The radioligand was removed and cells were washed twice with ice-cold PBS+. For determining internalization, a wash step with cold glycine buffer (50 mM, pH 2.8, 5 min) was included. For selected radioligands, saturation binding analyses were performed using 10 different radioligand concentrations (0.321–160 or 0.156–80 nM).

To assess the influence of Nectin-4 shedding on radioligand binding, cell binding of [^**64**^ **Cu]­Cu-NECT-224** at 10 nM was also performed in the presence of 100 nM ADAM10/17 inhibitor **INCB3619** (MCE, no. HY-12636). For this assay, the washing step was performed using medium containing 100 nM **INCB3619**. After the washing step, cells were incubated with the inhibitor for approximately 15 min before the assay was started.

### Immunoblot

An SDS-PAGE gel was loaded with 25 μg cell lysate of MDA-MB-468 and MDA-MB-231 per lane and subsequently blotted to a PVDF membrane (Cytiva #RPN3032D). The anti-Nectin-4 antibody (ThermoFisher #PA5–47365) was diluted 1:200 in TBS-T + 3% BSA, and the membrane was incubated with the antibody dilution overnight at 4 °C. The membrane was briefly washed three times with TBS-T and probed with an antigoat IgG HRP-conjugate (Sigma #A5420) diluted 1:20000 in TBS-T + 3% BSA for 2 h at room temperature. The washing procedure was repeated, and the bands were visualized with SuperSignal West Femto Maximum Sensitivity Substrate (ThermoFisher #34095) at a Celvin S Chemiluminescence Imager (Biostep).

### Small-Animal PET

Small animal PET/CT was performed with a nanoPET/CT scanner (Mediso Medical Imaging Systems, Budapest, Hungary). The PET experiments with the radiotracers were conducted when the tumors reached a volume of ∼200 mm^3^. The radiotracers were diluted in 0.9% NaCl (^61^Cu- and ^64^Cu-labeled ligands) or PBS (^68^Ga-labeled ligands), and 200 μL containing 6–9 MBq (0.3–0.45 nmol) were injected into the lateral tail vein of the mice. A continuous PET scan over 2 h to record coincidences was performed starting at the time of injection. For anatomical reference and attenuation correction, a CT scan was performed. Image reconstruction, time framing, and tissue delineation were performed as described previously.^67^ Preclinical PET images were postprocessed and analyzed using Rover version 3.0.77h (ABX, Radeberg, Germany). Details on the extraction of tissue-specific uptake values from preclinical PET images (region-averaged standardized uptake values, SUVmean) are provided in Figure S14. Additionally, static uptake values were extracted and compared for the 1–2 h time frame, which offered the best compromise between the highest possible tumor contrast and acceptable signal intensity. To statistically compare the tumor-to-muscle and tumor-to-heart ratios at 1–2 h postinjection achieved with the ^64^Cu- and ^68^Ga-labeled ligands **NECT-330**, **NECT-334,** and **NECT-346** to [^**64**^ **Cu]­Cu-NECT-224** and [^**68**^ **Ga]­Ga-NECT-224**, respectively (Tables S2 and S3), an ordinary one-way ANOVA was conducted using the Šidák correction model (*p ≤ 0.05, **p ≤ 0.01, ***p ≤ 0.001).

For the PET experiment with the ADAM10/17 inhibitor **INCB3619**, the HT-1376 tumor-bearing mice were injected intraperitoneally with either 100 nmol of **INCB3619** (inhibitor, approximately 1.4 mg/kg) in 100 μL of 0.9% NaCl or only 100 μL of 0.9% NaCl (control) at 17 and 1 h before the injection of the radiotracer.

### First-in-Human Application

[^**64**^ **Cu]­Cu-NECT-224** was synthesized in a manual process. The [^64^Cu]­CuCl_2_ solution (20–150 μL, 0.01 M HCl) was mixed at a ratio of 2:1 with sodium acetate buffer (0.5 M, pH 5.0) and precursor (30 μg, 1 μg/μL). The resulting mixture was diluted to 1 mL with H_2_O_suprapur_ (1.00473 Supelco) and transferred into a sterile, evacuated 10 mL vial (Curium GmbH). The labeling was performed at 75 °C for 20 min. The labeling solution was diluted with 3 mL of 0.9% saline and transferred onto a Sep-Pak C8 light (Waters GmbH, WAT036770). The cartridge was washed with 3 mL of water for injection (Ampuwa). Finally, the product was eluted into a sterile 10 mL vial with 1.0 mL of 50% ethanol for injection (PZN 03837980) and formulated with 7 mL 0.9% PBS-buffered saline (B. Braun) via a 0.22 μm sterile filter (Vented Millex, SLGVV255F). The molar activity was determined to be 50.6–60.6 MBq/nmol at a radiochemical yield of 81–85% and a radiochemical purity of greater than 98% (*n* = 3). All quality control tests were performed as a prerequisite for human use, including radiochemical purity, chemical purity, endotoxins, and sterility testing.

The PET/CT scan was performed at 67 min and 16.5 h after the injection of 251 MBq of [^**64**^ **Cu]­Cu-NECT-224** on a Siemens Biograph Vision 600 instrument (Siemens Healthineers, Knoxville, TN, USA). Emission PET scan was obtained using continuous bed motion with speeds of 1.4 mm/s (67 min p.i.) and 0.9 mm/s (16.5 h p.i.) from just above the skull base to the mid-thigh in a caudocranial direction. Images were reconstructed using the TrueX algorithm with 4 iterations, 5 subsets, and a time-of-flight (TOF) application, without postreconstruction filtering (all-pass). A low-dose CT was acquired (X-ray tube current of 11 mAs, tube voltage of 120 kV, spiral pitch factor, and 3.0 mm slice thickness), which was used for scatter correction of the subsequent PET scan.

## Supplementary Material



## Notes

Additional experimental methods, figures, schemes, tables, and analytical data. Chemistry section; Binding of **NECT-224** to hNectin-1, -2, -3, and -4; stability of ^64^Cu-labeled **NECT-330**, **NECT-334**, and **NECT-346** in human plasma; shedding of Nectin-4 from HT-1376 cells; cell binding and internalization of the ^68^Ga-labeled ligands using UC and TNBC cells; TACs for normal organs in HT-1376 tumor-bearing mice; PET images and image-derived uptake values of [^**64**^ **Cu]­Cu-NECT-224** and [^**64**^ **Cu]­Cu-NECT-346** in 5637 and MDA-MB-231 tumor-bearing mice; TACs for normal organs in 5637 and MDA-MB-231 tumor-bearing mice; TACs for normal organs in HT-1376 tumor-bearing mice obtained for the ^61^Cu-labeled ligands; PET images and image-derived uptake values of [^**64**^ **Cu]­Cu-NECT-224** with and without coadministration of the ADAM10/17 inhibitor INCB3619; TACs for normal organs in HT-1376 tumor-bearing mice of [^**64**^ **Cu]­Cu-NECT-224** with and without inhibitor INCB3619; TACs for normal organs in MDA-MB-468 tumor-bearing mice; insights into activity uptake in kidneys from the first-in-human application of [^**64**^ **Cu]­Cu-NECT-224**; exemplary TAC for the submandibularis in MDA-MB-468 tumor-bearing mice of [^**64**^ **Cu]­Cu-NECT-224**; regions of interest (ROIs) generated in Rover for extraction of tissue-specific uptake values from PET images; general synthetic route to the Nectin-4 ligands; analytical data of the bicyclic peptides; SUVmean and SUVmean ratios for the ^64^Cu- and ^68^Ga-labeled ligands in an HT-1376 tumor xenograft model; SUVmean and SUVmean ratios for the ^64^Cu- and ^68^Ga-labeled ligands in an MDA-MB-468 xenograft model; SUVmean and SUVmean ratios for [^**64**^ **Cu]­Cu-NECT-224** and [^**64**^ **Cu]­Cu-NECT-346** in 5637 and MDA-MB-231 tumor xenograft models; SUVmean and SUVmean ratios for [^**61**^ **Cu]­Cu-NECT-224** and [^**61**^ **Cu]­Cu-NECT-346** in HT-1376 tumor xenograft models; blood kinetic parameters for the ^64^Cu- and ^61^Cu-labeled ligands in an HT-1376 tumor xenograft model; blood kinetic parameters for the ^68^Ga-labeled ligands in an HT-1376 tumor xenograft model; Compound characterization data (PDF)

## Abbrivation

ADC: antibody-drug conjugate
PET: positron emission tomography
PDC: peptide-drug conjugate
SMDC: small molecule-drug conjugate
ROI: region of interest
SPECT: single-photon emission computed tomography
SPR: surface plasmon resonance
SUV: standardized uptake value
TAC: time-activity curve
TATA: 1,3,5-triacryloyl-1,3,5-triazinane
TNBC: triple-negative breast cancer
UC: urothelial carcinoma

## References

[1] Perez H. L., Cardarelli P. M., Deshpande S., Gangwar S., Schroeder G. M., Vite G. D., Borzilleri R. M. (2014). Antibody-Drug Conjugates: Current
Status and Future Directions. Drug Discovery
Today.

[2] Do
Pazo C., Nawaz K., Webster R. M. (2021). The Oncology Market for Antibody-Drug
Conjugates. Nat. Rev. Drug Discovery.

[3] Liu K., Li M., Li Y., Li Y., Chen Z., Tang Y., Yang M., Deng G., Liu H. (2024). A review of the clinical
efficacy of FDA-approved antibody–drug conjugates in human
cancers. Mol. Cancer.

[4] Colombo R., Tarantino P., Rich J. R., LoRusso P. M., de Vries E. G. E. (2024). The
Journey of Antibody-Drug Conjugates: Lessons Learned from 40 Years
of Development. Cancer Discovery.

[5] Wang R., Hu B., Pan Z., Mo C., Zhao X., Liu G., Hou P., Cui Q., Xu Z., Wang W. (2025). Antibody–Drug
Conjugates (ADCs): current and future biopharmaceuticals. J. Hematol. Oncol..

[6] Fda Approved Antibody-Drug Conjugates (Adcs) by 2026 (Biopharma Peg).

[7] Bouleftour W., Guillot A., Magne N. (2022). The Anti-Nectin
4: A Promising Tumor
Cells Target. A Systematic Review. Mol. Cancer
Ther..

[8] Heath E. I., Rosenberg J. E. (2021). The Biology and Rationale of Targeting Nectin-4 in
Urothelial Carcinoma. Nat. Rev. Urol..

[9] Challita-Eid P. M., Satpayev D., Yang P., An Z., Morrison K., Shostak Y., Raitano A., Nadell R., Liu W., Lortie D. R., Capo L., Verlinsky A., Leavitt M., Malik F., Avina H., Guevara C. I., Dinh N., Karki S., Anand B. S., Pereira D. S., Joseph I. B., Donate F., Morrison K., Stover D. R. (2016). Enfortumab
Vedotin Antibody-Drug Conjugate Targeting Nectin-4 Is a Highly Potent
Therapeutic Agent in Multiple Preclinical Cancer Models. Cancer Res..

[10] Reymond N., Fabre S., Lecocq E., Adelaide J., Dubreuil P., Lopez M. (2001). Nectin4/Prr4, a New Afadin-Associated
Member of the Nectin Family
That Trans-Interacts with Nectin1/Prr1 through V Domain Interaction. J. Biol. Chem..

[11] Information for Padcev from European Medicines Agency.

[12] Fda Approves Enfortumab Vedotin-Ejfv with Pembrolizumab for Locally Advanced or Metastatic Urothelial Cancer.

[13] Chavda V. P., Solanki H. K., Davidson M., Apostolopoulos V., Bojarska J. (2022). Peptide-Drug Conjugates: A New Hope for Cancer Management. Molecules.

[14] Dean T. T., Jelu-Reyes J., Allen A. C., Moore T. W. (2024). Peptide-Drug Conjugates:
An Emerging Direction for the Next Generation of Peptide Therapeutics. J. Med. Chem..

[15] Cazzamalli S., Puca E., Neri D. P. (2025). Present and Future
of Drug Conjugates
for Cancer Therapy. Nat. Cancer.

[16] Paulus J., Sewald N. (2024). Small molecule–
and peptide–drug conjugates
addressing integrins: A story of targeted cancer treatment. J. Pept. Sci..

[17] Heinis C., Rutherford T., Freund S., Winter G. (2009). Phage-Encoded Combinatorial
Chemical Libraries Based on Bicyclic Peptides. Nat. Chem. Biol..

[18] Angelini A., Morales-Sanfrutos J., Diderich P., Chen S., Heinis C. (2012). Bicyclization
and Tethering to Albumin Yields Long-Acting Peptide Antagonists. J. Med. Chem..

[19] Mudd G. E., Brown A., Chen L., van Rietschoten K., Watcham S., Teufel D. P., Pavan S., Lani R., Huxley P., Bennett G. S. (2020). Identification and Optimization of
Epha2-Selective Bicycles for the Delivery of Cytotoxic Payloads. J. Med. Chem..

[20] Upadhyaya P., Kristensson J., Lahdenranta J., Repash E., Ma J., Kublin J., Mudd G. E., Luus L., Jeffrey P., Hurov K., McDonnell K., Keen N. (2022). Discovery and Optimization
of a Synthetic Class of Nectin-4-Targeted Cd137 Agonists for Immuno-Oncology. J. Med. Chem..

[21] Noisier A. F. M., Sandmark J., Edfeldt F., Backmark A., Broddefalk J., Wandzik J., Jurva U., Ek M., Johansson C. A., Barlind L., Gunnarsson J., Bigalke J. M., Xue Y., Frolov A. I., Kankkonen C., Roth R. G., Fritsch M., Watcham S., van Rietschoten K., Mudd G. E., Harrison H., Chen L., Skynner M. J., Craik D. J., Chankeshwara S. V., Lemurell M. (2025). Design of Bicyclic
Peptide Tandems Mimicking the Homodimeric
Gdf15 Protein to Inhibit Gdf15-Gfral-Ret Complex Cell Signaling. J. Med. Chem..

[22] Mudd G. E., Scott H., Chen L., van Rietschoten K., Ivanova-Berndt G., Dzionek K., Brown A., Watcham S., White L., Park P. U., Jeffrey P., Rigby M., Beswick P. (2022). Discovery of Bt8009: A Nectin-4 Targeting Bicycle Toxin
Conjugate for the Treatment of Cancer. J. Med.
Chem..

[23] Rigby M., Bennett G., Chen L., Mudd G. E., Harrison H., Beswick P. J., Van Rietschoten K., Watcham S. M., Scott H. S., Brown A. N., Park P. U., Campbell C., Haines E., Lahdenranta J., Skynner M. J., Jeffrey P., Keen N., Lee K. (2022). Bt8009; a Nectin-4 Targeting Bicycle Toxin Conjugate for Treatment
of Solid Tumors. Mol. Cancer Ther..

[24] Speltri G., Badrane I., Napolitano R., Boschi A., Uccelli L., Filippi L., Guidoboni M., Brunelli M., Lancia F., Martini P., Iudicello A., Cittanti C., Bartolomei M., Urso L. (2025). Nectin-4-Targeting
Radiotracers: Novel Theranostic Agents for Precision
Oncology in Cancer. Mol. Diagn. Ther..

[25] Primac I., Tabury K., Tasdogan A., Baatout S., Herrmann K. (2025). The Molecular
Blueprint of Targeted Radionuclide Therapy. Nat. Rev. Clin. Oncol..

[26] Kabunda J., Ndlovu H., Singh K., Sibiya S., Mdanda S., Ramonaheng K., Al-Ibraheem A., Herrmann K., Mokoala K., Sathekge M. (2025). Nectin-4,Bladder
Cancer, and Nuclear Medicine: A Theranostic
Frontier. Semin Nucl. Med..

[27] Huang W., Li L., Liang Y., Yang Q., Mixdorf J. C., Engle J. W., Fan Y., Kang L., Cai W. (2025). Immunopet Imaging of Nectin4 Expression
in Gastric and Bladder Cancer Using [^64^cu]­Cu-Nota-Padcev. Mol. Pharmaceutics.

[28] Huang W., Wang T., Liang Y., Chao F., Yang Q., Barnhart T. E., Engle J. W., Li L., Kang L., Cai W. (2025). A Zirconium-89-Labeled Antibody-Drug
Conjugate Pet Probe for Noninvasive
Monitoring of Nectin4 Expression in Breast Cancer and Lung Cancer. Cell Rep. Phys. Sci..

[29] Huang W., Li L., Chao F., Yang Q., Mixdorf J. C., Engle J. W., Hsu J. C., Kang L., Cai W. (2025). [^64^cu]­Cu-Nota-Ev-F­(Ab’)_2_ Enables Same-Day Immuno-Pet Imaging of Nectin-4 in Triple-Negative
Breast and Urothelial Bladder Cancers. J. Nucl.
Med..

[30] Shao F., Pan Z., Long Y., Zhu Z., Wang K., Ji H., Zhu K., Song W., Song Y., Song X. (2022). Nectin-4-Targeted
Immunospect/Ct Imaging and Photothermal Therapy of Triple-Negative
Breast Cancer. J. Nanobiotechnol..

[31] Campbell D. O., Noda A., Verlinsky A., Snyder J., Fujita Y., Murakami Y., Fushiki H., Miyoshi S., Lacayo S., Cabral E., Yang P., Stover D. R., Joseph I. B. (2016). Preclinical
Evaluation of an Anti-Nectin-4 Immunopet Reagent in Tumor-Bearing
Mice and Biodistribution Studies in Cynomolgus Monkeys. Mol. Imaging Biol..

[32] Zheng D., Huang Y., Li C., Lu Y., Xie Z., Ren Q., Liang Y., Meng X. (2025). A Radiofluorinated
Nanobody Pet Tracer
to Dynamically Visualize Differential Nectin4 Expression in Vivo. J. Med. Chem..

[33] Sun L., Sun Y., Zuo K., Fan L., Wang X., Zhang J., Hu S., Liu X., Li J., Li Y., Shao Z., Xu X., Wu A., Song S. (2025). Pilot Study
of Nectin-4-Targeted
Pet Imaging Agent ^68^ga-Fz-Nr-1 in Triple-Negative Breast
Cancer from Bench to First-in-Human. J. Nucl.
Med..

[34] Duan X., Zhang Z., Xu H., Zhang J., Yan Y., Yang X. (2025). Preclinical Evaluation of an Al^18^f-Radiolabeled Bicyclic
Peptide Targeting Nectin-4. Mol. Pharmaceutics.

[35] Zhang J., Duan X., Chen X., Zhang Z., Sun H., Shou J., Zhao G., Wang J., Ma Y., Yang Y., Tian X., Shen Q., Yu W., He Z., Fan Y., Yang X. (2024). Translational Pet Imaging of Nectin-4
Expression in Multiple Different Cancers with ^68^ga-N188. J. Nucl. Med..

[36] Wan Q., Yuan H., Cai P., Liu Y., Yan T., Wang L., Zhou Z., Zhang W., Liu N. (2024). Effects of
Pegylation on Imaging Contrast of ^68^ga-Labeled Bicyclic
Peptide Pet Probes Targeting Nectin-4. Mol.
Pharmaceutics.

[37] Ge S., Jia T., Shi J., Cao J., Sang S., Li J., Zhang B., Deng S. (2024). A Cutting-Edge ^68^ga-Labeled
Bicyclic Peptide Pet Molecular Probe for Noninvasive Assessment of
Nectin4 Expression. Bioorg. Chem..

[38] Duan X., Xia L., Zhang Z., Ren Y., Pomper M. G., Rowe S. P., Li X., Li N., Zhang N., Zhu H., Yang Z., Sheng X., Yang X. (2023). First-in-Human Study of the Radioligand ^68^ga-N188 Targeting
Nectin-4 for Pet/Ct Imaging of Advanced
Urothelial Carcinoma. Clin. Cancer Res..

[39] Krönke T., Trommer J., Ullrich M., Laube M., Löser R., Kretzschmar J., Urbanova M., Stadlbauer S., Brandt F., Platzek I., Hoberück S., Kotzerke J., Thomas C., Miederer M., Bundschuh R. A., Kopka K., Pietzsch J., Wodtke R. (2025). “Precision
on
Two Wheels” - Structural Refinement of ^64^cu- and ^68^ga-Labeled Bicyclic Peptides Targeting Nectin-4 for Improved
Tumor Imaging: From Preclinical Development to First-in-Human Application. J. Med. Chem..

[40] Brühlmann S. A., Walther M., Kopka K., Kreller M., Kiss O. C. (2026). Coppernostics-Here
We Are Now, Entertain Us!. Pharmaceuticals.

[41] Fabre-Lafay S., Monville F., Garrido-Urbani S., Berruyer-Pouyet C., Ginestier C., Reymond N., Finetti P., Sauvan R., Adelaide J., Geneix J. (2007). Nectin-4 Is a New Histological
and Serological Tumor Associated Marker for Breast Cancer. BMC Cancer.

[42] Fabre-Lafay S., Garrido-Urbani S., Reymond N., Goncalves A., Dubreuil P., Lopez M. (2005). Nectin-4,
a New Serological Breast
Cancer Marker, Is a Substrate for Tumor Necrosis Factor-Alpha-Converting
Enzyme (Tace)/Adam-17. J. Biol. Chem..

[43] Buchanan P. C., Boylan K. L. M., Walcheck B., Heinze R., Geller M. A., Argenta P. A., Skubitz A. P. N. (2017). Ectodomain
Shedding of the Cell Adhesion
Molecule Nectin-4 in Ovarian Cancer Is Mediated by Adam10 and Adam17. J. Biol. Chem..

[44] Powles T., Rosenberg J. E., Sonpavde G. P., Loriot Y., Duran I., Lee J. L., Matsubara N., Vulsteke C., Castellano D., Wu C., Campbell M., Matsangou M., Petrylak D. P. (2021). Enfortumab Vedotin
in Previously Treated Advanced Urothelial Carcinoma. N. Engl. J. Med..

[45] Powles T., Valderrama B. P., Gupta S., Bedke J., Kikuchi E., Hoffman-Censits J., Iyer G., Vulsteke C., Park S. H., Shin S. J. (2024). Enfortumab Vedotin and Pembrolizumab in Untreated Advanced
Urothelial Cancer. N. Engl. J. Med..

[46] Chu C. E., Sjostrom M., Egusa E. A., Gibb E. A., Badura M. L., Zhu J., Koshkin V. S., Stohr B. A., Meng M. V., Pruthi R. S., Friedlander T. W., Lotan Y., Black P. C., Porten S. P., Feng F. Y., Chou J. (2021). Heterogeneity in Nectin4 Expression
across Molecular Subtypes of Urothelial Cancer Mediates Sensitivity
to Enfortumab Vedotin. Clin. Cancer Res..

[47] Klümper N., Ralser D. J., Ellinger J., Roghmann F., Albrecht J., Below E., Alajati A., Sikic D., Breyer J., Bolenz C., Zengerling F., Erben P., Schwamborn K., Wirtz R. M., Horn T., Nagy D., Toma M., Kristiansen G., Buttner T., Hahn O., Grunwald V., Darr C., Erne E., Rausch S., Bedke J., Schlack K., Abbas M., Zschabitz S., Schwab C., Mustea A., Adam P., Manseck A., Wullich B., Ritter M., Hartmann A., Gschwend J., Weichert W., Erlmeier F., Holzel M., Eckstein M. (2023). Membranous
Nectin-4 Expression Frequently Decreases During Metastatic Spread
of Urothelial Carcinoma and Is Associated with Enfortumab Vedotin
Resistance. Clin. Cancer Res..

[48] Mishra A., Sharma A. K., Gupta K., Banka D. S., Johnson B. A., Hoffman-Censits J., Huang P., McConkey D. J., Nimmagadda S. (2026). Nectin-4 Pet
for Predicting Enfortumab Vedotin Dose-Response in Urothelial Carcinoma. Sci. Adv..

[49] Stoddard A., Furey K., Dehlavi G., Mane M. M., Sebastiano J., Zeglis B. M. (2026). Linker Chemistry in Radiopharmaceutical Design. Bioconjugate Chem..

[50] Eder M., Pavan S., Bauder-Wust U., van Rietschoten K., Baranski A. C., Harrison H., Campbell S., Stace C. L., Walker E. H., Chen L., Bennett G., Mudd G., Schierbaum U., Leotta K., Haberkorn U., Kopka K., Teufel D. P. (2019). Bicyclic Peptides as a New Modality
for Imaging and Targeting of Proteins Overexpressed by Tumors. Cancer Res..

[51] Zeindler J., Soysal S. D., Piscuoglio S., Ng C. K. Y., Mechera R., Isaak A., Weber W. P., Muenst S., Kurzeder C. (2019). Nectin-4 Expression
Is an Independent Prognostic Biomarker and Associated with Better
Survival in Triple-Negative Breast Cancer. Front.
Med..

[52] Miyake M., Nishimura N., Ohnishi S., Oda Y., Owari T., Ohnishi K., Morizawa Y., Hori S., Gotoh D., Nakai Y. (2023). Diagnostic and Prognostic Roles of Urine Nectin-2 and
Nectin-4 in Human Bladder Cancer. Cancers.

[53] Nuti E., Casalini F., Santamaria S., Fabbi M., Carbotti G., Ferrini S., Marinelli L., La Pietra V., Novellino E., Camodeca C., Orlandini E., Nencetti S., Rossello A. (2013). Selective Arylsulfonamide Inhibitors
of Adam-17: Hit Optimization and Activity in Ovarian Cancer Cell Models. J. Med. Chem..

[54] Liu X., Fridman J. S., Wang Q., Caulder E., Yang G., Covington M., Liu C., Marando C., Zhuo J., Li Y., Yao W., Vaddi K., Newton R. C., Scherle P. A., Friedman S. M. (2006). Selective Inhibition of Adam Metalloproteases Blocks
Her-2 Extracellular Domain (Ecd) Cleavage and Potentiates the Anti-Tumor
Effects of Trastuzumab. Cancer Biol. Ther..

[55] Brummer T., Pigoni M., Rossello A., Wang H., Noy P. J., Tomlinson M. G., Blobel C. P., Lichtenthaler S. F. (2018). The Metalloprotease
Adam10 (a Disintegrin and Metalloprotease 10) Undergoes Rapid, Postlysis
Autocatalytic Degradation. FASEB J..

[56] Shi X., Yang H., Qi M., Chen W., Guo H., Xiao M., Jiang C., Zhang N., Chen Z., Xu X. (2026). Biodistribution,
Dosimetry, and Preliminary Imaging
of the Novel Nectin-4-Targeting Pet Tracer [^68^ga]­Ga-Fz-Nr-1
in Humans. EJNMMI Res..

[57] Fani M., Nicolas G. P. (2023). ^61^cu-Labeled Radiotracers:
Alternative or
Choice?. J. Nucl. Med..

[58] Nicolas G. P., Lafont M. A., McDougall L., Kaul F., Chirindel A., Weber W., Llmazares A., Johayem A., Baier M., Schubert N., De Rose F., Jaafar-Thiel L., Bauman A., Fani M., Wild D. (2026). First-in-Human
Administration
of [^61^cu]­Cu-Nodaga-Lm3 and Head-to-Head Comparison with
[(68)­Ga]­Ga-Dota-Toc Pet/Ct as Part of the Phase I/Ii Copper Pet in
Net Study. Eur. J. Nucl. Med. Mol. Imaging.

[59] Laforest R., Dehdashti F., Lewis J. S., Schwarz S. W. (2005). Dosimetry of ^60/61/62/64^cu-Atsm: A Hypoxia Imaging Agent for Pet. Eur.
J. Nucl. Med. Mol. Imaging.

[60] Williams H. A., Robinson S., Julyan P., Zweit J., Hastings D. (2005). A Comparison
of Pet Imaging Characteristics of Various Copper Radioisotopes. Eur. J. Nucl. Med. Mol. Imaging.

[61] Miederer M., Pretze M., Abbate E., Hartig A., Böhm K., Sommer U., Hoberück S., Bundschuh R. A., Kotzerke J., Thomas C. (2026). Staging Metastatic
Urothelial Cancer
with Nectin-4 Imaging Using Gallium-68-N188 Pet/Ct. BJU Int..

[62] Jiang C., Wei M., Li P., Hu S., Xu X., Li J., Huang D., Weng W., Yu X., Song S. (2026). A Pilot Study
of Nectin-4-Targeted Radioligand [^68^ga]­Ga-Fznr-1 in Patients
with Pancreatic Ductal Adenocarcinoma: Head-to-Head Comparison with
[^18^f]-Fdg. Eur. J. Nucl. Med. Mol.
Imaging.

[63] Kreller M., Pietzsch H., Walther M., Tietze H., Kaever P., Knieß T., Füchtner F., Steinbach J., Preusche S. (2019). Introduction of the New Center for Radiopharmaceutical
Cancer Research at Helmholtz-Zentrum Dresden-Rossendorf. Instruments.

[64] Thieme S., Walther M., Pietzsch H. J., Henniger J., Preusche S., Mäding P., Steinbach J. (2012). Module-Assisted Preparation of ^64^cu with
High Specific Activity. Appl.
Radiat. Isot..

[65] Thieme S., Walther M., Preusche S., Rajander J., Pietzsch H. J., Lill J. O., Kaden M., Solin O., Steinbach J. (2013). High Specific
Activity ^61^cu Via ^64^zn­(P,Alpha)^61^cu Reaction at Low Proton Energies. Appl. Radiat.
Isot..

[66] Brühlmann S. A., Walther M., Kopka K., Kreller M. (2024). Production of the Pet
Radionuclide ^61^cu Via the ^62^ni­(P,2n)^61^cu Nuclear Reaction. EJNMMI Radiopharm. Chem..

[67] Brandt F., Ullrich M., Laube M., Kopka K., Bachmann M., Löser R., Pietzsch J., Pietzsch H. J., van den
Hoff J., Wodtke R. (2022). “Clickable” Albumin Binders for Modulating
the Tumor Uptake of Targeted Radiopharmaceuticals. J. Med. Chem..

