# Supplementary material for: “Imaging Triplets on Two Wheels”: 61Cu‑, 64Cu‑, and 68Ga-labeled Bicyclic Peptides Targeting Nectin‑4 Enable High-Contrast PET Imaging of Urothelial Carcinoma and Triple-Negative Breast Cancer
Source: Bioconjug Chem. 2026 Aug 22;37(9):1887–904. doi: 10.1021/acs.bioconjchem.6c00366 (PMC13587596; doi:10.1021/acs.bioconjchem.6c00366)
Supplement: Supplementary file 1 [file bc6c00366_si_001.pdf]

Supporting Information

"Imaging triplets on two wheels" - ^{61}Cu-, ^{64}Cu-, and ^{68}Ga-labeled bicyclic peptides targeting Nectin-4 enable high-contrast PET imaging of urothelial carcinoma and triple-negative breast cancer

Johanna Mauersberger,[a,#] Tobias Krönke,[a,#] Martin Ullrich,[a] Markus Laube,[a] Florian Brandt,[b] Marc Pretze,[b] Fabian Lohaus,[c] Sebastian Hoberück,[b] Christian Thomas,[d] Matthias Miederer,[a,e,f] Ralph A. Bundschuh,[a,b,g] Sven Stadlbauer,[a,h] Klaus Kopka,[a,e,g,h] Jens Pietzsch,[a,h] Robert Wodtke[a,*]

[a] Helmholtz-Zentrum Dresden-Rossendorf, Institute of Radiopharmaceutical Cancer Research, Bautzner Landstraße 400, 01328 Dresden, Germany

[b] University Hospital Carl Gustav Carus at the Technische Universität Dresden, Klinik und Poliklinik für Nuklearmedizin, Fetscherstraße 74, 01307 Dresden, Germany

[c] University Hospital Carl Gustav Carus at the Technische Universität Dresden, Klinik und Poliklinik für Strahlentherapie und Radioonkologie, Fetscherstraße 74, 01307 Dresden, Germany

[d] University Hospital Carl Gustav Carus at the Technische Universität Dresden, Klinik und Poliklinik für Urologie, Fetscherstraße 74, 01307 Dresden, Germany

[e] National Center for Tumor Diseases (NCT), NCT/UCC Dresden, a partnership between DKFZ, Faculty of Medicine and University Hospital Carl Gustav Carus, TUD Dresden University of Technology & Helmholtz-Zentrum Dresden-Rossendorf (HZDR), 01307 Dresden, Germany.

[f] German Cancer Research Center (DKFZ), 69120 Heidelberg, Germany

[g] German Cancer Consortium (DKTK), Partner Site Dresden, 01307 Dresden, Germany

[h] Technische Universität Dresden, School of Science, Faculty of Chemistry and Food Chemistry, Mommsenstraße 4, 01069 Dresden, Germany

# These authors contributed equally to this work.
* Corresponding author (r.wodtke@hzdr.de)

Table of Contents

Chemistry 3

Chromatography 3

General solid-phase syntheses of peptides 4

Figure S1: **Binding of NECT-224 to hNectin-1, -2, -3, and -4** **6**

Figure S2: **Stability of ^{64}Cu-labeled NECT-330, NECT-334, and NECT-346 in human plasma** **7**

Figure S3: **Shedding of Nectin-4 from HT-1376 cells** **8**

Figure S4: **Cell binding and internalization of the ^{68}Ga-labeled ligands using UC and TNBC cells** **9**

Figure S5: **TACs for normal organs in HT-1376 tumor bearing mice** **10**

Figure S6: **PET images and image-derived uptake values of [^{64}Cu]Cu-NECT-224 and [^{64}Cu]Cu-NECT-346 in 5637 and MDA-MB-231 tumor bearing mice** **11**

Figure S7: **TACs for normal organs in 5637 and MDA-MB-231 tumor bearing mice** **12**

Figure S8: **TACs for normal organs in HT-1376 tumor bearing mice obtained for the ^{61}Cu-labeled ligands** **13**

Figure S9: **PET images and image-derived uptake values of [^{64}Cu]Cu-NECT-224 with and without co-administration of ADAM10/17 inhibitor INCB3619** **14**

Figure S10: **TACs for normal organs in HT-1376 tumor bearing mice of [^{64}Cu]Cu-NECT-224 with and without inhibitor INCB3619** **15**

Figure S11: **TACs for normal organs in MDA-MB-468 tumor bearing mice** **16**

Figure S12: **Insights into activity uptake in kidneys from first-in-human application of [^{64}Cu]Cu-NECT-224** **17**

Figure S13: **Exemplary TAC for the submandibularis in MDA-MB-468 tumor bearing mice of [^{64}Cu]Cu-NECT-224** **18**

Figure S14: **Regions of interest (ROIs) generated in Rover for extraction of tissue-specific uptake values from PET images** **19**

Scheme S1: **General synthetic route to the Nectin-4 ligands** **20**

Table S1: **Analytical data of the bicyclic peptides** **21**

Table S2: **SUVmean and SUVmean ratios for the ^{64}Cu- and ^{68}Ga-labeled ligands in a HT-1376 tumor xenograft model** **22**

Table S3: **SUVmean and SUVmean ratios for the ^{64}Cu- and ^{68}Ga-labeled ligands in a MDA-MB-468 xenograft model** **23**

Table S4: **SUVmean and SUVmean ratios for [^{64}Cu]Cu-NECT-224 and [^{64}Cu]Cu-NECT-346 in 5637 and MDA-MB-231 tumor xenograft models** **24**

Table S5: **SUVmean and SUVmean ratios for [^{61}Cu]Cu-NECT-224 and [^{61}Cu]Cu-NECT-346 in HT-1376 tumor xenograft models** **25**

Table S6: **Blood kinetic parameters for the ^{64}Cu- and ^{61}Cu-labeled ligands in a HT-1376 tumor xenograft model** **26**

Table S7: **Blood kinetic parameters for the ^{68}Ga-labeled ligands in a HT-1376 tumor xenograft model** **27**

Compound characterization data **28**

Bicyclic peptides 28

Radiolabeled bicyclic peptides 34

References **37**

Chemistry

Chromatography

The HPLC system used was a LC-20A Prominence HPLC by Shimadzu, consisting of degasser unit DGU-20A5R, two separate pumping units LC-A20R, sample manager SIC-20ACHT, column oven CTO-20AC, PDA-detector SPD-M20A, communication-bus module CBM-20A and fraction collector FRC-10A. Two Aeris Peptide 5 µm XB-C18 columns (100 Å, 250x4.6 mm and 250x21.2 mm) were used as the stationary phases for analytical and preparative RP-HPLC, respectively. A binary gradient system of 0.1% CF_3COOH/water (solvent A) and 0.1% CF_3COOH/CH_3CN (solvent B) at a flow rate of 1 mL/min (analytical) or 10 mL/min (preparative) served as the eluent. For determining the purity of the bicyclic peptides with analytical RP-HPLC, the following gradient was applied: 25% eluent B for 5 min, 25% to 75% eluent B in 25 min, 75% to 95% eluent B in 1 min, 95% eluent B for 5 min, 95% to 25% eluent B in 1 min, and 25% eluent B for 5 min. High resolution mass spectra (HRMS) of the peptides were obtained on either of the two following two systems:

- Orbitrap mass spectrometer using electrospray ionization: syringe pump coupled to Thermo Scientific Orbitrap Exploris 120. The measurements were performed in direct injection mode using an eluent consisting of: 0.1 % formic acid in MeOH/Water (50:50 v/v); flow rate 3 µL/min.
- Q-TOF MS using electrospray ionization: Agilent 1260 Infinity II HPLC (Santa Clara, California, USA; pump G7104C, autosampler G7129C, column oven G7116A, DAD detector G7117C) coupled to a γ detector Gabi Star (Raytest Isotopenmeßgeräte GmbH, Straubenhardt, Germany) followed by accurate mass Revident Q-TOF LC/Q-TOF G6575A. The measurements were performed in bypass mode using an eluent consisting of (A): CH_3CN and (B): 0.1% formic acid in H2O; flow rate 0.2 mL/min. A reference mass solution containing hexakis(1H,1H,3H-tetrafluoropropoxy)phosphazene and purine was continuously coinjected via dual AJS ESI source. The system was operated using Agilent Masshunter Workstation 3.6 – LC/MS data acquisition software (Version 12.0), and data evaluation was performed using Agilent Masshunter Workstation 3.6 Qualitative Analysis software (Version 12.0 Update 1).

For UPLC-DAD-MS (reaction monitoring and purity analysis), a system from Waters (ACQUITY UPLC I class system including an ACQUITY UPLC PDA e λ detector coupled to a Xevo TQ-S mass spectrometer) was used. An ACQUITY UPLC BEH C18 column (1.7 µm,

130 Å, 100x2.1 mm, equipped with a ACQUITY UPLC BEH C18 VanGuard Pre-column, 1.7 µm, 130 Å, 5x2.1 mm) was used as stationary phase. A binary gradient system of 0.1% CH_3COOH/water (solvent A) and 0.1% CH_3COOH in CH_3CN/CH_3OH (1:1, v/v, solvent B) at a flow rate of 0.4 mL/min served as the eluent.

Analytical radio-HPLC after $^{64}Cu/^{68}Ga$-labeling was performed on a Series 1200 device (Agilent Technologies, Santa Clara, CA, USA) equipped with a GABI ß/γ-ray detector (Raytest, Straubenhardt, Germany). Eluent A: 0.1% (v/v) trifluoroacetic acid in H_2O; eluent B: 0.1% (v/v) trifluoroacetic acid in acetonitrile; HPLC system: Aeris Peptide XB-C18, 100 Å, 5 µm, 250 × 4.6 mm (Phenomenex); gradient elution using 30% eluent B for 2 minutes, 30% eluent B to 90% eluent B in 12 minutes, 95% eluent B for 2 minutes and 95% eluent B to 30% eluent B in 1 minutes, 1 mL/minute, room temperature, recovery of activity (decay-corrected) was > 95 %.

Analytical radio-HPLC after ^{61}Cu-labeling was performed on a Series 1200 device (Agilent Technologies, Santa Clara, CA) equipped with a Ramona ß/γ-ray detector (Raytest, Straubenhardt, Germany). Eluent A: 0.1% (v/v) trifluoroacetic acid in H2O; eluent B: 0.1% (v/v) trifluoroacetic acid in acetonitrile; HPLC system: Zorbax SB-C18, 300 Å, 4 µm, 250 mm × 9.4 mm (Agilent); gradient elution using 5% eluent B for 5 min, 5% eluent B to 95% eluent B in 10 min, 95% eluent B for 5 min and 95% eluent B to 5% eluent B in 5 min, 3 mL/ min, 50 °C.

General solid-phase syntheses of peptides

All peptides were synthesized by automated microwave-assisted solid phase peptide synthesis (SPPS) using a Biotage Initiator+ Astra with standard protocols for resin loading, amino acid coupling and Fmoc removal, which were previously described in detail.[1] In brief, Fmoc-Rink Amide resin was used as polymeric support and peptide assembly was performed by repetitive cycles of Fmoc removal (20% piperidine in DMF) and coupling (4 eq. amino acid, 4 eq. HATU, 8 eq. DIPEA, in DMF). Coupling of Fmoc-PEG_2-OH (2eq.) and Fmoc-PEG_8-OH (2 eq,) was manually performed using HATU (2 eq.) and DIPEA (4 eq.) in DMF. N-terminal acylation with *R*-NODAGA-$(tBu)_3$ (2 eq.) was manually performed using HATU (2 eq.) and DIPEA (4 eq.) in DMF. Cleavage from the resin and concomitant removal of all protecting groups was realized by treatment with TFA/H_2O/TIPS/Anisol (92.5:2.5:2.5:2.5, v/v/v/v, 1 h at 40°C) and after removal of TFA (with N_2 flow, TFA trapping in KOH solution), the peptides were precipitated with ice-cold diethyl ether. The linear peptides were dissolved in a mixture of CH_3CN/H_2O (1:1, v/v) and diluted with NH_3 (1% in H_2O) to a final concentration of ≈1 mM. The cyclization was started by the addition of TATA (1.3 eq.). After completion of the reaction (≈1 h), the pH value was adjusted to 2.0 with TFA/water (1:9, v/v). The solution was then lyophilized and the crude products were purified by RP-HPLC.

The non-radioactive metal complexes **^{nat}Cu-NECT-224** and **^{nat}Ga-NECT-224** were prepared under the same conditions as applied for radiolabeling (see main manuscript) using $CuSO_4$ (1.2 eq.) and $GaCl_3$ (1.2 eq.). The reaction mixtures were then lyophilized and the crude products were purified by RP-HPLC.

Figure S1: Binding of NECT-224 to hNectin-1, -2, -3, and -4

A) SPR sensorgrams (single cycle) for binding of **NECT-224** (200 nM) to immobilized hNectin-1, -2, -3, and -4 on flow cell 1, 2, 3, and 4, respectively. Signals of y-axis are adjusted to the baseline (before injection of the analyte) **B**) Relative binding response of **NECT-224**, -**330**, -**334** and -**346** to hNectin-1, -2, -3, and -4 based in evaluating the binding response in the dissociation phase (60 s after injection of analyte) compared to the baseline (before injection of analyte).

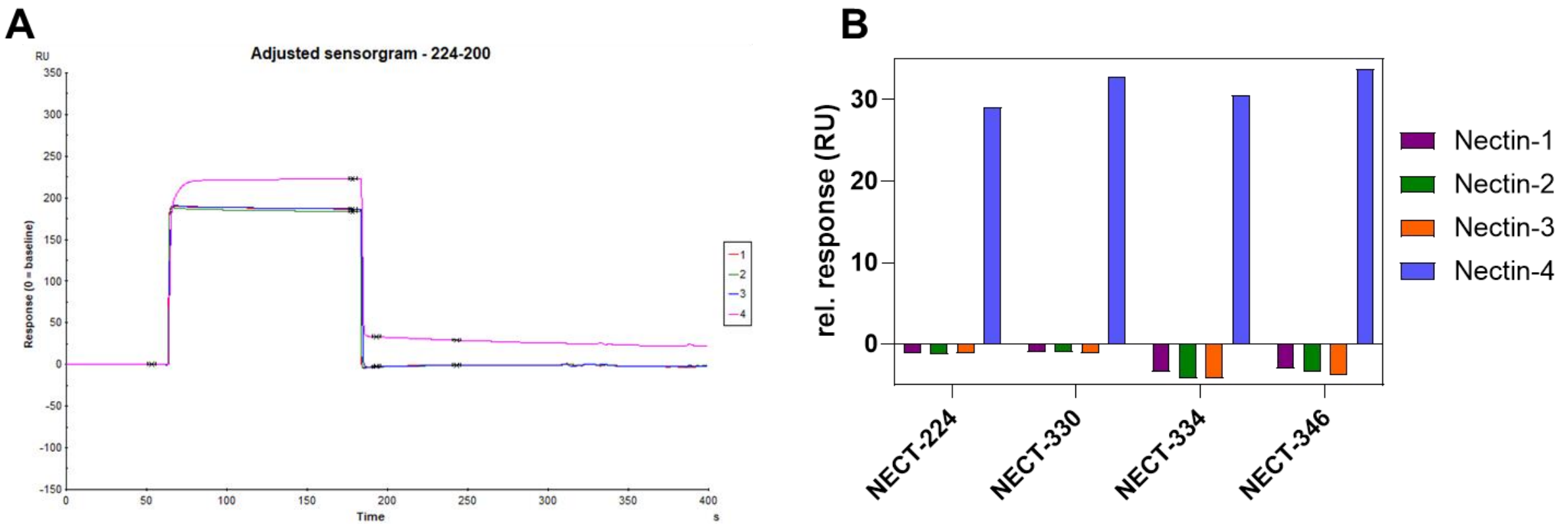

Figure S2: Stability of ^{64}Cu-labeled NECT-330, NECT-334, and NECT-346 in human plasma

Radio-HPLC chromatograms at 24 h were staggered by nudging the data points along the x- and y-axes.

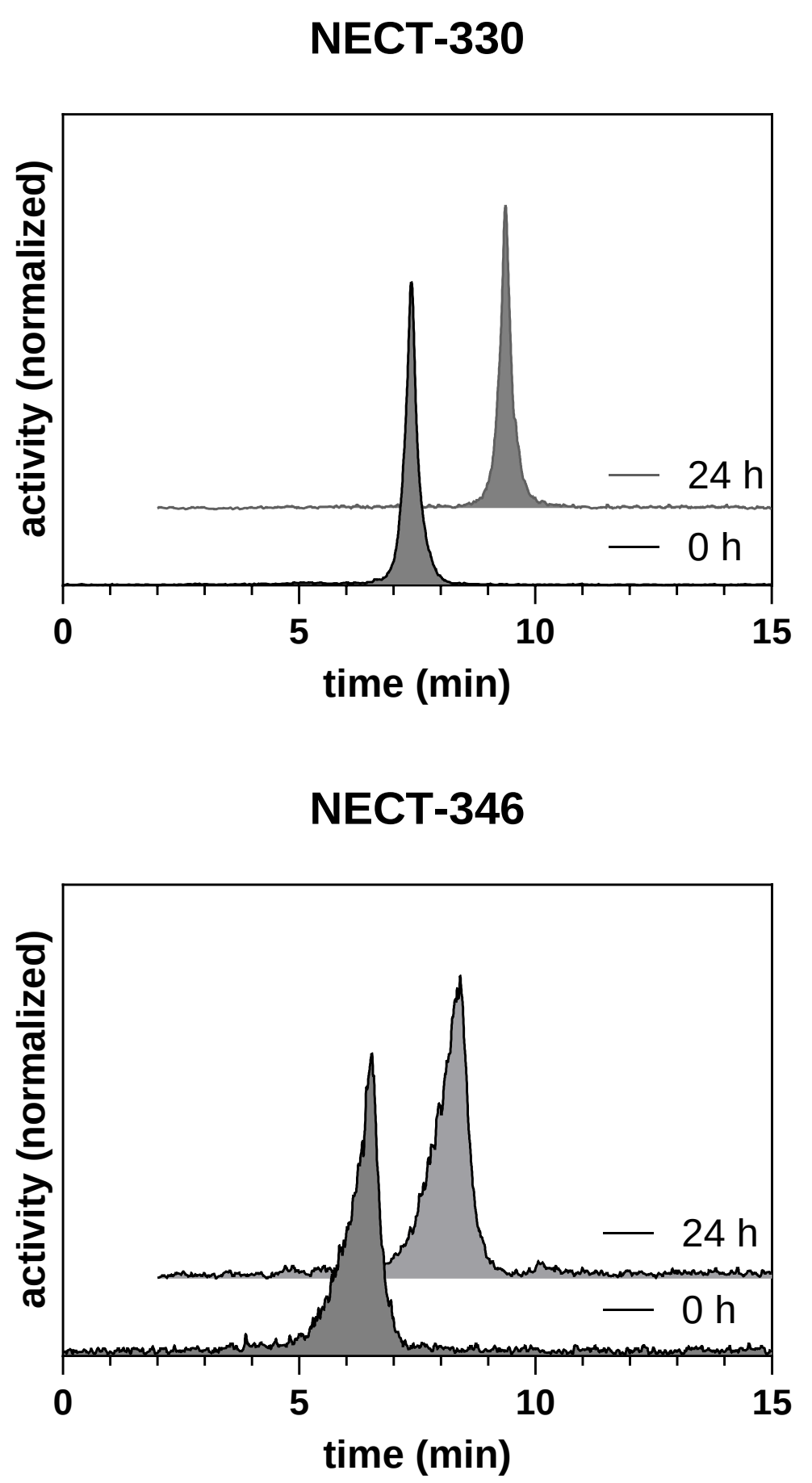

Figure S3: Shedding of Nectin-4 from HT-1376 cells

(A) Correlation of soluble Nectin-4 (sNectin-4) in HT-1376 cell culture supernatant per well over time. (**E**) Direct comparison of quantities of Nectin-4 in cell layer and sNectin-4 per hour in corresponding volume of cell culture supernatant. Concentration of sNectin-4 detected by ELISA in the supernatant of HT-1376 cells incubated with different concentrations of (**C**) **JG26** or (**D**) **INCB3619**. The cell culture supernatant was tested after 2 h, 6 h, 24 h and 48 h of incubation. Bars represent single measurements. (**E**) Results of a radioligand binding performed with and without 100 nM **INCB3619** (15 min pre-incubation period) with HT-1376 cells.

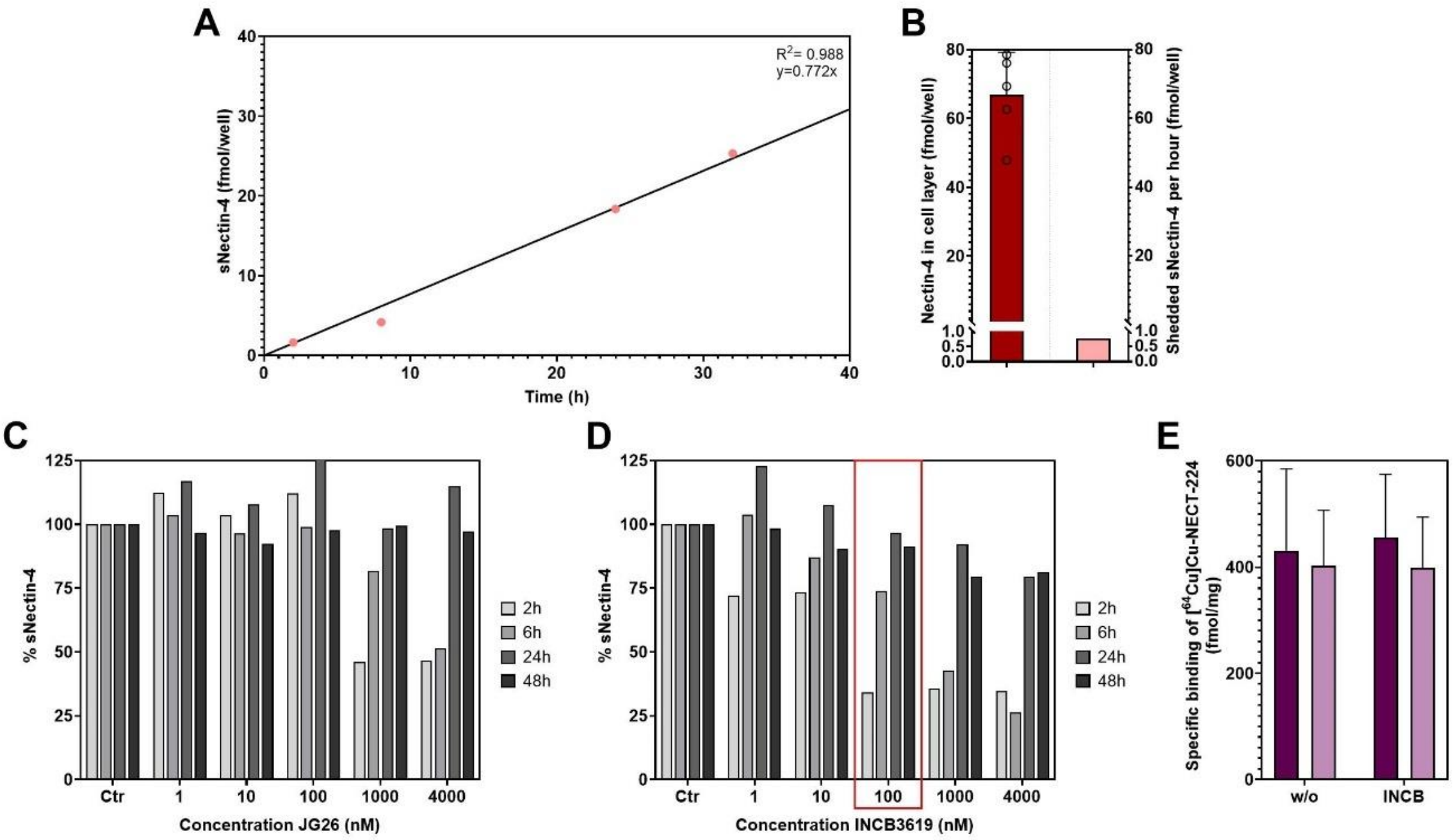

Figure S4: Cell binding and internalization of the ^{68}Ga-labeled ligands using UC and TNBC cells

A/D) Specific cell binding of the ^{68}Ga-labeled ligands to intact HT-1376 (A) and TNBC cells (D) (MDA-MB-468 in muted colors and MDA-MB-231 in light colors). Data are shown for specific binding after washing with PBS (solid bars) and after washing with acidic glycine buffer ("acid" wash, hatched bars), with mean values given at the bars. Data shown are mean values (±SD) of one experiment performed in sextuplicate. **B/E**) Saturation binding of **[^{68}Ga]Ga-NECT-224** toward intact HT-1376 (**B**) and MDA-MB-468 cells (**E**). **C/F**) Saturation binding of **[^{68}Ga]Ga-NECT-346** toward intact HT-1376 (**C**) and intact MDA-MB-468 cells (**F**). Data for total binding, non-specific binding and specific binding are shown in black, gray, and colored circles respectively. Non-specific binding was determined in presence of 1 µM **NECT-214**. Regression analysis was performed as described in the experimental section.

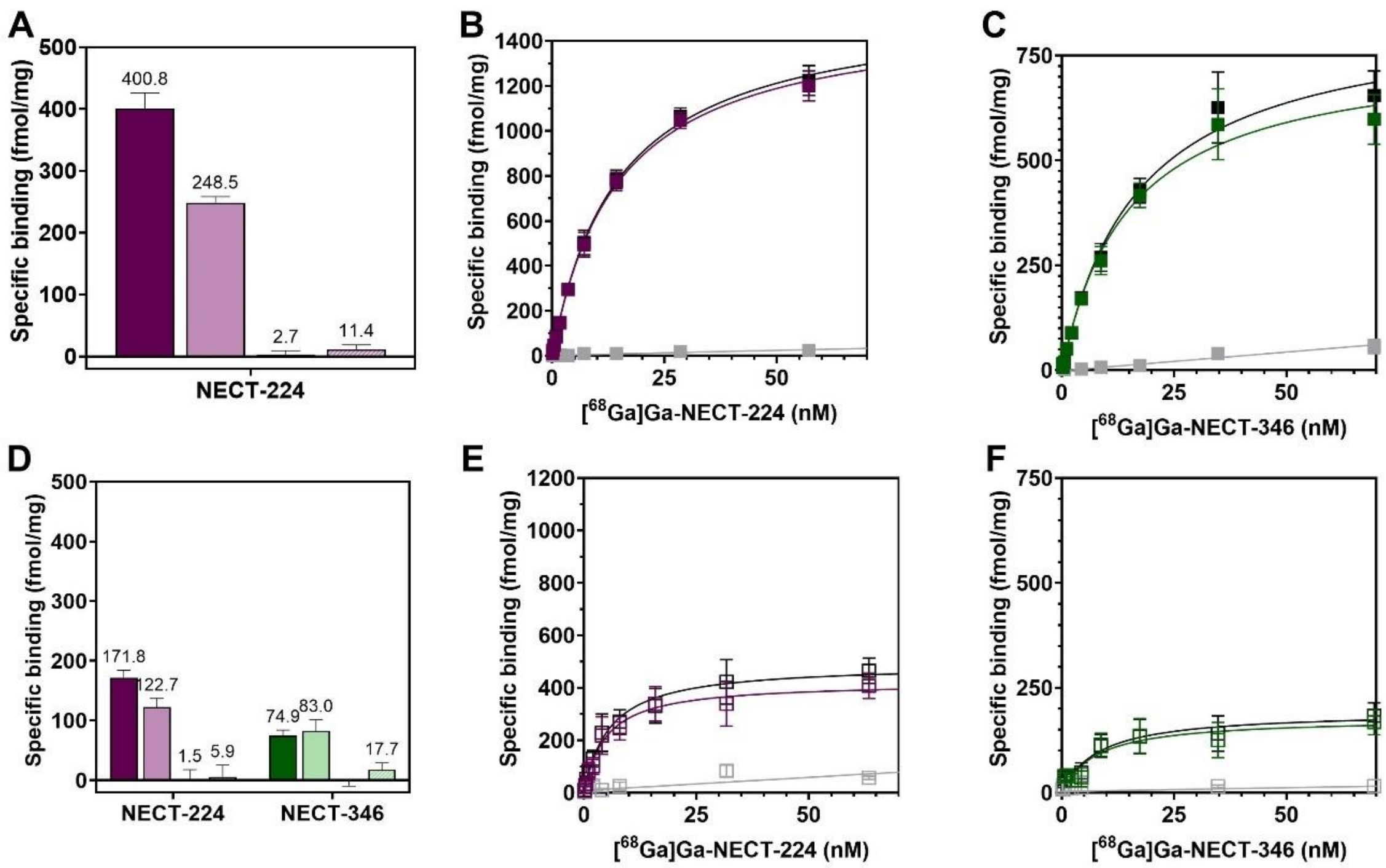

Figure S5: TACs for normal organs in HT-1376 tumor bearing mice

Time-activity curves (SUVmean, decay-corrected, as a function of time up to 2 h) of the muscle (**A/D**), kidney (**B/E**), and liver (**C/F**) obtained from quantitative analysis of PET images are depicted for the ^{64}Cu-labeled (**A**-**C**) and ^{68}Ga-labeled ligands (**D-F**). Color coding according to Figure 4 in the main article.

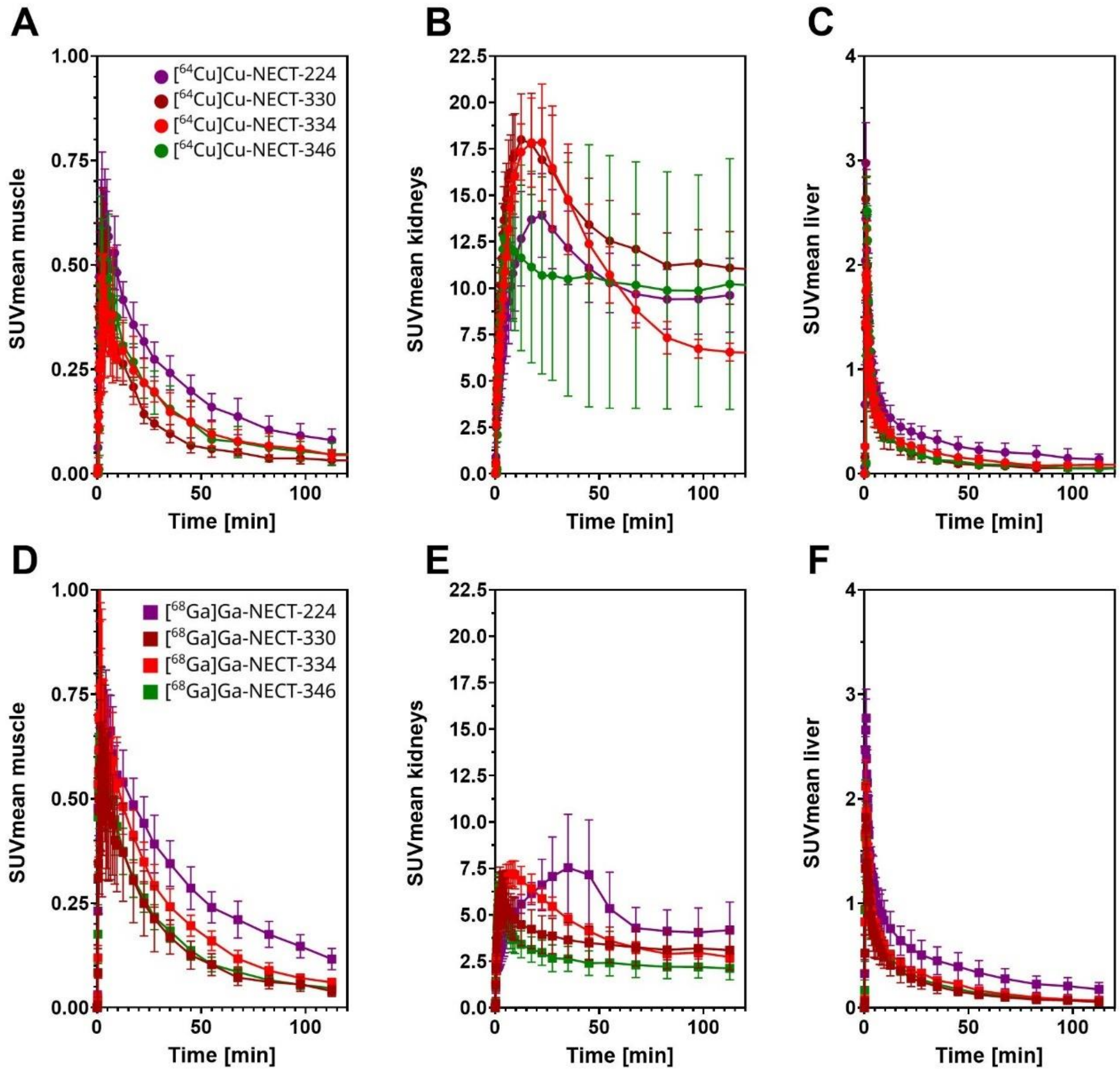

Figure S6: PET images and image-derived uptake values of [^{64}Cu]Cu-NECT-224 and [^{64}Cu]Cu-NECT-346 in 5637 and MDA-MB-231 tumor bearing mice

A/B) PET/CT images at 1-2 h after intravenous injection of the indicated radioligands in 5637 (**A**) and MDA-MB-231 (**B**) tumor-bearing mice. 6-9 MBq/animal (0.3-0.45 nmol/animal) were injected. Images are presented as maximum intensity projections with a common scale. Anatomical positions of the tumor (tu), kidney (ki), and urinary bladder (ub) are shown. **C/D**) Time-activity curves (SUVmean, decay-corrected, as a function of time up to 2 h) for the blood content of the heart (**C**) and tumor (**D**) obtained from quantitative analysis of PET images are depicted. **E/F**) Time-resolved SUVmean ratios up to 2 h p.i. including tumor-to-muscle (**E**) and tumor-to-heart (**F**). **G**) Time-resolved body retention in % of the initial dose up to 2 h p.i.. Data points in **C-G** represent mean values (±SD) measured in groups of 5637 and MDA-MB-231 tumor-bearing mice (n=3 for all except for **[^{64}Cu]Cu-NECT-224** in 5637 n=4).

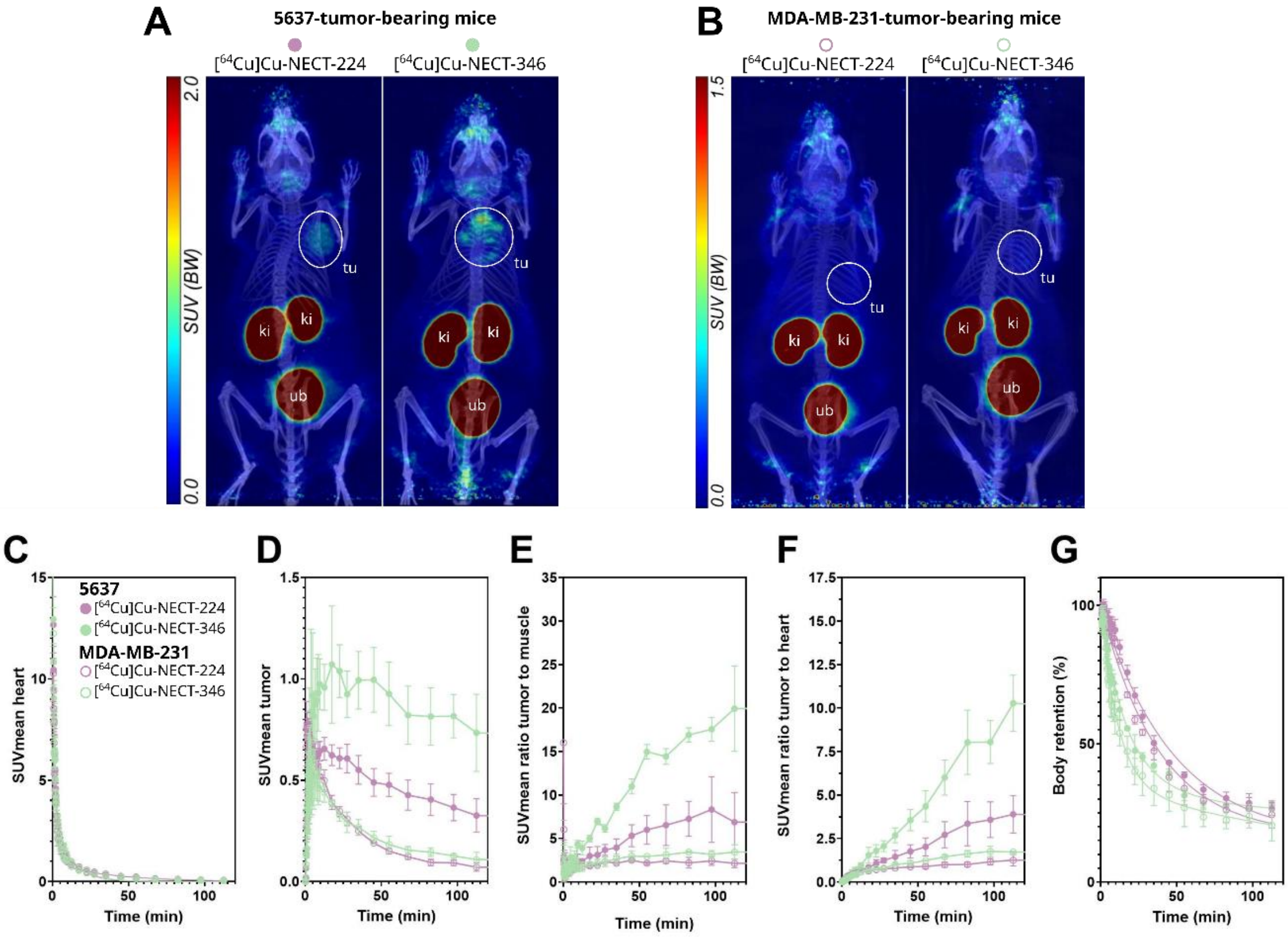

Figure S7: TACs for normal organs in 5637 and MDA-MB-231 tumor bearing mice

Time-activity curves (SUVmean, decay-corrected, as a function of time up to 2 h) of the muscle (**A**), kidney (**B**), and liver (**C**) obtained from quantitative analysis of PET images are depicted for the ligands **[^{64}Cu]Cu-NECT-224** and **[^{64}Cu]Cu-NECT-346**. Color coding according to Figure S6.

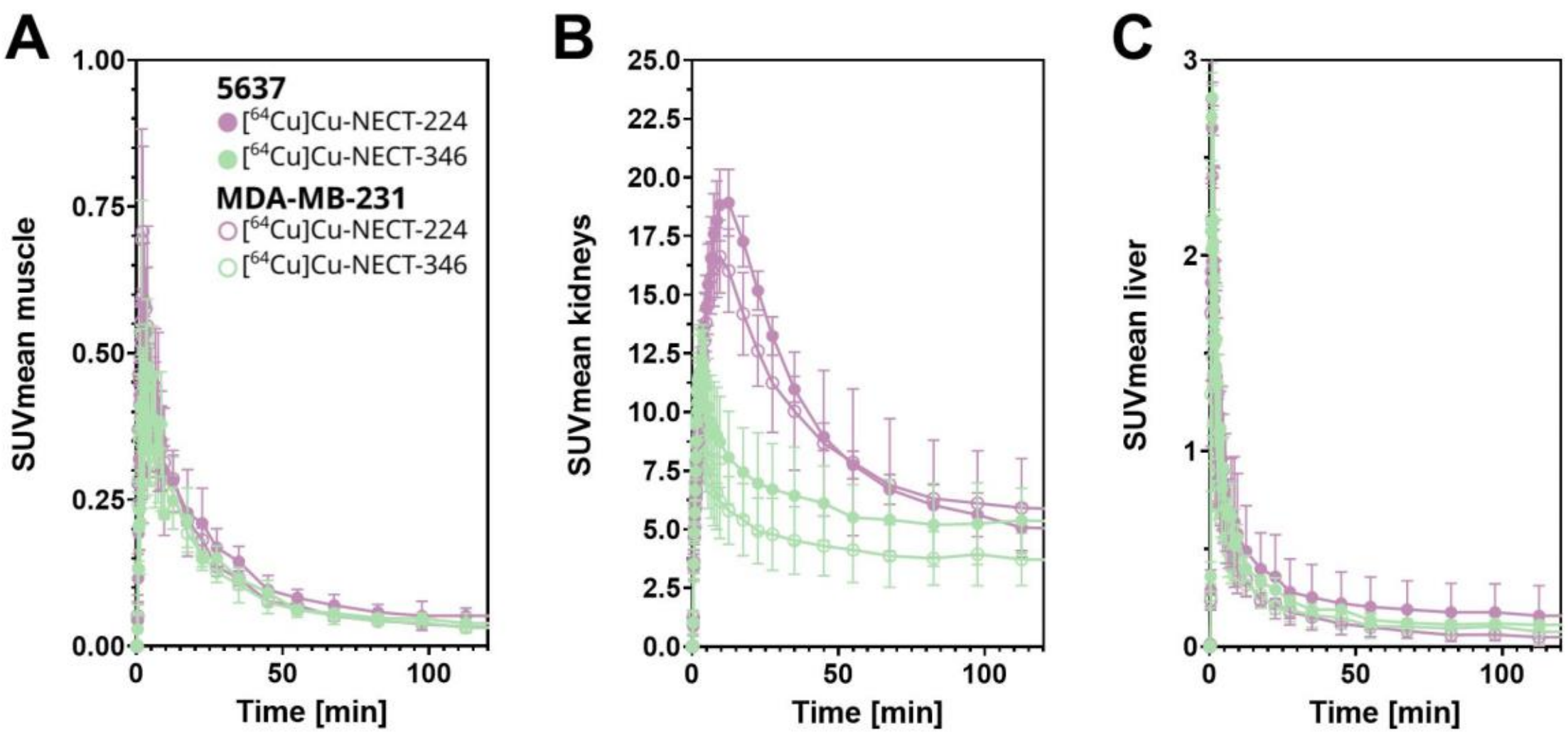

Figure S8: TACs for normal organs in HT-1376 tumor bearing mice obtained for the ^{61}Cu-labeled ligands

Time-activity curves (SUVmean, decay-corrected, as a function of time up to 2 h) of the muscle (**A**), kidney (**B**), and liver (**C**) obtained from quantitative analysis of PET images are depicted for the ligands **[^{61}Cu]Cu-NECT-224** and **[^{61}Cu]Cu-NECT-346**. Color coding according to Figure 5 in the main article.

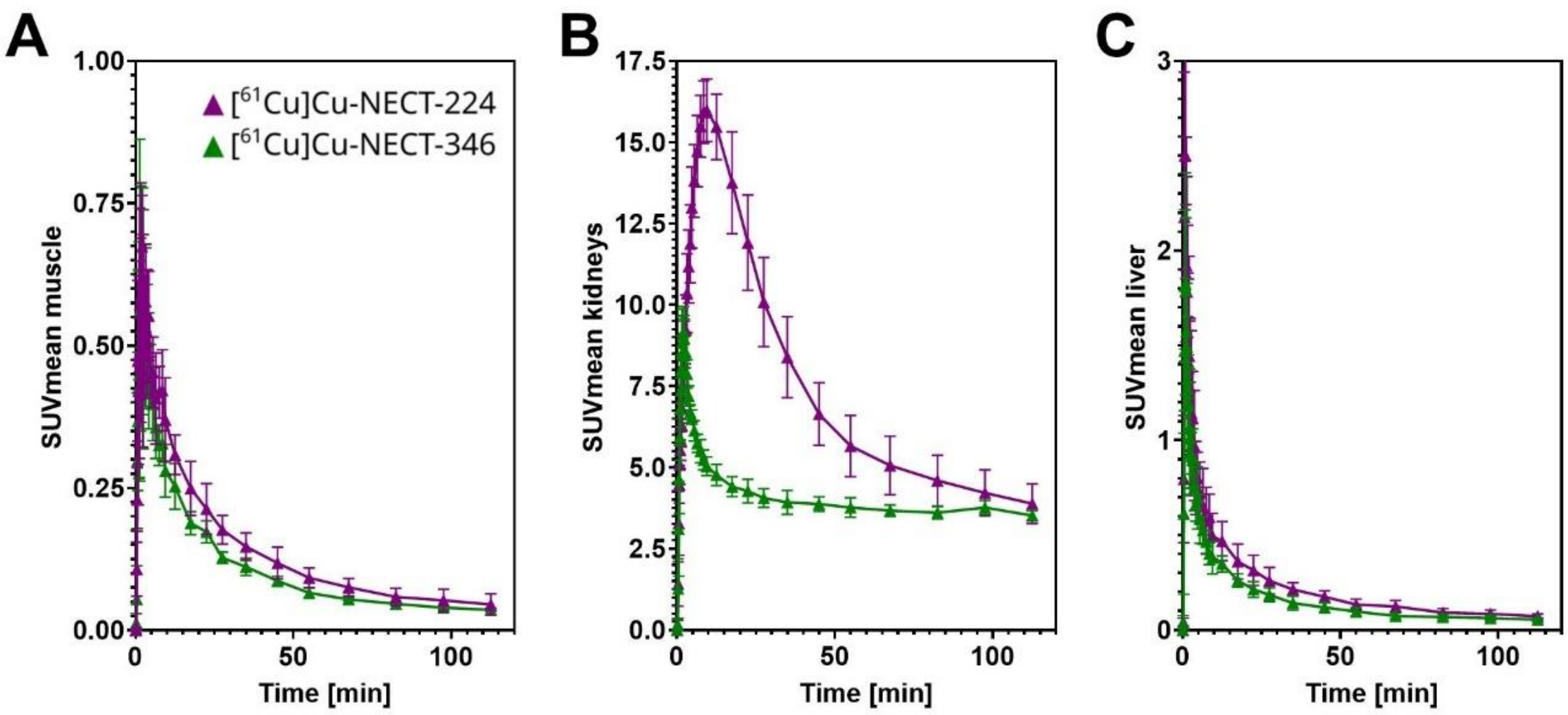

Figure S9: PET images and image-derived uptake values of [^{64}Cu]Cu-NECT-224 with and without co-administration of ADAM10/17 inhibitor INCB3619

A) PET/CT images at 1-2 h after intravenous injection of **[^{64}Cu]Cu-NECT-224** in HT-1376 tumor-bearing mice with and without **INCB3619**. 6-9 MBq/animal (0.3-0.45 nmol/animal) were injected. Images are presented as maximum intensity projections with a common scale. Anatomical positions of the tumor (tu), kidney (ki), and urinary bladder (ub) are shown. **B**) Time-activity curves (SUVmean, decay-corrected, as a function of time up to 2 h) for the blood content of the heart and tumor (**C**) obtained from quantitative analysis of PET images are depicted. **D/E**) Time-resolved SUVmean ratios up to 2 h p.i. including tumor-to-muscle (**D**) and tumor-to-heart (**E**). **F**) Time-resolved body retention in % of the initial dose up to 2 h p.i.. Data points in **C-G** represent mean values (±SD) measured in groups of 4 tumor-bearing mice.

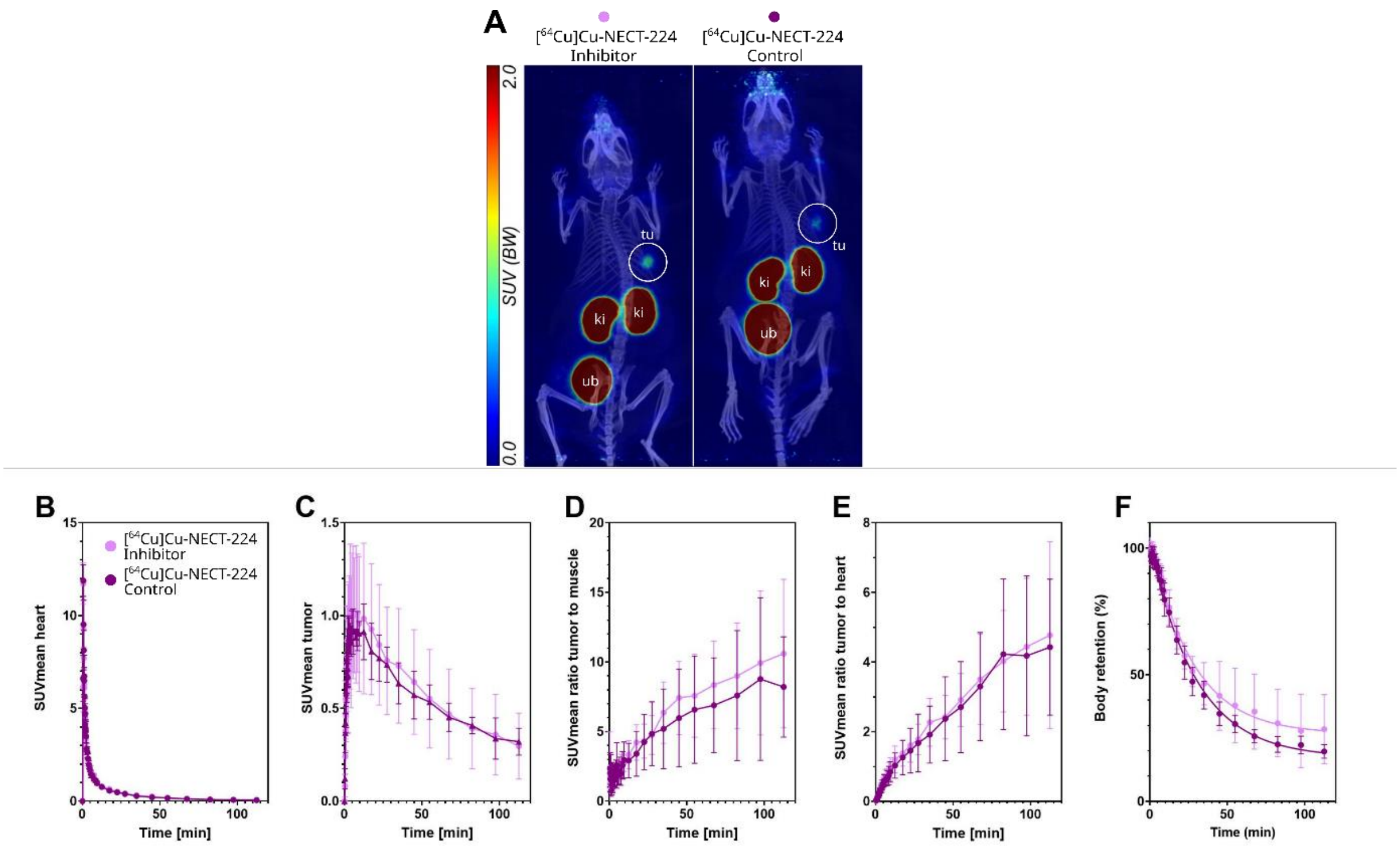

Figure S10: TACs for normal organs in HT-1376 tumor bearing mice of [^{64}Cu]Cu-NECT-224 with and without inhibitor INCB3619

Time-activity curves (SUVmean, decay-corrected, as a function of time up to 2 h) of the muscle (**A**), kidney (**B**), and liver (**C**) obtained from quantitative analysis of PET images are depicted for the ligand **[^{64}Cu]Cu-NECT-224** with and without administration of Inhibitor INCB3619.

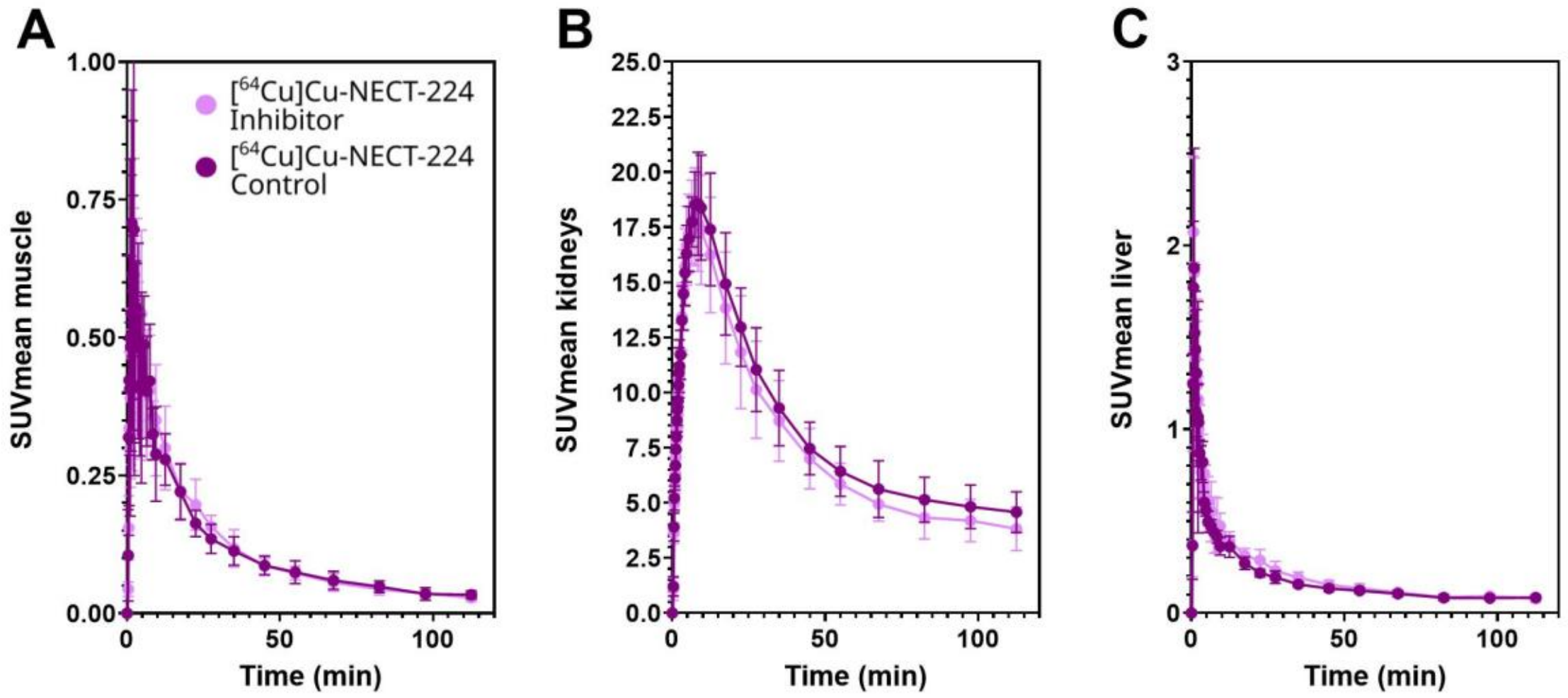

Figure S11: TACs for normal organs in MDA-MB-468 tumor bearing mice

Time-activity curves (SUVmean, decay-corrected, as a function of time up to 2 h) of the muscle (**A/D**), kidney (**B/E**), and liver (**C/F**) obtained from quantitative analysis of PET images are depicted for the ^{64}Cu-labeled (**A**-**C**) and ^{68}Ga-labeled ligands (**E-F**). Color coding according to Figure 5 in the main article.

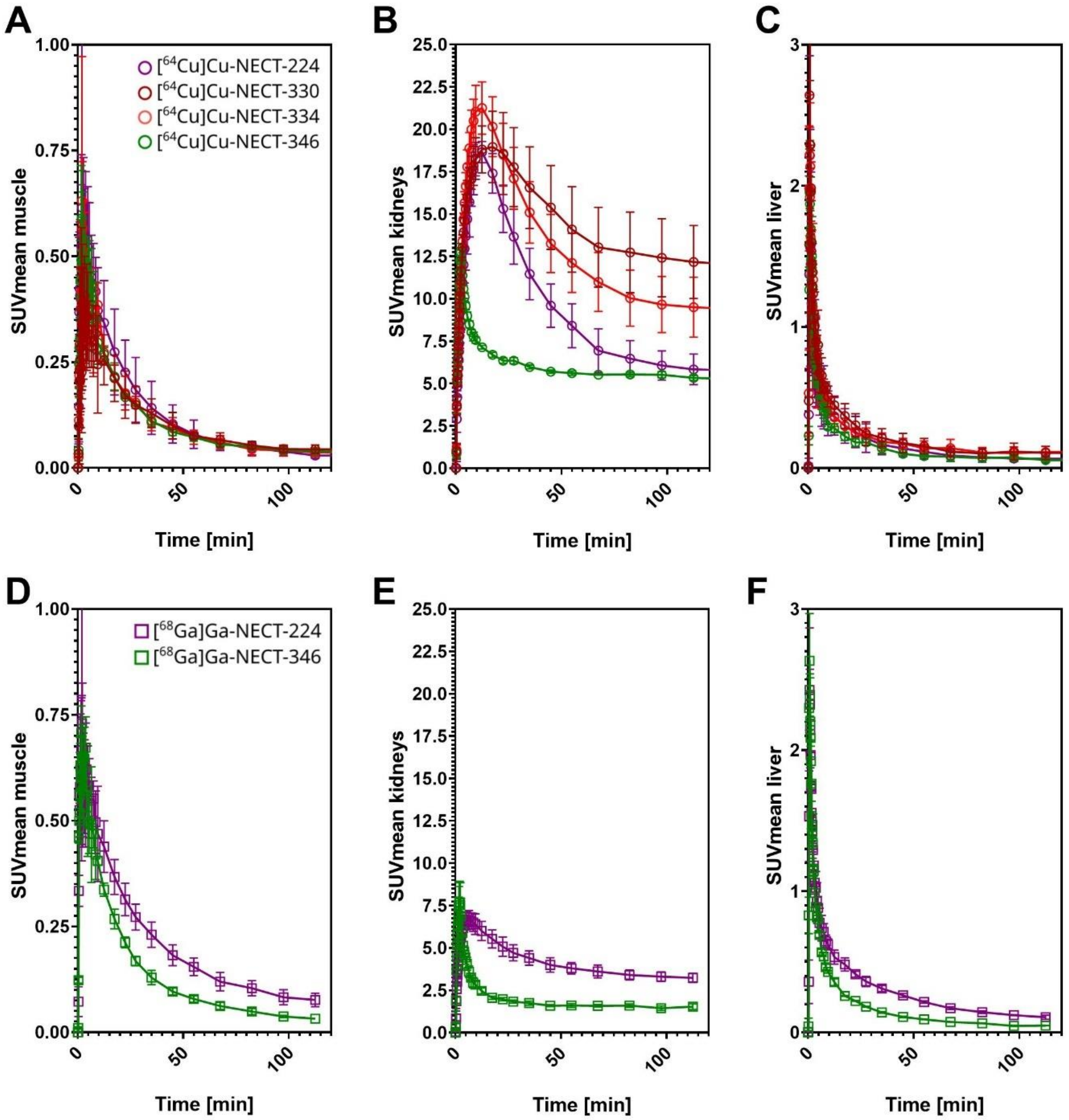

Figure S12: Insights into activity uptake in kidneys from first-in-human application of [^{64}Cu]Cu-NECT-224

Maximum intensity projection (MIP) scaled to a maximum standardized uptake value (SUVmax) of 22 acquired in the anterior view. Imaging was performed at 67 min (**left**) and 16.5 h (**right**) after intravenous injection of 251 MBq of **[^{64}Cu]Cu-NECT-224**.

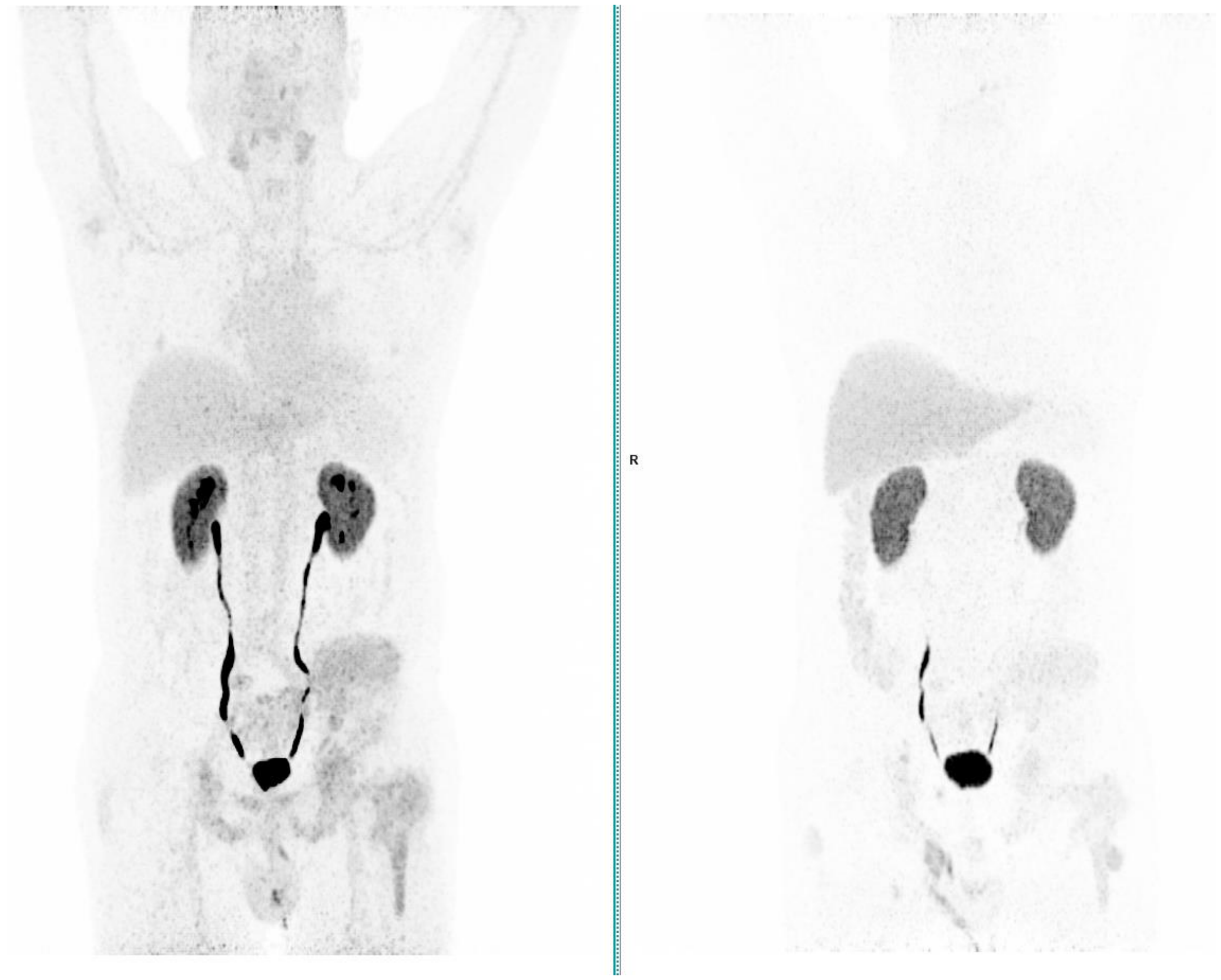

Figure S13: Exemplary TAC for the submandibularis in MDA-MB-468 tumor bearing mice of [^{64}Cu]Cu-NECT-224

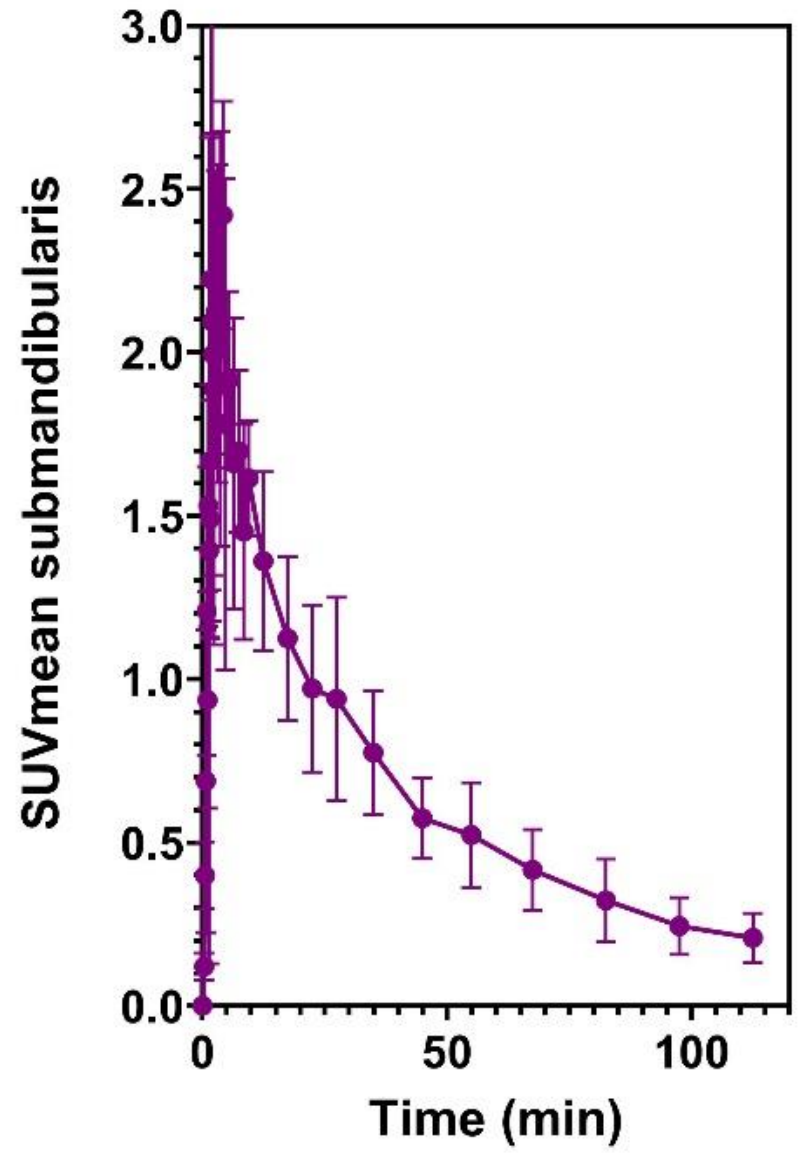

Time-activity curves (SUVmean, decay-corrected, as a function of time up to 2 h) of the submandibularis obtained from quantitative analysis of PET images are depicted for the ligand **[^{64}Cu]Cu-NECT-224**.

Figure S14: Regions of interest (ROIs) generated in Rover for extraction of tissue-specific uptake values from PET images

Exemplary data showing position (red crosshairs) and shape (black-yellow-white overlays) of the ROIs in orthogonal PET image slices of an HT-1376 tumor-bearing mouse including all signals from a full dynamic 2-hour-scan following **[^{64}Cu]Cu-NECT-224** injection; percentages refer to tissue-specific intensity thresholds (% of image's maximum voxel intensity) used for including voxels into the ROI.

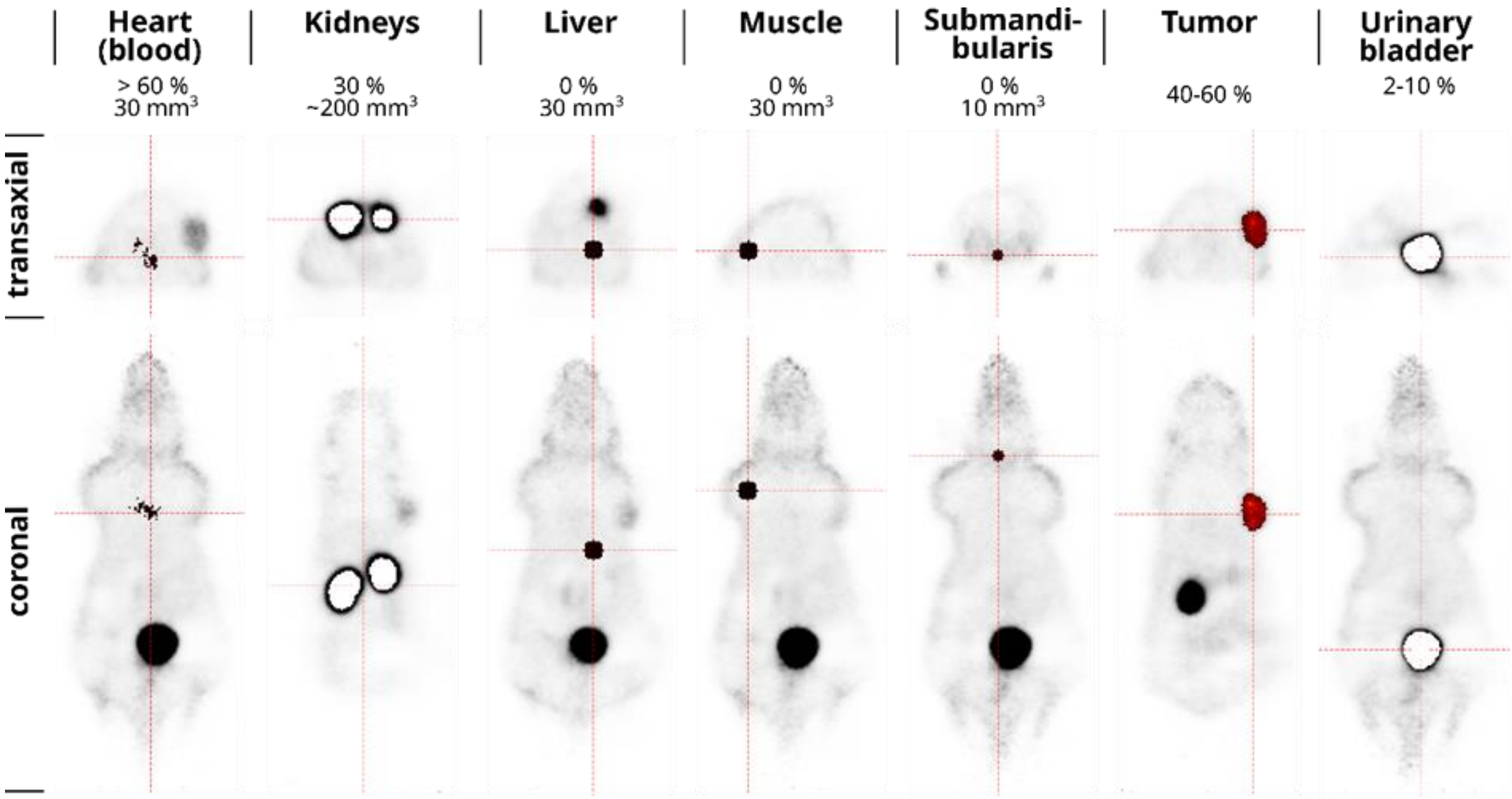

Scheme S1: General synthetic route to the Nectin-4 ligands

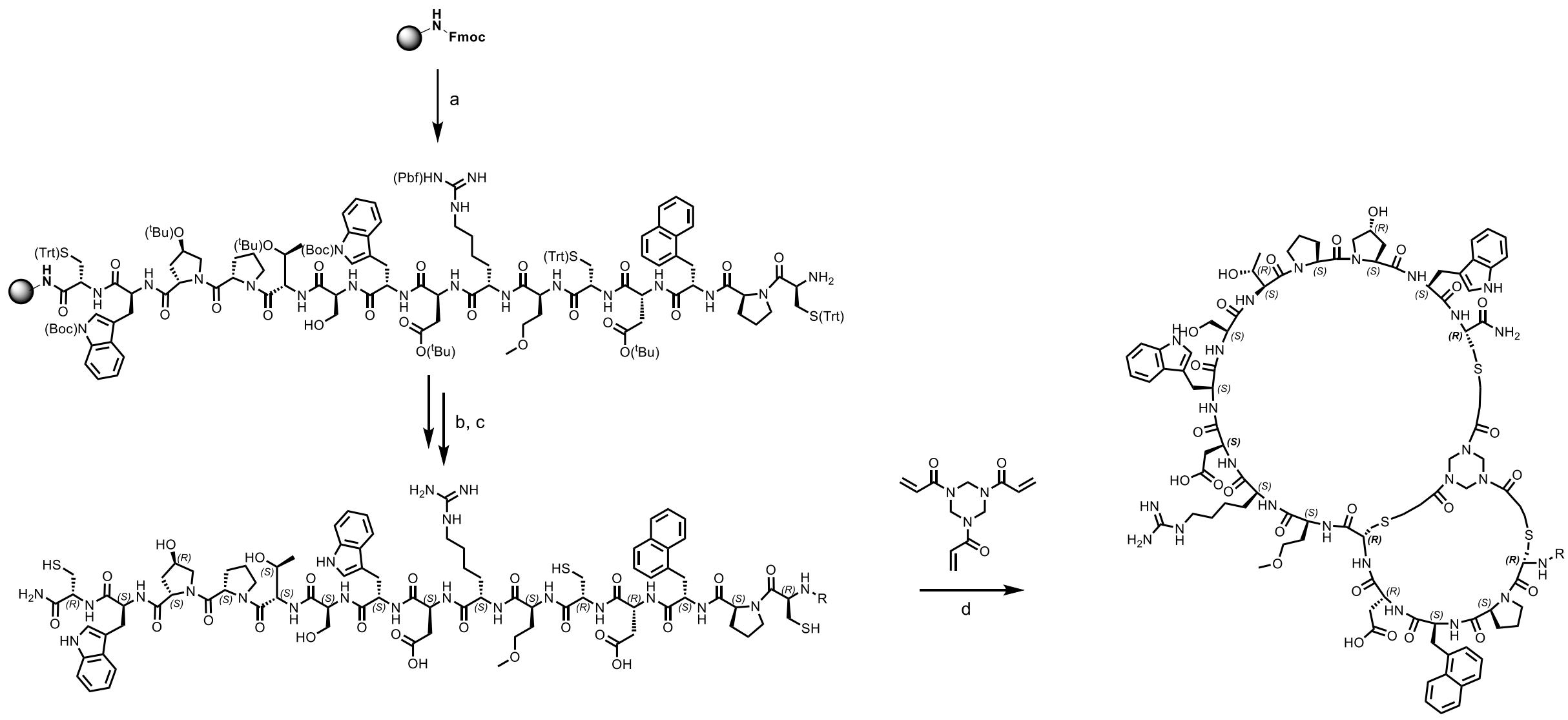

a) Fmoc/*t*Bu-based solid-phase peptide synthesis using an automated microwave peptide synthesizer (Biotage Initiator+ Alstra) with standard protocols for coupling (HATU/DIPEA) and Fmoc removal (20% piperidine in DMF); **b₁**) Fmoc-Linker-OH, HATU, DIPEA, DMF **b₂**) *R*-NODAGA(^{t}Bu)$_3$, HATU, DIPEA, DMF, 18 **c**) TFA/H_2O/TIPS/Anisol (92.5:2.5:2.5:2.5, v/v/v/v), 40°C, 1 h; **d**) 1,3,5-Triacryloylhexahydro-1,3,5-triazine (TATA), NH_3, 1 h.

Table S1: Analytical data of the bicyclic peptides

compound	Chemical formula	m/z calculated for $[M+2H]^{2+}$	m/z found[a] for $[M+2H]^{2+}$	Purity (%)[b]
**^{nat}Cu-NECT-224**	$C_{113}H_{151}N_{27}O_{33}S_3Cu$	1288.4799	1288.4791	> 98*
**^{nat}Ga-NECT-224**	$C_{113}H_{151}N_{27}O_{33}S_3Ga$	1290.9739	1290.9735	> 98*
**NECT-330**	$C_{119}H_{164}N_{28}O_{36}S_3$	1329.5585	1329.5584	> 95
**NECT-334**	$C_{131}H_{188}N_{28}O_{42}S_3$	1461.6372	1461.6361	> 99
**NECT-346**	$C_{146}H_{208}N_{38}O_{44}S_3$	1647.7257	1647.7245	> 95
**NECT-416**	$C_{98}H_{130}N_{24}O_{26}S_3$	1078.9464	1078.9464	> 97

[a]High-resolution mass spectra using electrospray ionization were recorded.

[b]Purity was determined by analytical RP-HPLC and is given for 230 nm.

*Purity based on NECT-224

Table S2: SUVmean and SUVmean ratios for the ^{64}Cu- and ^{68}Ga-labeled ligands in a HT-1376 tumor xenograft model

Data shown are mean values (±SD). Results of ordinary one-way ANOVA are indicated for the tumor-to-heart and tumor-to-muscle ratios at 1-2 h post injection (ns - non-specific, * - p ≤ 0.05, ** - p ≤ 0.01, *** - p ≤ 0.001). The ^{64}Cu-labeled ligands were statistically compared to **[^{64}Cu]Cu-NECT-224** and the ^{68}Ga-labeled ligands to **[^{68}Ga]Ga-NECT-224**.

	Time p.i.	**[^{64}Cu]Cu-NECT-224**	**[^{64}Cu]Cu-NECT-330**	**[^{64}Cu]Cu-NECT-334**	**[^{64}Cu]Cu-NECT-346**	**[^{68}Ga]Ga-NECT-224**	**[^{68}Ga]Ga-NECT-330**	**[^{68}Ga]Ga-NECT-334**	**[^{68}Ga]Ga-NECT-346**
**tumor SUVmean**	22.5 min[a]/ 5.5 min[b] (Max)	1.04 (±0.31)[a]	1.00 (±0.13)[a]	1.08 (±0.10)[a]	0.93 (±0.09)[a]	0.63 (±0.02)[b]	0.78 (±0.06)[b]	0.66 (±0.20)[b]	0.98 (±0.29)[b]
	1-2 h	0.76 (±0.29)	0.70 (±0.09)	0.75 (±0.15)	0.79 (±0.20)	0.35 (±0.09)	0.43 (±0.07)	0.41 (±0.12)	0.48 (±0.16)
**SUVmean ratios**									
**tumor to muscle**	9.5 min	2.06 (±0.46)	3.53 (±0.32)	3.95 (±0.72)	2.51 (±0.46)	1.12 (±0.11)	1.47 (±0.21)	1.75 (±0.30)	2.01 (±0.64)
	35 min	4.04 (±0.74)	6.46 (±1.50)	11.03 (±2.36)	6.29 (±1.26)	1.49 (±0.17)	2.70 (±0.29)	3.32 (±0.40)	3.85 (±1.40)
	1-2 h	7.40 (±1.29)	12.69*** (±4.36)	20.83*** (±7.83)	15.47*** (±7.16)	2.21 (±0.36)	5.26* (±0.82)	7.34*** (±1.36)	8.09*** (±3.79)
**tumor to heart**	9.5 min	0.73 (±0.18)	0.75 (±0.15)	1.11 (±0.12)	0.73 (±0.06)	0.45 (±0.07)	0.63 (±0.11)	0.59 (±0.14)	0.80 (±0.24)
	35 min	1.71 (±0.45)	1.89 (±0.31)	4.15 (±0.35)	2.49 (±0.27)	0.76 (±0.13)	1.28 (±0.16)	1.54 (±0.35)	1.79 (±0.49)
	1-2 h	3.60 (±0.89)	5.25[ns] (±1.41)	11.17*** (±2.03)	8.69*** (±3.75)	1.23 (±0.26)	3.05* (±0.72)	3.77*** (±1.16)	3.97*** (±1.24)
**tumor to kidney**	9.5 min	0.07 (±0.02)	0.06 (±0.01)	0.06 (±0.01)	0.08 (±0.02)	0.08 (±0.01)	0.11 (±0.02)	0.15 (±0.04)	0.24 (±0.05)
	35 min	0.06 (±0.02)	0.06 (±0.01)	0.07 (±0.00)	0.11 (±0.04)	0.05 (±0.02)	0.14 (±0.01)	0.16 (±0.03)	0.25 (±0.06)
	1-2 h	0.07 (±0.03)	0.1 (±0.01)	0.07 (±0.01)	0.1 (±0.04)	0.06 (±0.01)	0.14 (±0.01)	0.14 (±0.04)	0.22 (±0.04)

Table S3: SUVmean and SUVmean ratios for the ^{64}Cu- and ^{68}Ga-labeled ligands in a MDA-MB-468 xenograft model

Data shown are mean values (±SD). Results of ordinary one-way ANOVA are indicated for the tumor-to-heart and tumor-to-muscle ratios at 1-2 h post injection (ns - non-specific, * - p ≤ 0.05, ** - p ≤ 0.01, *** - p ≤ 0.001). The ^{64}Cu-labeled ligands were statistically compared to **[^{64}Cu]Cu-NECT-224** and the ^{68}Ga-labeled ligands to **[^{68}Ga]Ga-NECT-224**.

	Time p.i.	**[^{64}Cu]Cu-NECT-224**	**[^{64}Cu]Cu-NECT-330**	**[^{64}Cu]Cu-NECT-334**	**[^{64}Cu]Cu-NECT-346**	**[^{68}Ga]Ga-NECT-224**	**[^{68}Ga]Ga -NECT-346**
**tumor SUVmean**	8 min (Max)	0.67 (±0.12)	0.77 (±0.03)	0.89 (±0.18)	0.68 (±0.02)	0.70 (0.07)	0.66 (0.03)
	1-2 h	0.25 (±0.10)	0.47 (±0.10)	0.48 (±0.14)	0.46 (±0.08)	2.16 (0.19)	1.45 (0.24)
**SUVmean ratios**							
**tumor to muscle**	9.5 min	1.82 (±0.52)	2.07 (±0.20)	4.05 (±1.03)	2.32 (±0.40)	1.61 (±0.30)	1.69 (±0.09)
	35 min	3.46 (±1.02)	6.02 (±.51)	5.80 (±0.50)	5.46 (±0.76)	3.10 (±0.26)	3.86 (±0.80)
	1-2 h	5.98 (±1.92)	10.57** (±1.29)	9.30 ^{ns} (±1.66)	9.95* (±1.66)	5.84 (±0.65)	8.54^{ns} (±1.86)
**tumor to heart**	9.5 min	0.60 (±0.07)	0.73 (±0.07)	0.73 (±0.06)	0.67 (±0.08)	0.67 (±0.05)	0.67 (±0.05)
	35 min	1.27 (±0.28)	2.16 (±0.28)	1.96 (±0.19)	2.22 (±0.28)	1.54 (±0.07)	1.54 0.07)
	1-2 h	2.51 (±0.74)	5.26** (±1.33)	4.23 ^{ns} (±0.93)	6.23*** (±1.45)	3.36 (±0.36)	4.16^{ns} (±0.41)
**tumor to kidney**	9.5 min	0.04 (±0.01)	0.04 (±0.00)	0.05 (±0.01)	0.09 (±0.01)	0.11 (±0.01)	0.24 (±0.01)
	35 min	0.04 (±0.01)	0.04 (±0.00)	0.05 (±0.01)	0.10 (±0.01)	0.16 (±0.01)	0.28 (±0.02)
	1-2 h	0.04 (±0.01)	0.05 (±0.00)	0.04 (±0.01)	0.08 (±0.01)	0.16 (±0.01)	0.23 (±0.02)

Table S4: SUVmean and SUVmean ratios for [^{64}Cu]Cu-NECT-224 and [^{64}Cu]Cu-NECT-346 in 5637 and MDA-MB-231 tumor xenograft models

Data shown are mean values (±SD).

	Time p.i.	5637 tumor-bearing mice		MDA-MB-231 tumor-bearing mice	
		**[^{64}Cu]Cu-NECT-224**	**[^{64}Cu]Cu-NECT-346**	**[^{64}Cu]Cu-NECT-224**	**[^{64}Cu]Cu-NECT-346**
**tumor SUVmean**	22.5 min[a]/8.5 min[b] (Max)	0.55 (±0.06)[a]	0.99 (±0.12)[a]	0.57 (±0.01)[b]	0.43 (±0.07)[b]
	1-2 h	0.38 (±0.08)	0.80 (±0.13)	0.10 (±0.02)	0.13 (±0.03)
**SUVmean ratios**					
**tumor to muscle**	9.5 min	2.37 (±0.71)	4.33 (±0.036)	1.67 (±0.11)	1.71 (±0.46)
	35 min	3.94 (±0.86)	8.63 (±0.34)	2.16 (±0.11)	2.66 (±0.44)
	1-2 h	7.25 (±2.66)	17.20 (±2.90)	2.29 (±0.42)	3.29 (±0.52)
**tumor to heart**	9.5 min	0.54 (±0.03)	0.73 (±0.02)	0.53 (±0.04)	0.44 (±0.02)
	35 min	1.45 (±0.32)	2.68 (±0.35)	0.81 (±0.03)	1.02 (±0.05)
	1-2 h	3.39 (±1.01)	8.09 (±1.92)	1.11 (±0.21)	1.64 (±0.19)
**tumor to kidney**	9.5 min	0.03 (±0.00)	0.12 (±0.03)	0.03 (±0.00)	0.08 (±0.02)
	35 min	0.05 (±0.00)	0.17 (±0.06)	0.03 (±0.00)	0.06 (±0.01)
	1-2 h	0.07 (±0.01)	0.17 (±0.06)	0.02 (±0.00)	0.04 (±0.01)

Table S5: SUVmean and SUVmean ratios for [^{61}Cu]Cu-NECT-224 and [^{61}Cu]Cu-NECT-346 in HT-1376 tumor xenograft models

Data shown are mean values (±SD).

	Time p.i.	**[^{61}Cu]Cu-NECT-224**	**[^{61}Cu]Cu-NECT-346**
**tumor SUVmean**	7.5 min (Max)	1.30 (±0.21)	1.14 (±0.09)
	1-2 h	2.73 (±0.38)	2.97 (±0.12)
**SUVmean ratios**			
**tumor to muscle**	9.5 min	3.10 (±0.61)	3.43 (±0.74)
	35 min	7.11 (±1.16)	7.98 (±1.56)
	1-2 h	13.13 (±4.10)	15.95 (±3.74)
**tumor to heart**	9.5 min	1.03 (±0.11)	1.20 (±0.44)
	35 min	2.88 (±0.12)	3.65 (±0.93)
	1-2 h	6.69 (±0.59)	8.32 (±1.58)
**tumor to kidney**	9.5 min	0.07 (±0.01)	0.19 (±0.05)
	35 min	0.12 (±0.01)	0.23 (±0.05)
	1-2 h	0.15 (±0.01)	0.19 (±0.04)

Table S6: Blood kinetic parameters for the ^{64}Cu- and ^{61}Cu-labeled ligands in a HT-1376 tumor xenograft model

Parameters were derived from non-linear regression of the TAC for heart uptake according to the model of two-phase decay. Given are mean values and standard errors.

	[^{64}Cu]Cu-NECT-224	[^{64}Cu]Cu-NECT-330	[^{64}Cu]Cu-NECT-334	[^{64}Cu]Cu-NECT-346	[^{61}Cu]Cu-NECT-224	[^{61}Cu]Cu-NECT-346
**Percent fast [%]**	85.28 (±0.66)	88.42 (±0.89)	91.82 (±0.69)	92.97 (±0.88)	88.02 (0.84)	90.69 (0.87)
**Blood half life fast [min]**	1.30 (0.07)	1.81 (0.09)	1.75 (0.11)	1.40 (0.14)	1.57 (0.11)	1.72 (0.14)
**Blood half life slow [min]**	32.05 (0.85)	26.83 (0.98)	25.91 (1.21)	26.86 (1.70)	27.49 (1.10)	29.41 (1.72)

Table S7: Blood kinetic parameters for the ^{68}Ga-labeled ligands in a HT-1376 tumor xenograft model

Parameters were derived from non-linear regression of the TAC for heart uptake according to the model of two-phase decay. Given are mean values and standard errors.

	**[^{68}Ga]Ga-NECT-224**	**[^{68}Ga]Ga-NECT-330**	**[^{68}Ga]Ga-NECT-334**	**[^{68}Ga]Ga-NECT-346**
**Percent fast [%]**	78.47 (1.93)	81.81 (±1.03)	87.26 (1.03)	85.88 (0.74)
**Blood half life fast [min]**	1.36 (0.18)	1.51 (0.11)	1.60 (0.11)	1.48 (0.09)
**Blood half life slow [min]**	39.14 (2.43)	27.68 (0.85)	27.89 (1.05)	28.12 (0.83)

Compound characterization data

Bicyclic peptides

NECT-330

NECT-330 (2.1 mg, 6 %) was obtained as a white powder.

HPLC chromatogram

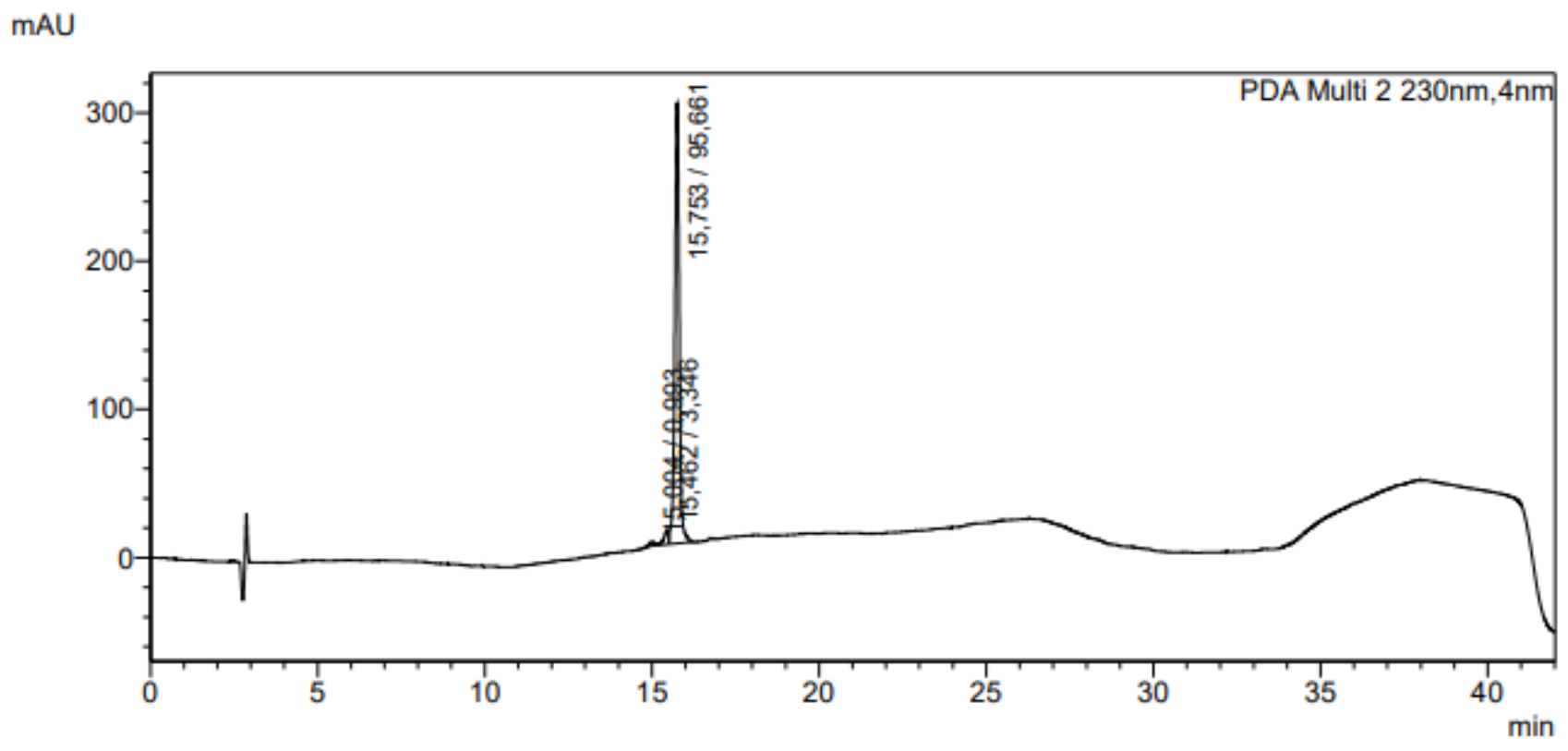

PDA Ch2 230nm

Peak#	Ret. Time	Area%
1	15,004	0,993
2	15,462	3,346
3	15,753	95,661
Total		100,000

HRMS spectrum (ESI+)

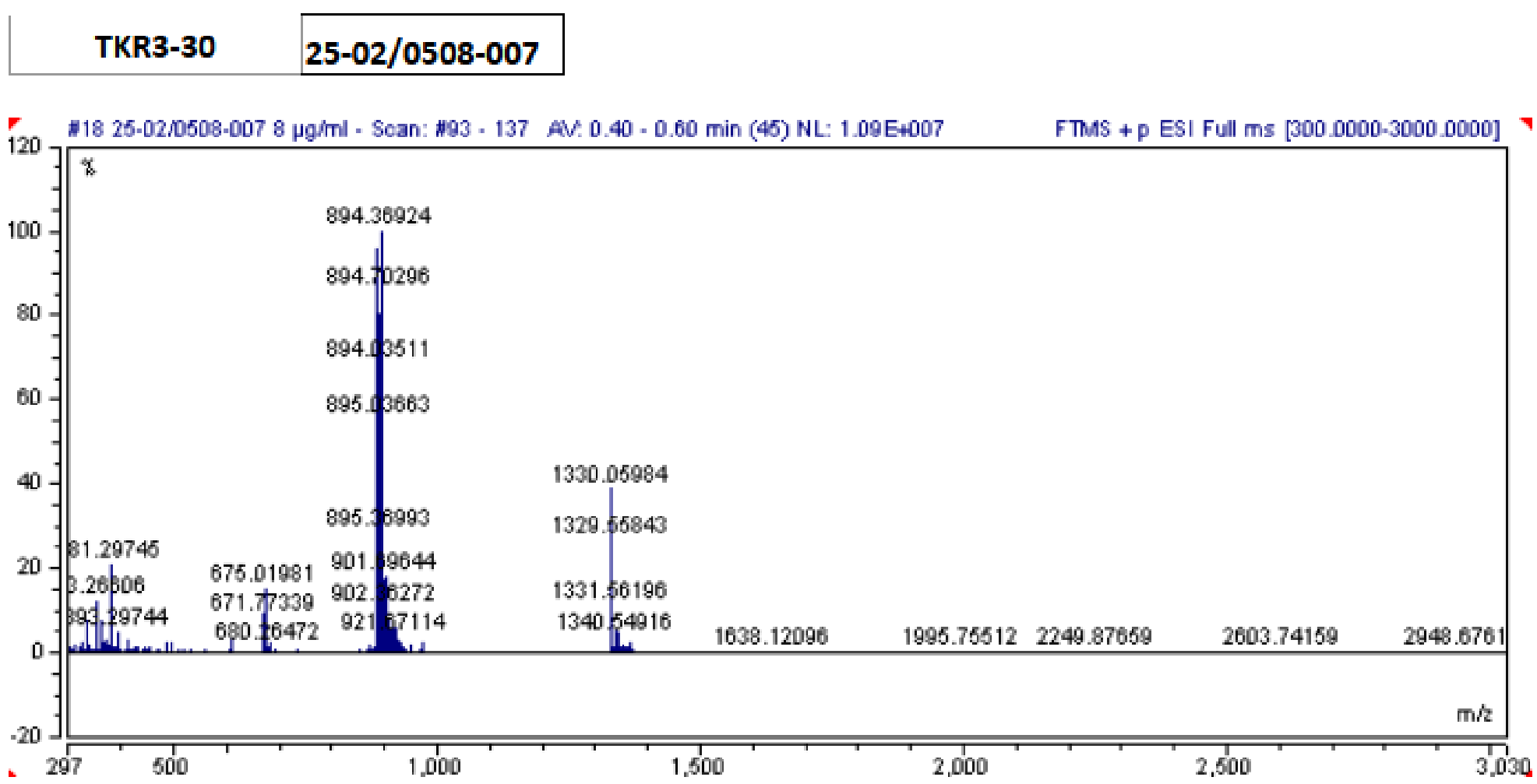

NECT-334

NECT-334 (1.8 mg, 5 %) was obtained as a white powder.

HPLC chromatogram

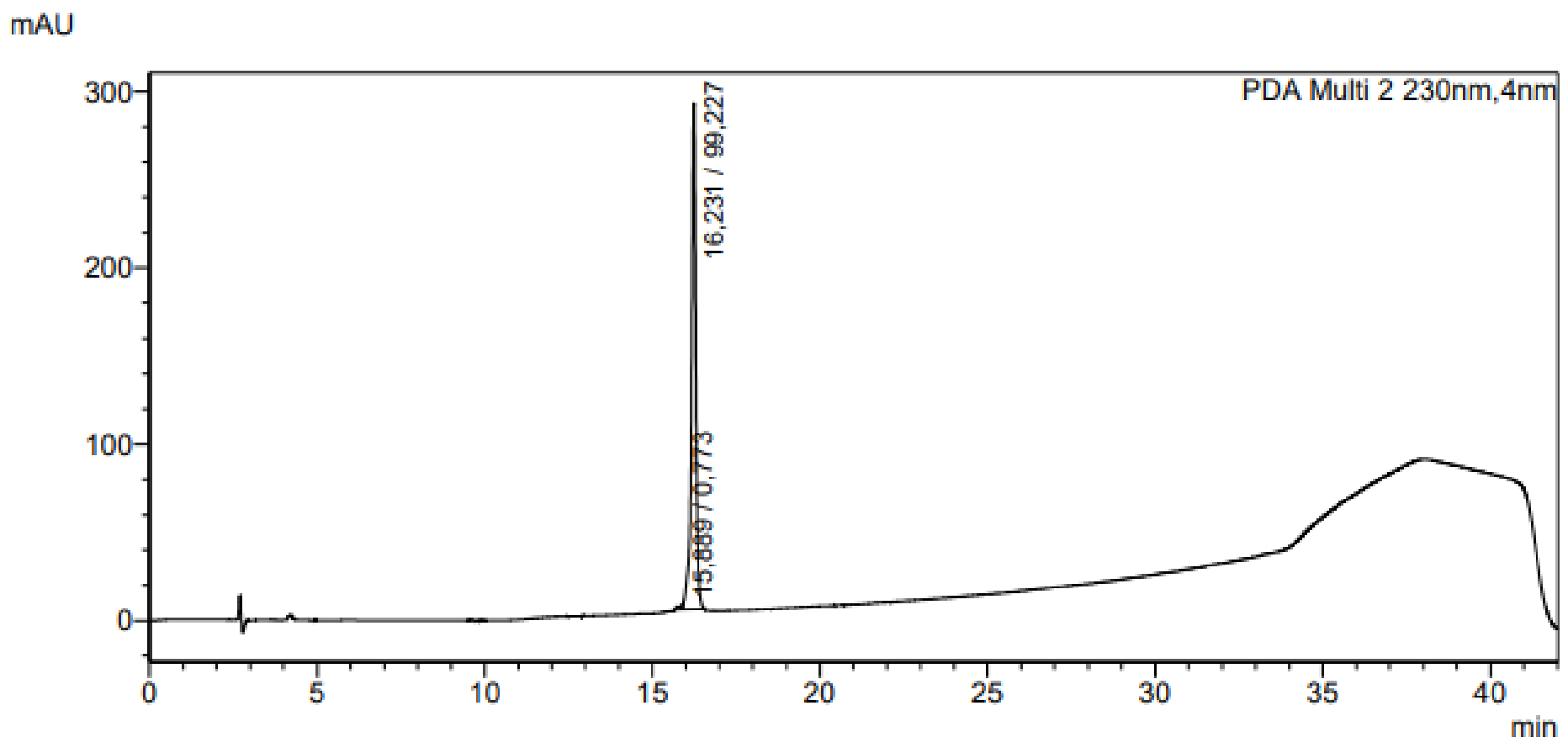

PDA Ch2 230nm

Peak#	Ret. Time	Area%
1	15,889	0,773
2	16,231	99,227
Total		100,000

HRMS spectrum (ESI+)

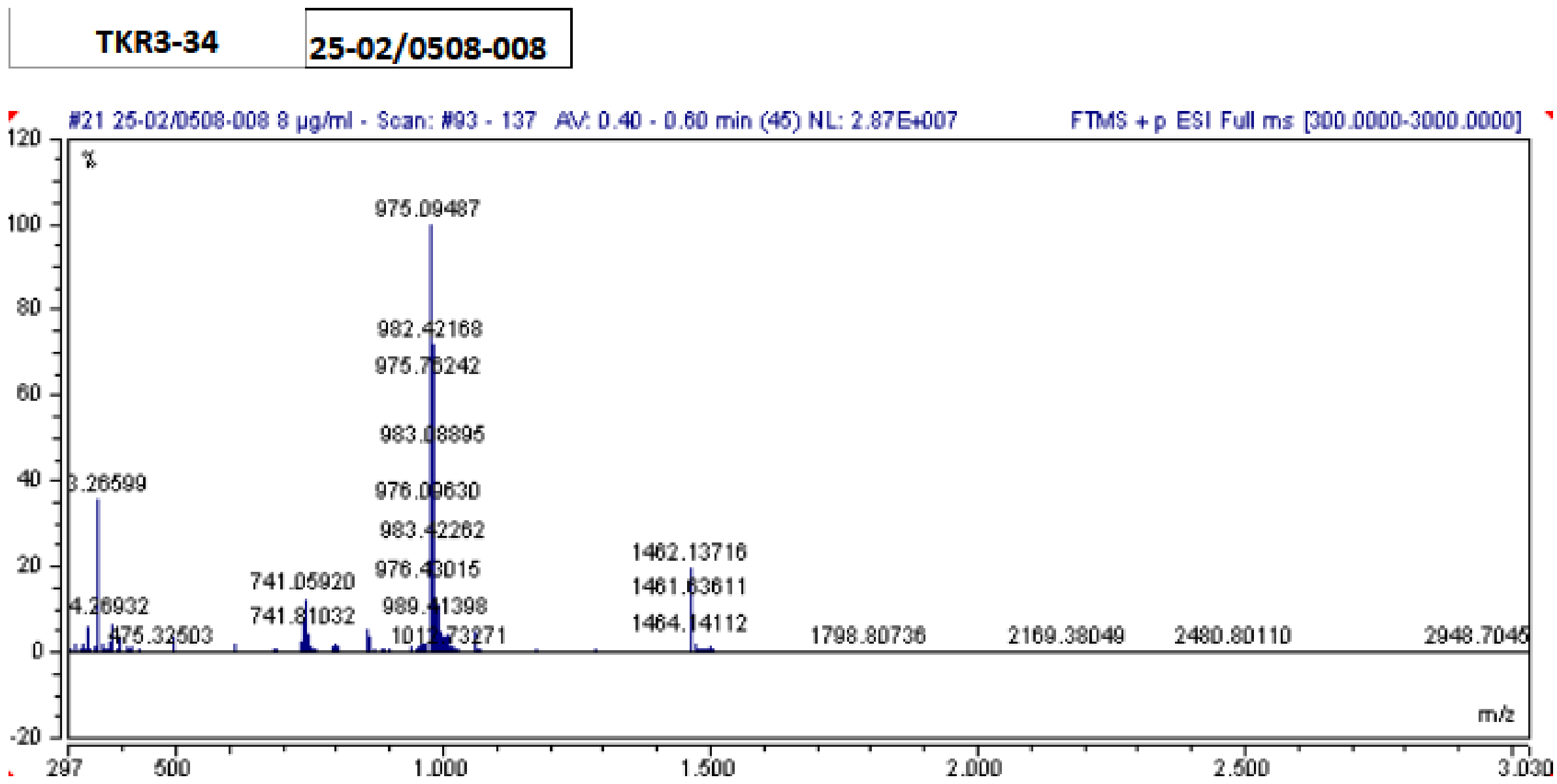

NECT-346

NECT-346 (2.8 mg, 6 %) was obtained as a white powder.

HPLC chromatogram

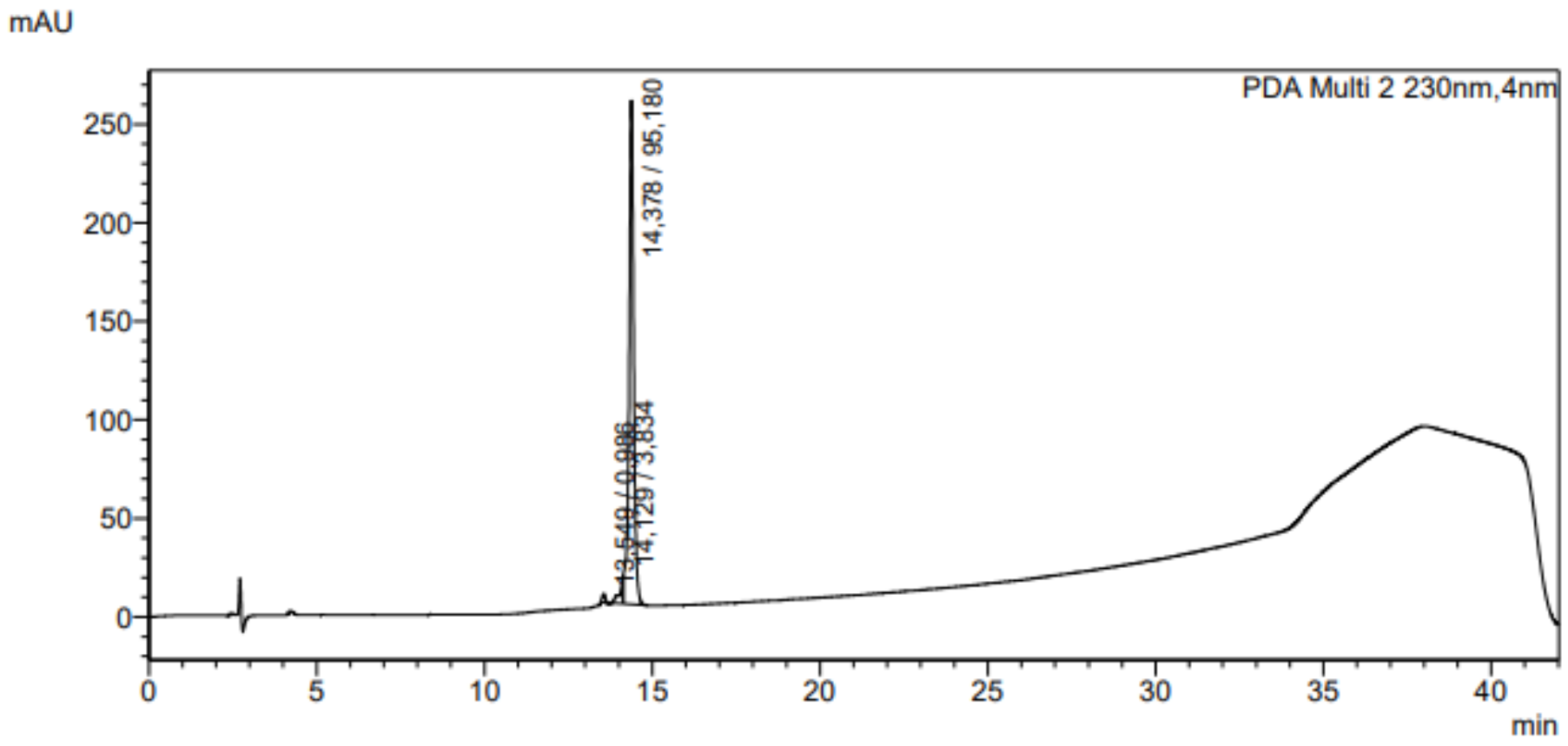

PDA Ch2 230nm			
Peak#	Ret. Time	Area	Area%
1	13,549	25527	0,986
2	14,129	99286	3,834
3	14,378	2464644	95,180
Total		2589456	100,000

HRMS spectrum (ESI+)

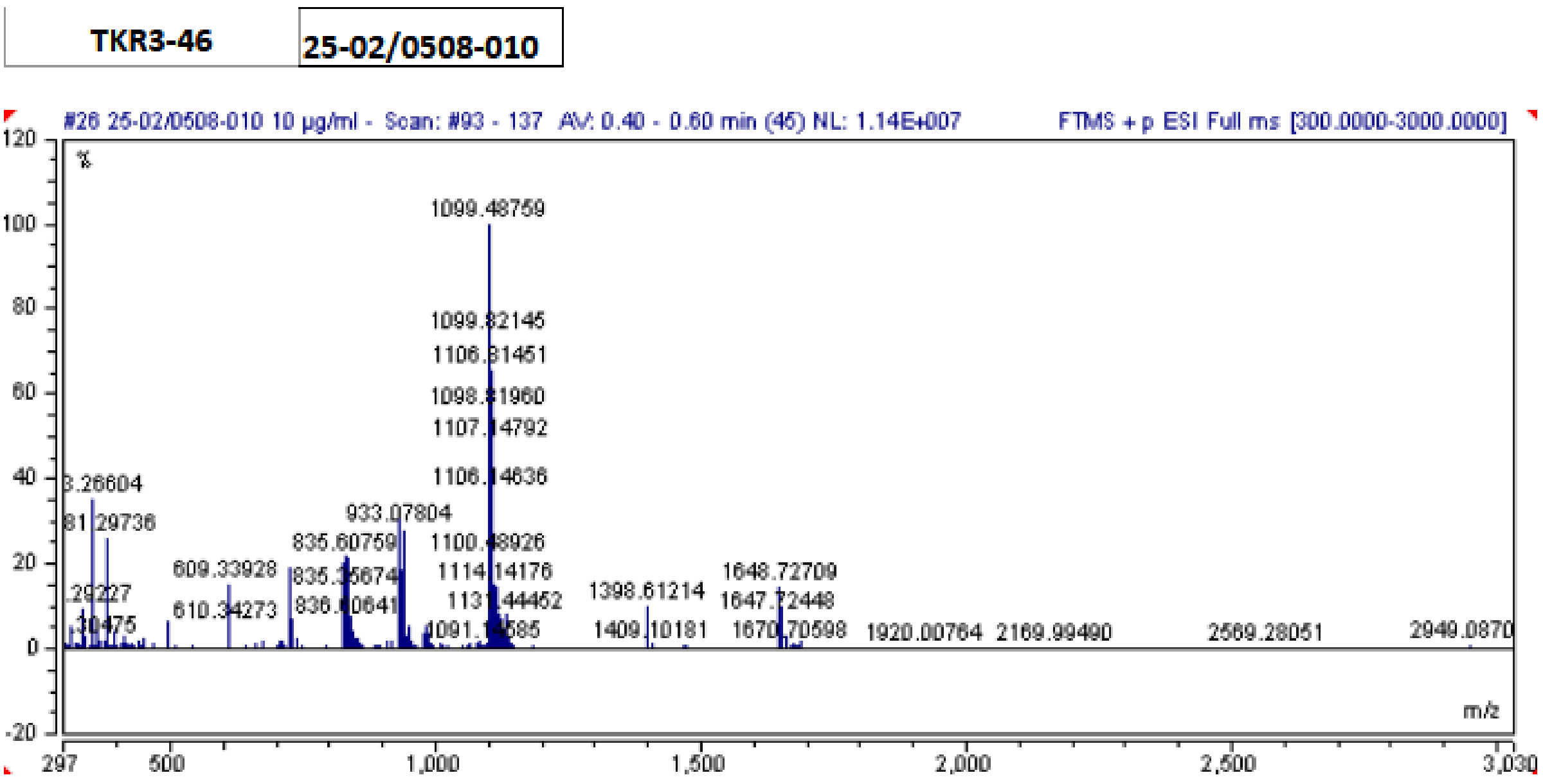

^{nat}Cu-NECT-224

^{nat}Cu-NECT-224 (0.2 mg, 95%) was obtained as a blue solid.

UPLC chromatogram (DMSO stock, 280 nm)

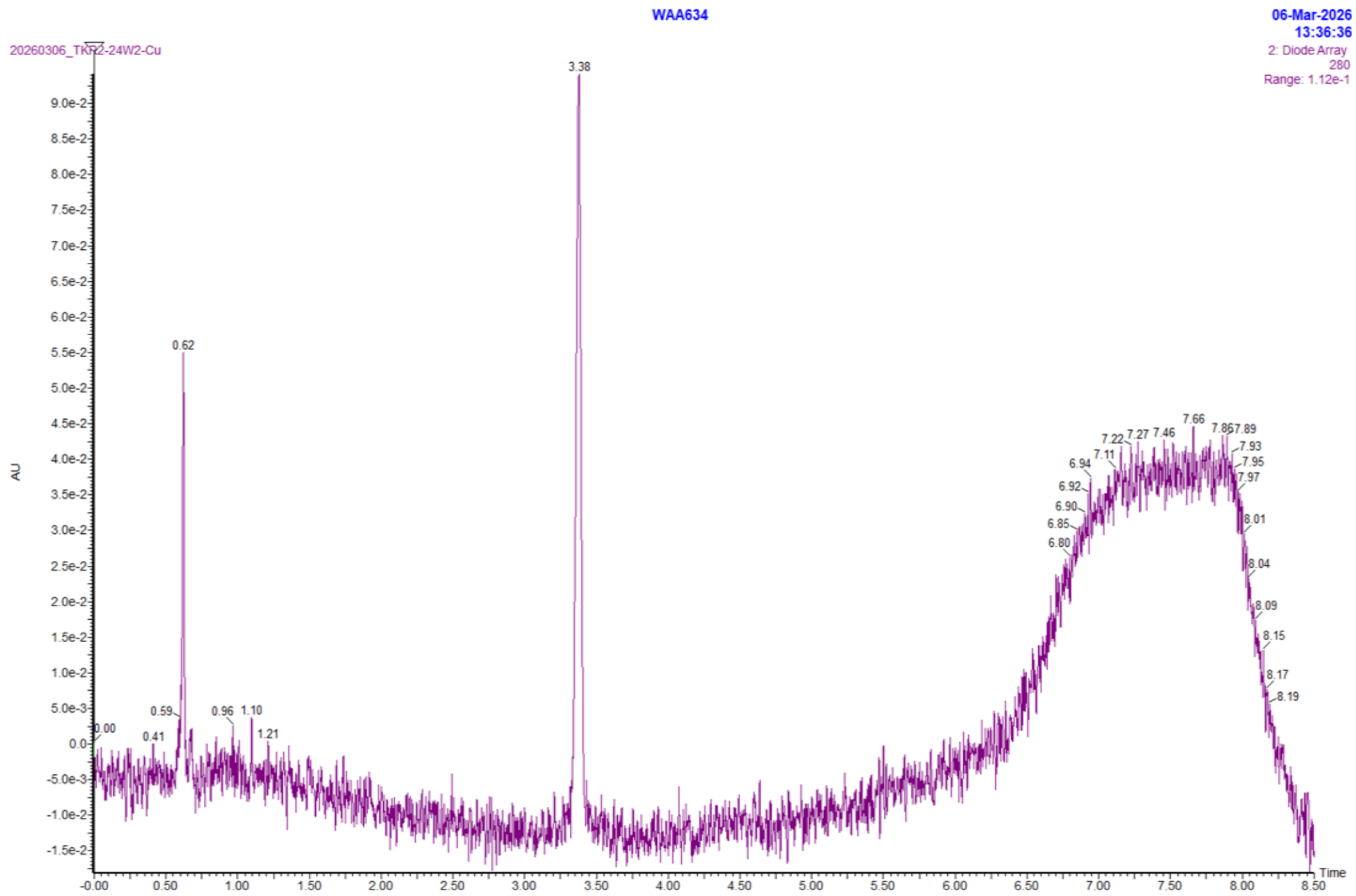

HRMS spectrum (ESI+)

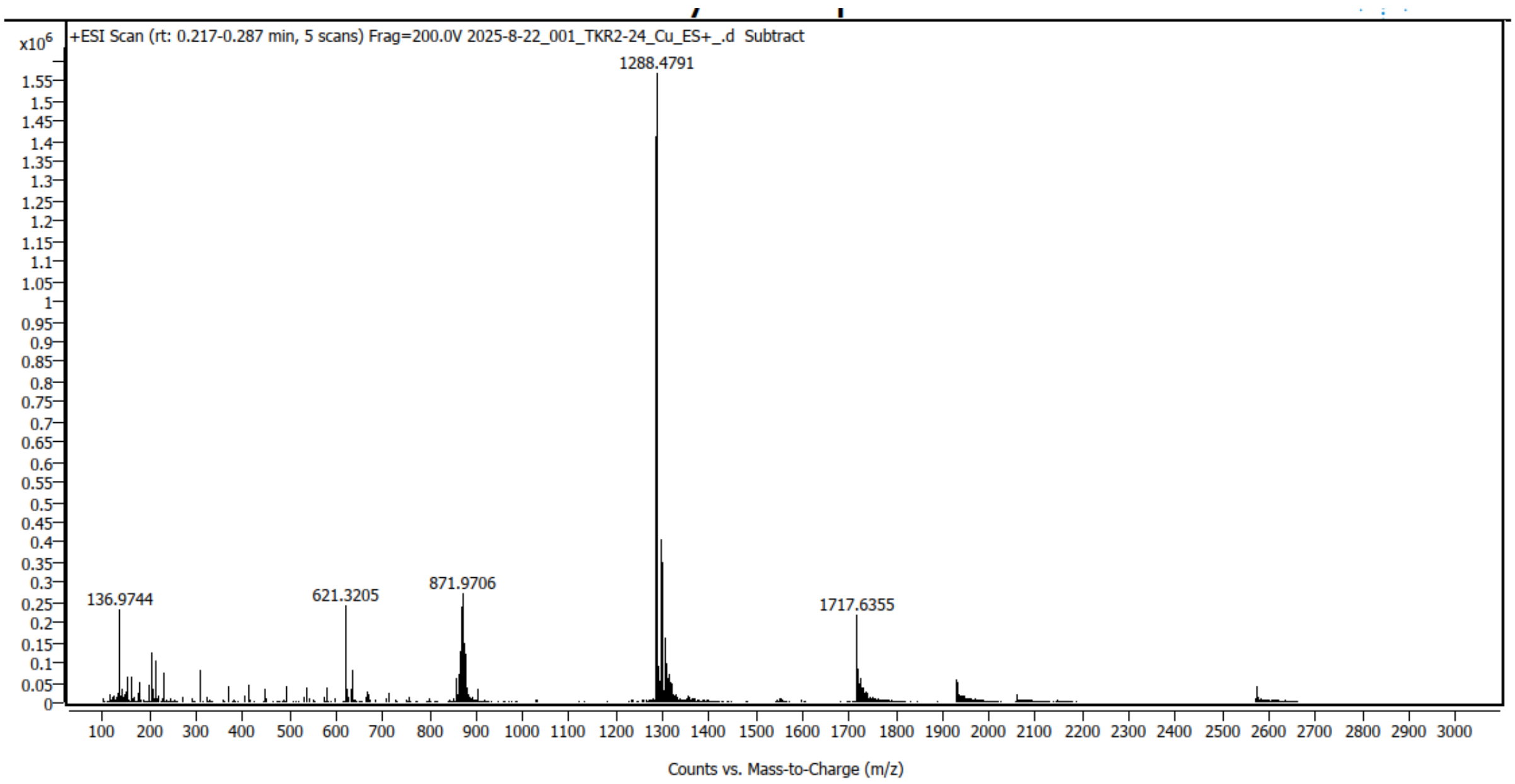

^{nat}Ga-NECT-224

^{nat}Ga-NECT-224 (0.2 mg, 95 %) was obtained as a white solid.

UPLC chromatogram (DMSO Stock, 280 nm)

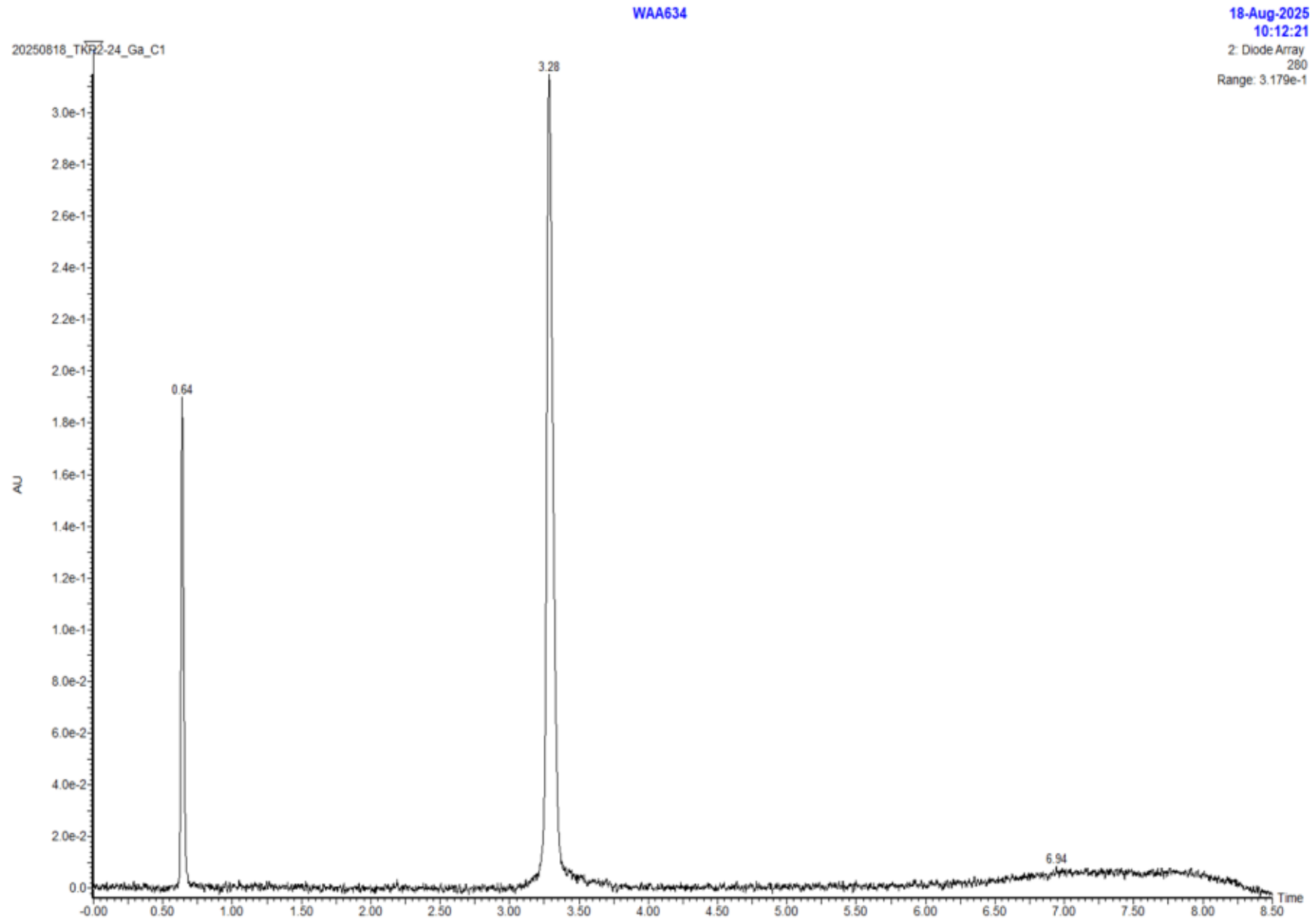

HRMS spectrum (ESI+)

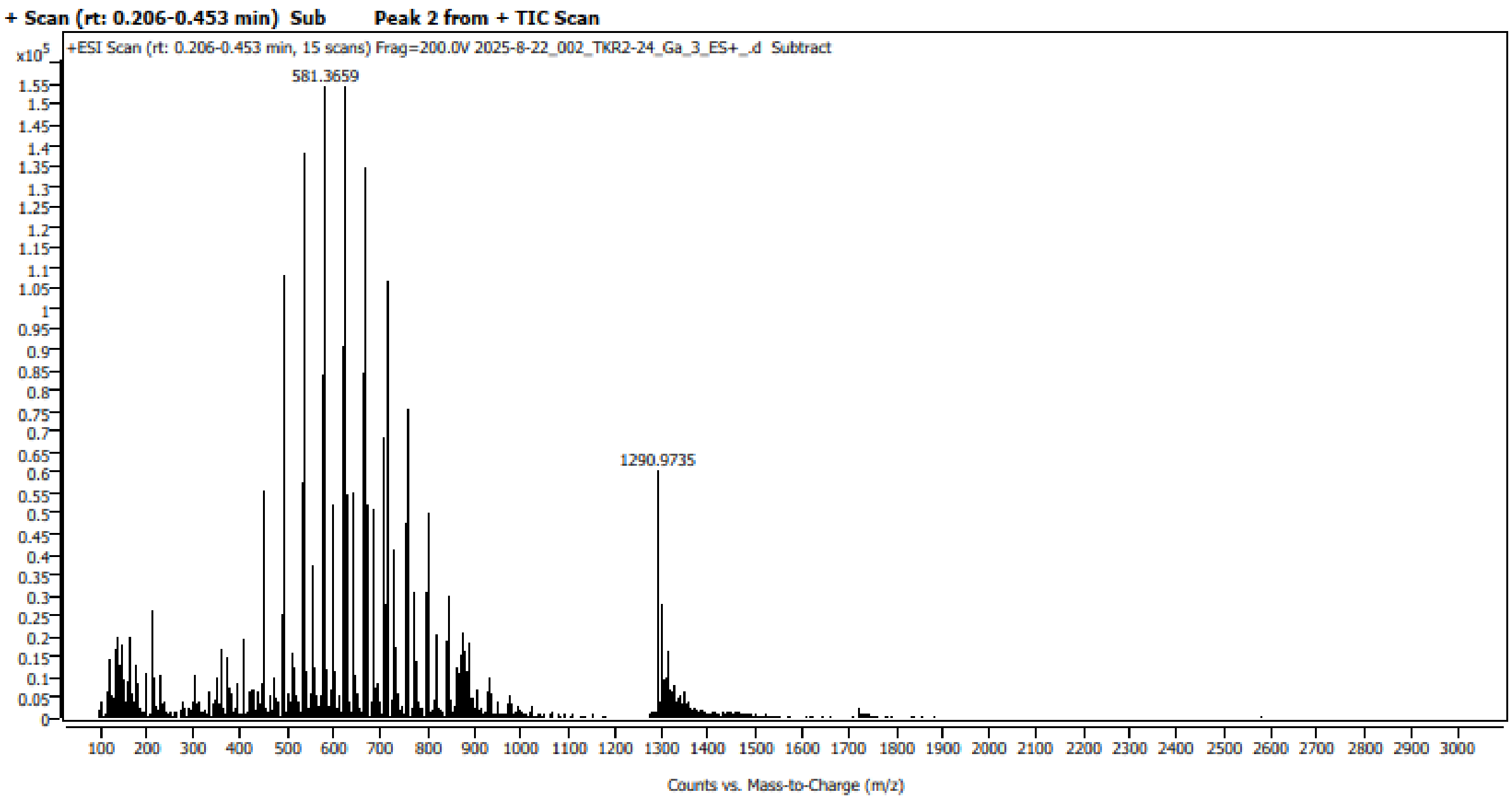

NECT-416

NECT-416 (2.8 mg, 10 %) was obtained as a white solid.

HPLC chromatogram

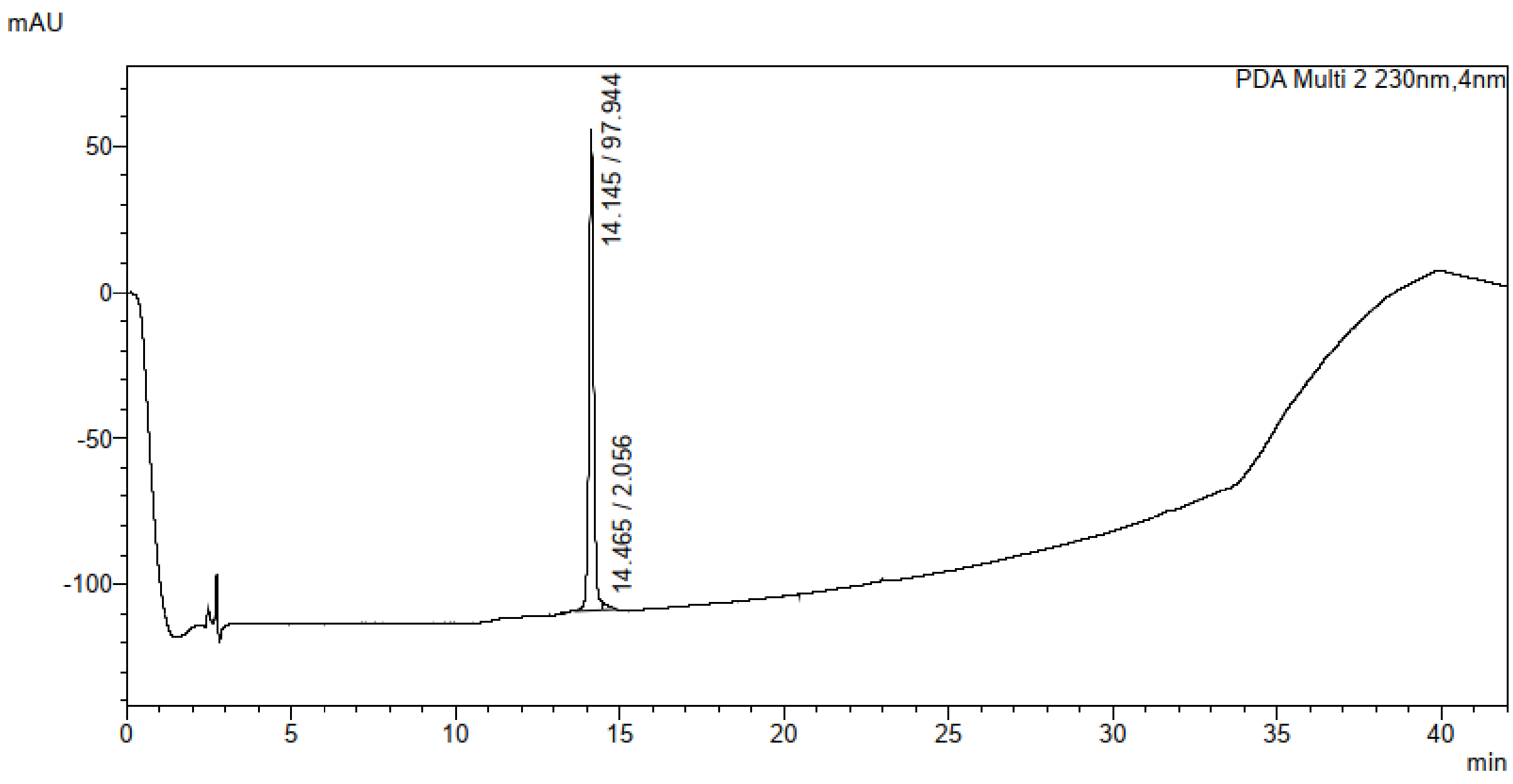

PDA Ch2 230nm

Peak#	Ret. Time	Area	Height	Conc.	Unit	Area%
1	14.145	1600123	164349	0.000		97.944
2	14.465	33594	2859	0.000		2.056
Total		1633717	167208			100.000

HRMS spectrum (ESI+)

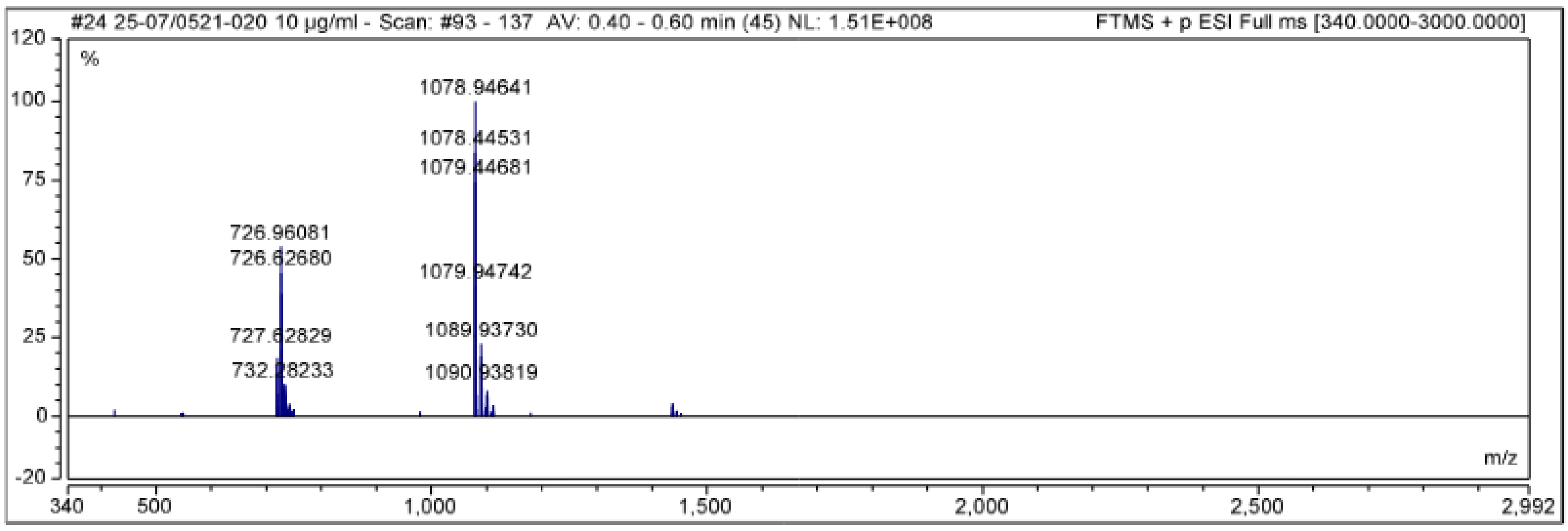

Radiolabeled bicyclic peptides

Exemplary radioactivity-detected chromatograms are shown below after labeling of the different peptides with copper-61, copper-64 or gallium-68. Labeling yields and purity were usually ≥95%.

[^{64}Cu]Cu-NECT-330

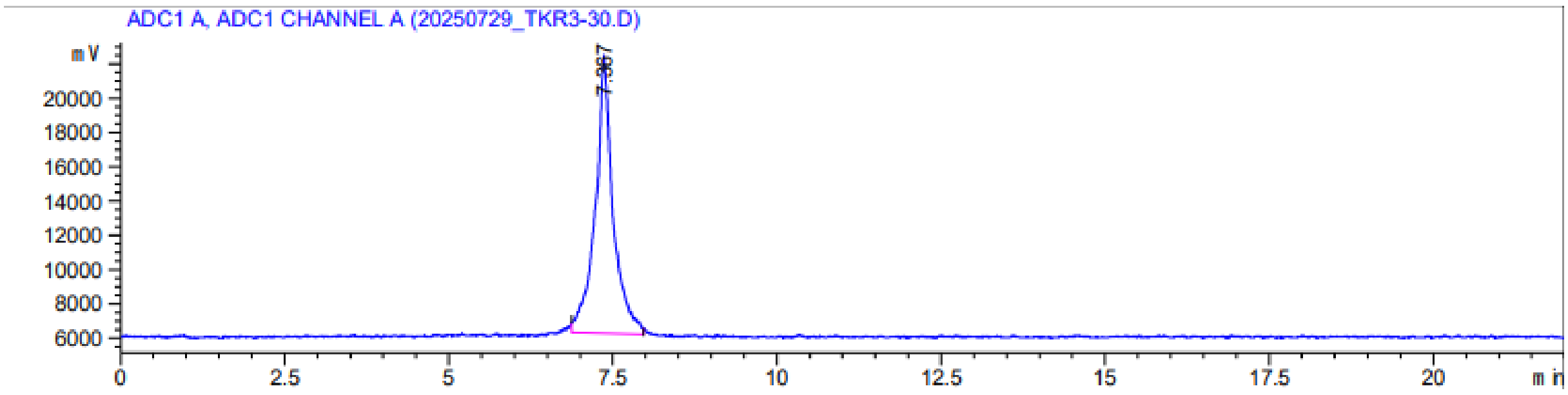

[^{64}Cu]Cu-NECT-334

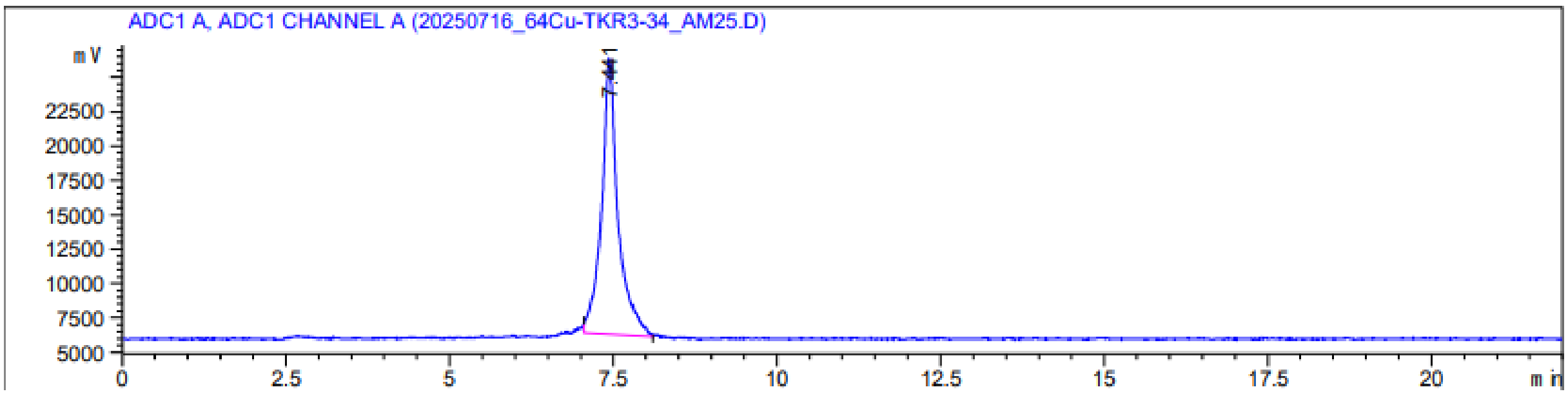

[^{64}Cu]Cu-NECT-346

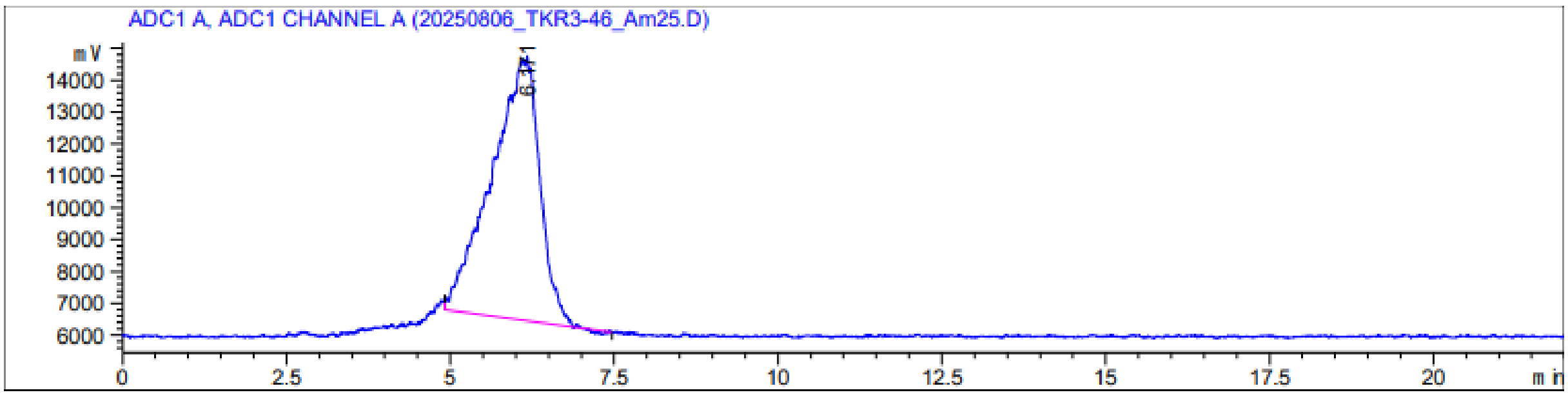

[^{68}Ga]Ga-NECT-346

The observed double peak likely arises from the cis/trans-isomerization of the ten tertiary amide bonds introduced by attaching ten sarcosine residues. This might result in different conformers that putatively exhibit a different retention behavior at the chosen stationary phase for analytical radio-HPLC.

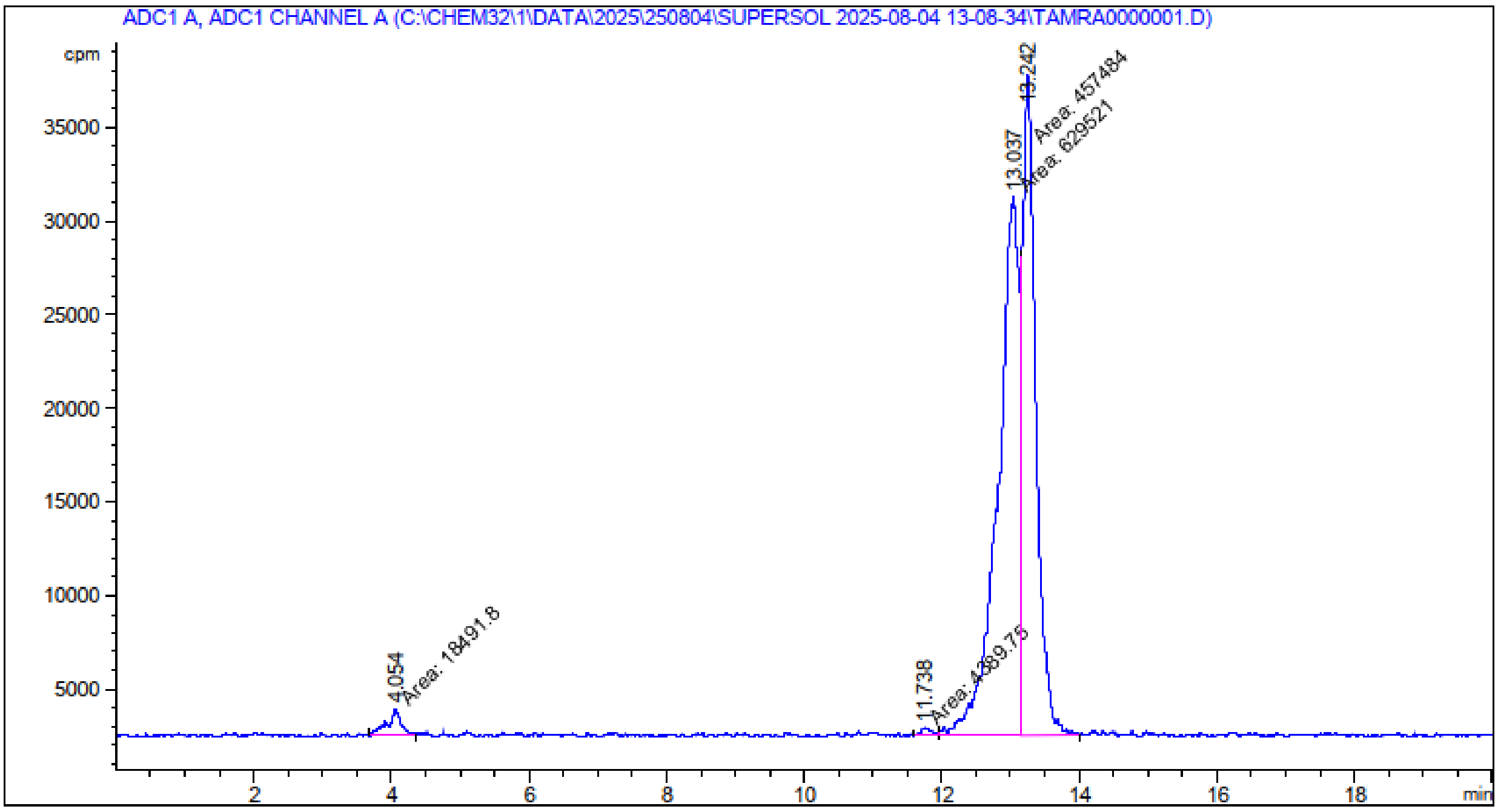

[^{61}Cu]Cu-NECT-346

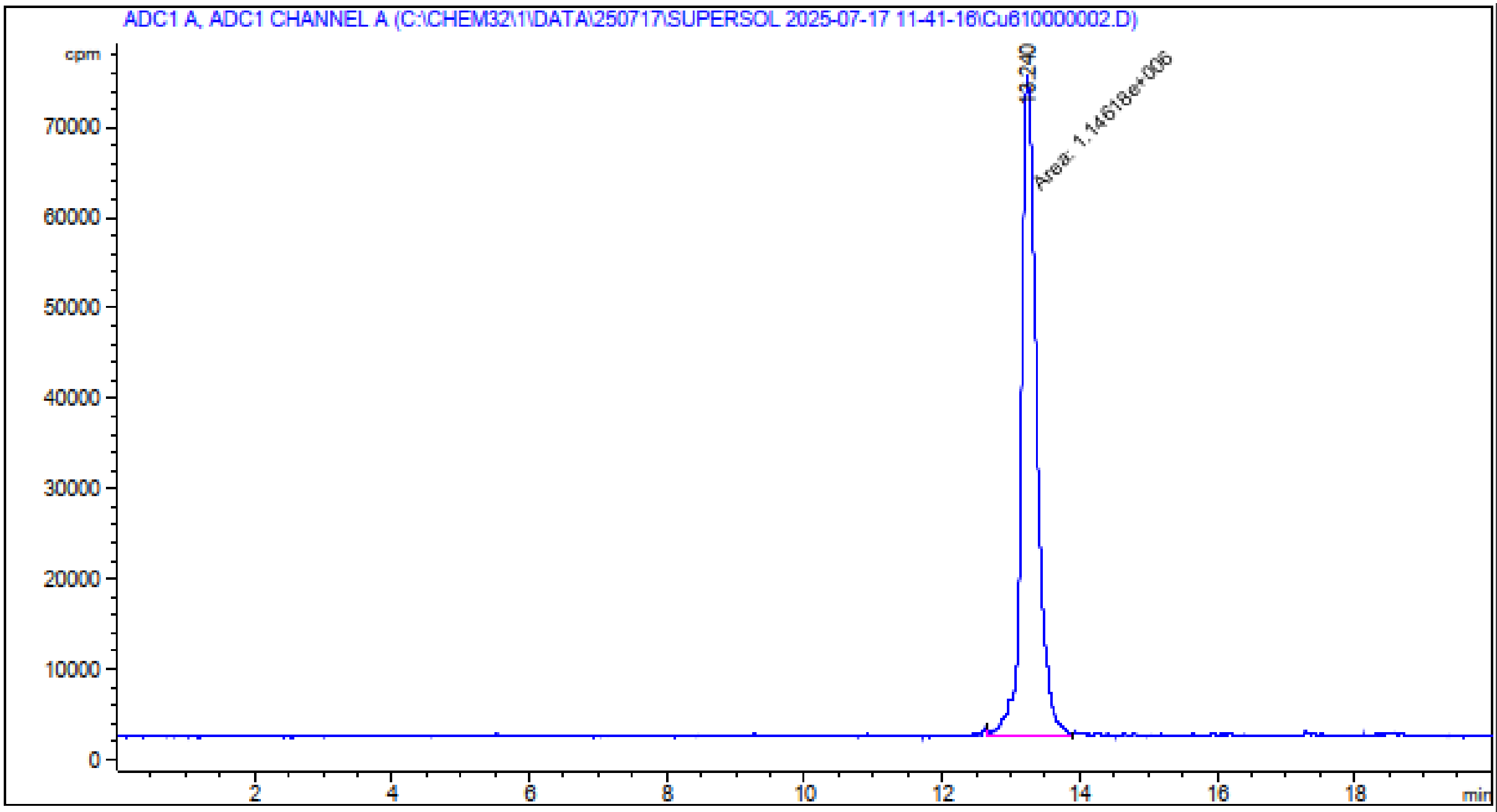

[^{61}Cu]Cu-NECT-224

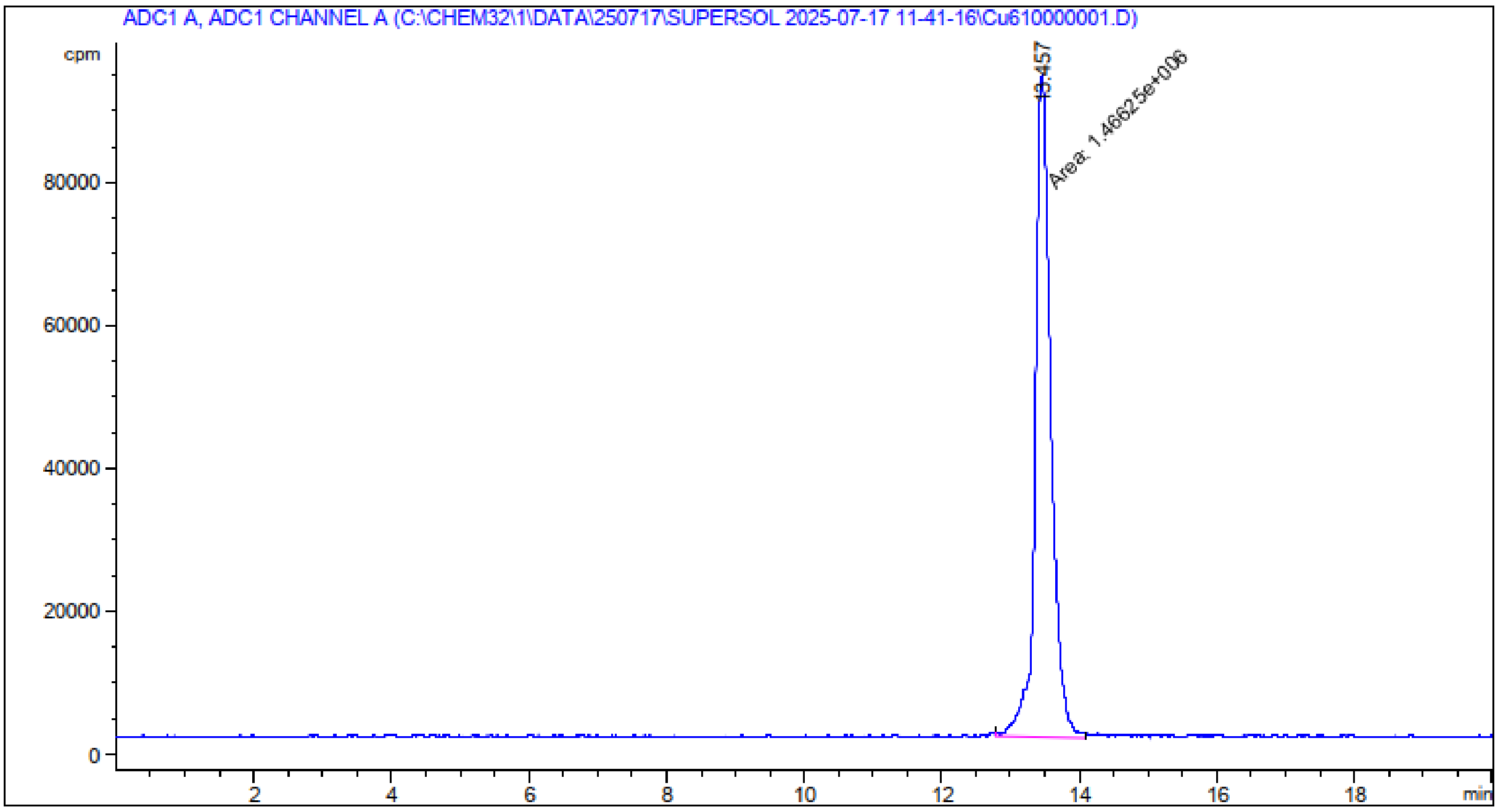

References

1. Brandt, F.; Ullrich, M.; Laube, M.; Kopka, K.; Bachmann, M.; Löser, R.; Pietzsch, J.; Pietzsch, H. J.; van den Hoff, J. and Wodtke, R. "Clickable" albumin binders for modulating the tumor uptake of targeted radiopharmaceuticals. *J. Med. Chem.*, **2022**, *65*, 710-733.
